# Synthesis and Activity‐Based Protein Profiling Identifies Aldo‐Keto Reductase 1C3 as Target Protein of Myxoglucamides

**DOI:** 10.1002/anie.6489568

**Published:** 2026-08-10

**Authors:** Thomas Siemon, Vivek K. Mishra, Mingming Zhao, Konrad Büssow, Alexander Popoff, Chengzhang Fu, Rolf Müller, Lothar Jänsch, Wulf Blankenfeldt, Mark Brönstrup

**Affiliations:** ^1^ Department of Chemical Biology Helmholtz‐Centre for Infection Research (HZI) Braunschweig Germany; ^2^ Department of Structure and Function of Proteins Helmholtz‐Centre for Infection Research (HZI) Braunschweig Germany; ^3^ Helmholtz‐Institute For Pharmaceutical Research Saarland (HIPS) Helmholtz Centre for Infection Research (HZI) and Department of Pharmacy (HZI), Saarland University, Campus E8.1 Saarbrücken Germany; ^4^ Research Group Cellular Proteome Research Helmholtz‐Centre For Infection Research (HZI) Braunschweig Germany; ^5^ Institute of Organic Chemistry and Centre of Biomolecular Drug Research (BMWZ) Leibniz Universität Hannover Hannover Germany; ^6^ German Center for Infection Research Site Hannover‐Braunschweig Braunschweig Germany

**Keywords:** activity‐based protein profiling, natural products, structure‐activity relationships, total synthesis, X‐ray crystallography

## Abstract

Myxoglucamides, natural products recently isolated from *Cystobacterineae* sp., are featured by an unprecedented vinyl‐substituted α‐keto‐γ‐amino acid that is linked to a glycosylated 14‐methyl‐pentadecanoic acid. Thus, they unite elements from three biomolecular classes in a compact glycolipopeptide. To elucidate the biological relevance of this arrangement, we searched for molecular targets by activity‐based protein profiling (ABPP)—although a phenotypic bioactivity has not been reported. An access to the compound class was established through the first total synthesis of myxoglucamide A in 11 steps. A proteome‐wide ABPP study led to the identification of aldo‐keto reductase 1C3 (AKR1C3) as the primary target of myxoglucamides in human cells. AKR1C3 is an oncogenic factor involved in prostaglandin and steroid synthesis, promoting the growth, proliferation, and metastasis of carcinoma cells. The functional inhibition of AKR1C3 by a competitive mechanism (IC_50_ = 1.61 µm) was validated in vitro, and 20 analogs provided structure‐activity relationships and more potent analogs (IC_50_ = 181 nm). Biophysical interactions were quantified by thermal shift assays, and essential molecular protein‐ligand interactions were characterized by X‐ray crystallography at 2.0 Å resolution. The study implies that the search for targets of natural products is rewarding even in the absence of an initial phenotypic activity.

## Graphical abstract

Myxoglucamide A—a novel glycolipopeptide isolated from myxobacteria—was synthesized, and activity‐based protein profiling identified aldo‐keto reductase 1C3 (AKR1C3) as its main target in human cells. Structure‐activity relationship studies, together with biophysical characterization and X‐ray crystallography, elucidated the structural basis of AKR1C3 inhibition.

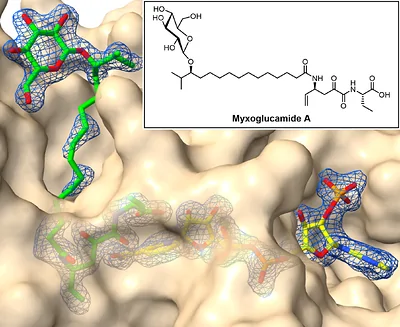

## 1 Introduction

The study of natural products has been highly beneficial for the progression of biological research [1, 2, 3]. They have not only been a highly prolific source of new drugs until today [4], but also disclosed original cellular targets and mechanisms that were subsequently exploited therapeutically by the natural product itself, or by other molecular formats [5]. The starting point of cellular mode‐of‐action studies is usually a strong phenotypic activity, such as the inhibition of growth of a cancer cell or of pathogenic microbes. However, the absence of a strong phenotype induced by a natural product and the limited availability of phenotypic assays leaves the biological function of the compound undisclosed in the majority of cases—and this uncharted biological space is regarded as one of the most eminent problems in today's natural product research [6].

We followed the hypothesis that an evolutionary optimization of complex natural products, whose production is metabolically costly, must serve a function. Thus, we set out to elucidate the bioactivity of natural products even in the absence of a cell death phenotype. Instead, original chemical structures of metabolites and an underexplored habitat were main selection criteria for a mechanistic study, as recently exemplified by our study of the volatile marine salinilactones [7]. The current study exploits the bioactivity of secondary metabolites produced by myxobacteria, Gram‐negative species predominantly found in soil that are characterized by their complex life cycles, social behaviors such as cooperative hunting, fruiting body formation, and cellular differentiation [8]. These microorganisms express a diverse array of natural products with significant pharmacological potential in multiple indications [9]. To further uncover the enormous genomic potential of myxobacteria [10], Wang et al. followed a genome mining strategy [11] that led to the discovery of a novel family of natural products isolated from *Cystobacterineae* sp. MCy9003 and named myxoglucamides (Figure 1) [12]. As shown in this issue of Angew Chem, the structure of myxoglucamide A (**1**) features an unprecedented γ‐amino acid‐containing α‐ketoamide with a vinylic side chain, linked to 13β‐d‐glucopyranosyl‐14‐methyl‐pentadecanoic acid. Because the myxoglucamides combine elements of three major biomolecular classes (lipids, sugars, peptides) in a compact molecule, we aimed to find out the bioactivity exerted by this arrangement by applying an unbiased activity‐based protein profiling (ABPP) approach. We speculated that the myxoglucamides bind covalently to their target(s) through the α‐ketoamide, given that this moiety is a widespread electrophilic warhead in natural and synthetic peptide‐based drugs. Herein, we developed a total synthesis of myxoglucamides that enabled the identification and validation of their mammalian target.

**FIGURE 1:**
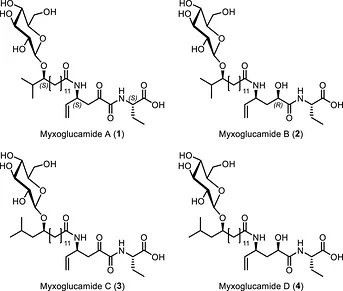
Structure of myxoglucamides.

## 2 Results and Discussion

For an unbiased target identification of myxoglucamides using activity‐based protein profiling (ABPP) with chemical probes [13, 14, 15, 16], a synthetic access to the natural products needed to be established first. We chose myxoglucamide A (**1**) as the target molecule for a total synthesis. Our retrosynthetic considerations resulted in a convergent strategy in which a glycosylated fatty acid (**II**) and a peptidyl building block (**III**) would be coupled in the final steps (Scheme 1A). The peptide fragment was accessible using commercially available, enantiomerically pure starting materials derived from amino acids (**IV** and **V**). For the fatty acid building block, we planned to install the chiral hydroxy group via enantioselective synthesis.

**SCHEME 1:**
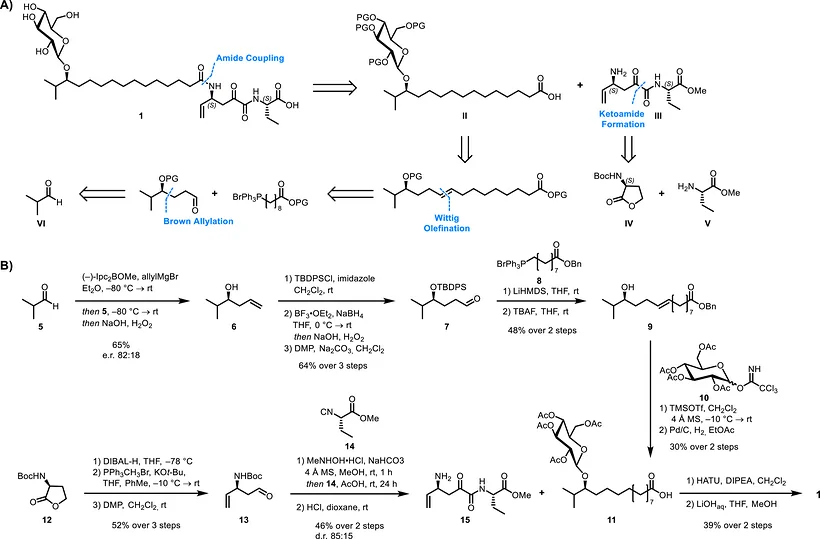
(A) Retrosynthetic analysis of myxoglucamide A (**1**), (B) Synthesis of **1**. PG = protecting group, Boc = *tert*‐butyloxycarbonyl, Ipc = isopinocampheyl, TBDPS = *tert*‐butyldiphenylsilyl, THF = tetrahydrofuran, DMP = Dess‐Martin periodinane, HMDS = bis(trimethylsilyl)amine, TBAF = tetrabutylammonium fluoride, TMSOTf = trimethylsilyl trifluoromethanesulfonate, HATU = *O*‐(7‐azabenzotriazol‐1‐yl)‐*N*,*N*,*N′*,*N′*‐tetramethyluronium hexafluorophosphate, DIPEA = *N*,*N*‐diisopropylethylamine.

A Brown allylation [17] of isobutyraldehyde (**5**) furnished the (*S*)‐allylic alcohol **6** with an enantiomeric ratio of 82:18, as determined by Mosher ester analysis [18] (Scheme 1B and Supporting Information). After silyl protection of the alcohol, the terminal olefin was subjected to hydroboration‐oxidation to yield the aldehyde **7**. A subsequent Wittig reaction with the phosphonium benzyl‐nonanoate **8** extended the carbon chain to the desired length. Following silyl deprotection, glycosylation of the resulting alcohol (**9**) was achieved using Schmidt's method with trichloroacetimidate **10** to selectively install the desired β‐d‐glycosyl moiety [19]. The synthesis of the fatty acid building block **11** was completed by simultaneous hydrogenation of the alkene and cleavage of the benzyl ester.

The synthesis of the peptide fragment started from commercially available Boc‐protected homoserine lactone (**12**). Reduction with diisobutylaluminium hydride (DIBAL‐H), followed by Wittig methylenation and Dess‐Martin oxidation afforded aldehyde **13** [20, 21]. Subsequently, the α‐ketoamide **15** was directly accessible via an oxidative coupling with isocyanide **14**. We applied a method developed by Grassot et al. [22]. that involved the condensation of *N*‐methyl‐hydroxylamine with aldehyde **13**, followed by an Ugi‐type reaction with isocyanide **14** and acetic acid, that led to an isolable α‐acetoxyamino amide intermediate. Mediated by 4 Å molecular sieves (4 Å MS), β‐elimination and hydrolysis resulted in the formation of α‐ketoamide **15**. Finally, amide coupling with the fatty acid fragment **11**, followed by global deprotection, afforded myxoglucamide A (**1**). The synthesis of **1** was achieved in 11 steps in the longest linear sequence with an overall yield of 2.4% and 16 steps in total.

To enable ‘target fishing’ by ABPP, myxoglucamide was linked to biotin. The design of the activity‐based probe **16** (Figure 2A) [15, 16] was built on the hypothesis that the isopropyl group of **1** was least involved in target binding and could be replaced with an alkyne as a handle for click chemistry. The synthesis of the alkynylated fatty acid started with the epoxidation of ethyl 13‐tetradecenoate (**17**), followed by Jacobsen hydrolytic kinetic resolution, to obtain the enantiopure epoxide **18** [23]. Epoxide opening with lithiated (triisopropylsilyl)acetylene provided the desired (*S*)‐configured alcohol with >99% ee as confirmed by Mosher ester analysis. After hydrolysis and silyl deprotection, carboxylic acid **19** was directly coupled with amine **15**. At this stage, the Schmidt glycosylation proved to be difficult, resulting in very low yield due to incomplete reaction and partial transacetylation. Nevertheless, the synthesis of the activity‐based probe **16** was completed via a copper‐catalyzed azide–alkyne cycloaddition with biotin–PEG_3–_azide [24].

**FIGURE 2:**
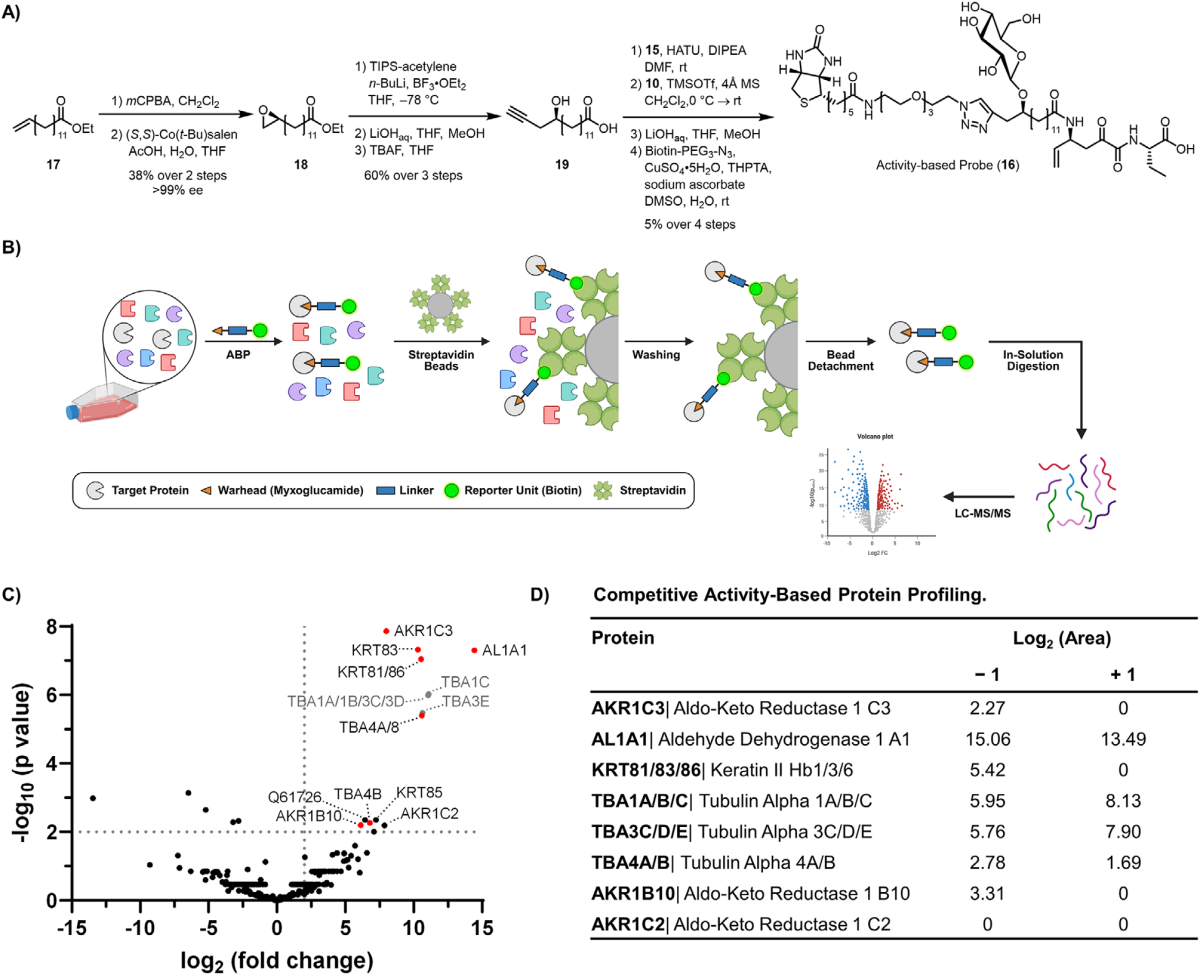
Target identification by ABPP. (A) Synthesis of activity‐based probe (ABP) **16**. (B) ABPP workflow. Graphic created with BioRender. (C) Volcano plot of proteins identified by tandem mass spectrometry after enrichment by ABPP in A549 lung epithelial cell lysate (positive control: n = 5, negative control: n = 5). The thresholds for significantly enriched proteins were set to *p* = 0.01 and log_2 _(fold change) = 2 and visualized with dashed grey lines. (D) Competitive ABPP. A549 cell lysate was incubated with **1** (n = 3) or DMSO (n = 3) for 30 min prior to the addition of ABP **16**. The resulting log2‐transformed peak areas of the previously identified proteins are listed in (D). Proteins that were significantly enriched and showed binding to **1** in the competitive ABPP are labeled as red dots in (C). Proteins that exhibited a higher enrichment after preincubation with **1** are marked in gray. *m*CPBA = *meta*‐chloroperoxybenzoic acid, TIPS = triisopropylsilyl, DMF = *N*,*N*‐dimethylformamide, PEG = polyethylene glycol, THPTA = tris(3‐hydroxypropyltriazolylmethyl) amine, DMSO = dimethylsulfoxide.

The anomeric center of **16** was β‐configured, as shown by a doublet at 4.27 ppm with a characteristic coupling constant of *J*  =  7.9 Hz in the ^1^H NMR spectrum.

To search for human protein targets of myxoglucamides, we selected the well‐characterized A549 lung epithelial cell line as a model system for ABPP, also because it is cultivated in a reproducible manner in our hands. Probe **16** was incubated with an A549 cell lysate for 3 h (Figure 2B). In parallel, lysates treated with DMSO served as negative controls to account for nonspecific binding artifacts. A pulldown of probe‐bound targets was performed using streptavidin‐coated Sepharose beads, leveraging the high affinity between biotin and streptavidin. After washing and centrifugation steps to remove unbound material, probe‐bound proteins were eluted under denaturing conditions. For their identification by nano‐liquid chromatography coupled tandem mass spectrometry (nano‐LC‐MS/MS), the samples were prepared using an adapted SP3 (single‐pot, solid‐phase‐enhanced sample preparation) protocol [7, 25]. The resulting MS/MS data were analyzed against a human protein database and visualized in a volcano plot (Figure 2C). Several proteins exhibited significant enrichment compared to the negative control, with the highest observed for aldehyde dehydrogenase 1A1 (AL1A1). The aldo‐keto reductase 1C3 (AKR1C3) demonstrated the strongest statistical significance, reflected by the highest –log_10_(p) value. Notably, AKR1B10 and AKR1C2, two other members of the aldo‐keto reductase family, were significantly enriched. In addition to these oxidoreductases, several tubulin α‐chains (1A–C, 3C–E, 4A–B, and 8) and keratins (type II Hb1/3/5/6) were identified.

In order to confirm the specific binding of **16** to these proteins, we performed a competitive ABPP. For this purpose, A549 cell lysates were pre‐incubated with an excess of **1** (or DMSO as a negative control) for 30 min. Subsequently, ABP **16** was added to all samples, and the previously described ABPP workflow was carried out. Proteins specifically bound by myxoglucamides were then indicated by a decrease in their peak abundances following pretreatment with **1**. The proteins AKR1C3, AKR1B10 and keratins II Hb1/3/6 (KRT81/83/86) were no longer enriched after pre‐incubation, indicating complete saturation of the binding site by **1** (Figure 2D). AL1A1 was detectable in both conditions, but slightly less abundant under pre‐treatment conditions. A specific binding to tubulin‐α chains, except for TBA4A/B, could not be confirmed in the competition experiment.

In summary, the chemoproteomics data implied that myxoglucamides may target keratin and in particular oxidoreductases. We decided to focus further investigations on the latter. In order to validate the ABPP results, we probed whether **1** had any functional effect on the identified oxidoreductases. This was tested by six enzymatic in vitro assays that measured the catalytic conversion of model substrates in the presence of **1** by quantifying the cofactor NADPH. Aldehyde dehydrogenase 1A1 was not inhibited by **1** without pre‐incubation or after just 1 h of pre‐treatment. However, an IC_50_ value of 210 µm was observed after 6 h of pre‐incubation (Table 1). These results were consistent with the competitive ABPP data, where only a moderate reduction in enrichment was seen following the short pre‐incubation period. Since several aldo‐keto reductases have been identified by ABPP, we also investigated the inhibitory activity of **1** against the structurally related family members AKR1B1, AKR1C1, although these were not detected in the ABPP experiment. AKR1B1 and AKR1B10 were only inhibited by very high concentrations of **1**, while AKR1C1 and AKR1C2 showed no inhibition even at the highest tested concentration (IC_50_> 250 µm). In contrast, AKR1C3 was strongly inhibited by **1** with an IC_50_ value of 1.61 µm. Aldo‐keto reductase family 1 member C3 (AKR1C3; aka 17 β‐HSD5) is an NADP^+^/NADPH‐dependent oxidoreductase that plays a crucial role in steroid metabolism by catalyzing the final reduction step in the biosynthesis of testosterone and 17β‐estradiol (Scheme S1) [26]. Additionally, AKR1C3 functions as a prostaglandin F2α synthase [27]. Overexpression of AKR1C3 has been observed in various types of cancer (e.g., prostate, breast, lung cancer, leukemia) and acts as an oncogenic factor, promoting the growth, proliferation, and metastasis of carcinoma cells [28].

**TABLE 1:** Evaluation of inhibitory activity of **1** against identified target proteins.

Protein	IC_50_ [µm]
**AL1A1**| Aldehyde Dehydrogenase 1 A1	210 ± 7^a^
**AKR1B1**| Aldo‐Keto Reductase 1 B1	221 ± 36^b^
**AK1B10**| Aldo‐Keto Reductase 1 B10	348 ± 136^b^
**AKR1C1**| Aldo‐Keto Reductase 1 C1	> 250^c^
**AKR1C2**| Aldo‐Keto Reductase 1 C2	> 250^c^
**AKR1C3**| Aldo‐Keto Reductase 1 C3	1.61 ± 0.14^c^

^a^ NAD^+^/MTT‐coupled oxidation of acetaldehyde, 6 h preincubation with **1**.

^b^ NADPH‐coupled reduction of dl‐glyceraldehyde.

^c^ NADP^+^‐coupled oxidation of (*S*)‐tetrahydronaphthol. MTT = (3‐(4,5‐dimethylthiazol‐2‐yl)‐2,5‐diphenyltetrazoliumbromid).

AKR1C3 has also been investigated as a treatment of endometriosis [27]. Therefore, the discovery and development of selective inhibitors has become an area of intense research that led to the identification of natural products, steroids, nonsteroidal anti‐inflammatory drugs (NSAIDs), fatty acids, organic ruthenium complexes, off‐label drugs, other small‐molecule heterocycles, or proteolysis‐targeting chimeras (PROTACs) [28, 29, 30, 31]. Clinical studies with the steroidal BAY1128688 and the indol ASP9521 were initiated, but discontinued due to off‐target toxicity or lack of efficacy [28].

Based on the selectivity of **1** for AKR1C3 and its relevance as a therapeutic target, we focused our efforts on this protein to establish structure–activity relationships (SAR) of myxoglucamids (Table 2). We found that the glucose moiety was not essential for inhibition, as the corresponding aglycon **20** was more potent. Consequently, the role of the fatty acid substituent was investigated further. Complete removal of the alkyl chain as in **21** or substitution with only short alkyl groups as in **22** significantly reduced activity.

**TABLE 2:** Structure‐activity relationship study of aldo‐keto reductase 1 C3 (AKR1C3).

#	R	IC_50_ [µm]^b^	CC_50_ [µm]^c^	SI^d^	#	R	IC_50_ [µm]^b^	#	R	IC_50_ [µm]^b^	CC_50_ [µm]^c^	SI^d^
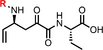					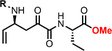			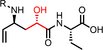				
**1**	**R1**	1.61	> 100	> 62	**33**	**R2**	22.8	**37**	**R1**	0.501	> 100	> 199
**20**	**R2**	0.947	> 100	> 106				**38**	14:0^a^	0.314	40.4	129
**21**	C(O)O*t‐*Bu	> 128	n.d.		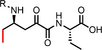			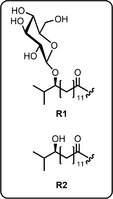				
**22**	6:0^a^	15.6	n.d.									
**23**	10:0^a^	0.855	> 100	> 116	**2**	**R1**	> 64					
**24**	14:0^a^	0.344	55.9	163	**34**	14:0^a^	14.0					
**25**	18:0^a^	0.573	25.7	45								
**26**	16:1*^cis^* ^−9^ ^a^	0.181	53.6	296	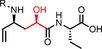							
**27**	18:1*^cis^* ^−9^ ^a^	0.208	32.5	156								
**28**	22:1*^cis^* ^−13^ ^a^	1.54	21.8	14	**35**	**R1**	47.5					
**29**	18:2*^cis−^* ^9,12^ ^a^	0.242	51.6	213								
**30**	18:3*^cis^* ^−9,12,15^ ^a^	0.196	59.3	303	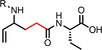							
**31**	20:4*^cis−^* ^5,8,11,14^ ^a^	0.432	44.2	102								
**32**	22:6*^cis−^* ^4,7,10,13,16,19^ ^a^	0.246	42.3	172	**36**	14:0^a^	22.6					

^a^ Fatty acid—number of carbon atoms: double bonds *^Configuration^* ^‐Location.^

^b^ Mean of n  =  3, AKR1C3: 132 nm, (*S*)‐tetrahydronaphthol: 1 mm, NADP^+^: 0.5 mm.

^c^ Cytotoxicity in A549 cells determined by Alamar Blue assay. Mean of n = 3.

^d^ Selectivity Index.

In contrast, longer fatty acid chains markedly improved potency, reaching nanomolar IC_50_ values (**23‐25**). The strongest inhibition was observed with unsaturated fatty acid substituents (**26‐32**), consistent with prior reports that AKR1C3 is potently inhibited by unsaturated fatty acids [32]. We next examined the importance of the carboxylic acid functionality. Its conversion into a methyl ester (**33**) led to a >20‐fold decrease in activity. A similar trend was observed for the ketoamide moiety: reduction (**2**, **34**, **35**) or removal (**36**) of the carbonyl group resulted in a significant loss of potency.

Interestingly, saturation of the vinyl group had no observable effect on inhibitory activity (**37**, **38)**.

Next, we evaluated the cytotoxic effect of myxoglucamide A and its analogs on A549 cells. While **1** showed no cytotoxicity up to a concentration of 100 µm, several of its derivatives displayed CC_50_ values ranging from 20 to 60 µm. However, the ratio between the concentration required for enzymatic inhibition and for cellular cytotoxicity, termed selectivity index (SI) here, was still high. The only analogs with an SI below 100 were **25** and **28**, which feature the longest alkyl chains with the lowest degree of unsaturation. In the enzyme inhibition assay, these derivatives achieved only 60–70% inhibition, even at very high concentrations, suggesting potential solubility issues. These solubility limitations are likely also a contributing factor to their increased cytotoxicity. Moreover, the fact that the potency of AKR1C3 inhibition did not correlate with cytotoxicity speaks against an on‐target effect, but for a lipophilicity‐related off‐target phenomenon.

To gain a deeper understanding of the mechanism of AKR1C3 inhibition, we conducted an enzyme kinetic study. Myxoglucamide A exhibited competitive inhibition relative to the substrate (*S*)‐tetrahydronaphthol, as evident from the Michaelis‐Menten and Lineweaver‐Burk plots (Figure 3A,B). Additionally, we determined the substrate‐independent inhibition constant (K_i_) for **1** and the most active analogs **24**, **26,** and **30**. As these compounds operate in the tight‐binding regime, conventional Michaelis–Menten‐based analyses are not applicable. Therefore, K_i_ values were determined using the Morrison equation, yielding inhibition constants in the low‐nanomolar range, with compound **26** displaying a K_i_ value of only 20 nm (Figure 3F).

**FIGURE 3:**
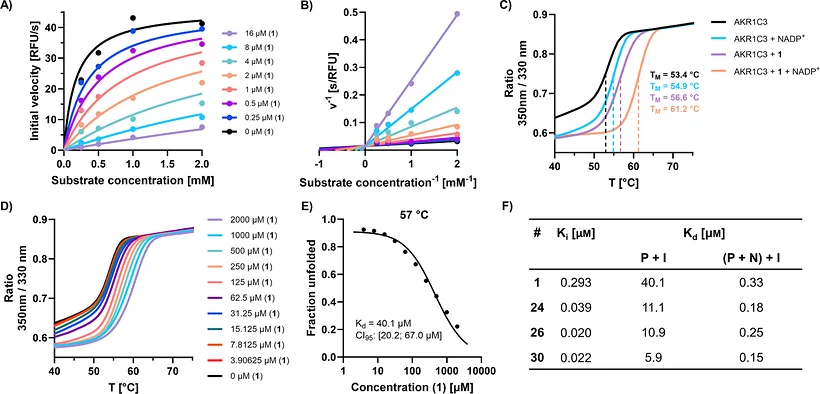
Michaelis‐Menten enzyme kinetic study and thermal shift assays for the binding of **1** to AKR1C3. (A) Michaelis‐Menten enzyme kinetics study for the inhibition of AKR1C3 by **1**. Competitive inhibition fit performed using GraphPad Prism 10.3.0. Conditions: AKR1C3: 132 nm, (*S*)‐tetrahydronaphthol: 0.25 – 2 mm, **1**: 0 – 16 µm, NADP^+^: 0.5 mm. (B) Lineweaver‐Burk plot of enzyme kinetics measurement from (A). (C) Thermal shift assay (nanoDSF) for T_M_ determination of binary and ternary complexes between AKR1C3, **1** and NADP^+^. AKR1C3 (12 µm) was incubated with or without 200 µm of **1** and NADP^+^, each. (D) Thermal shift assay of AKR1C3 (13 µm) with increasing concentrations of **1**. (E) Fraction of unfolded protein was calculated from D) using “FoldAffinity” [33, 34] at a selected temperature of 57°C and plotted against the concentration of **1**. K_d_ values were estimated and are listed in F) as the mean of n = 3. (F) Inhibition constants K_i_ and dissociation constants K_d_ for selected myxoglucamide analogs. K_i_ values (mean of n  =  2) were determined from Michaelis‐Menten kinetics and fitted with GraphPad Prism 10.3.0 using the Competitive Inhibition model for **1**, and the Morrison K_i_ model for inhibitors **24**, **26** and **30**. Conditions: AKR1C3: 132 nm, (*S*)‐tetrahydronaphthol: 0.25 – 2 mm, NADP^+^: 0.5 mm. **P** = AKR1C3, **I** = Inhibitor, **N** = NADP^+^.

Next, we investigated the binding affinity of **1** to AKR1C3. For this purpose, thermal shift assays using nano differential scanning fluorimetry (nanoDSF) were employed that measured the intrinsic fluorescence of the protein at increasing temperatures. Ligand binding typically stabilizes the protein, resulting in an increase in the denaturation temperature (T_M_). We determined the T_M_ shift of the binary complexes **1** – AKR1C3 and NADP^+^—AKR1C3 as well as the ternary complex. The T_M_ shift of the ternary complex was larger than the sum of the individual T_M_ shifts, indicating a positive cooperative effect on protein stabilization (Figure 3C and Table S6).

To further study this effect, we estimated the dissociation constant (K_d_), by measuring the thermal shift with increasing ligand concentrations (Figure 3D). By fitting the fraction of unfolded protein, obtained by the FoldAffinity method developed by Niebling et al. [33], against the initial ligand concentration, the K_d_ value can be roughly estimated at temperatures near the T_M_. From the thermal shift assay of the binary complex of **1** with AKR1C3, we obtained a K_d_ value of 40 µm (Figure 3E) [34]. The same methodology was applied for the ternary complex and resulted in an estimated K_d_ value of 0.33 µm. Thus, a tight binding of **1** is only achieved in the presence of the cofactor. In line with the SAR study, the K_d_ values of the most active derivatives **24**, **26**, and **30** were approximately two to four times lower than that of **1** (Figures 3F and S7).

Finally, we were able to obtain an X‐ray cocrystal structure of AKR1C3 in complex with **1** and NADP^+^ at a resolution of 2.0 Å (Figure 4). The protein crystallized as a dimer with myxoglucamide bound in both monomers. While the electron density of the peptide part was well‐defined and consistent in both chains, the glucose moiety was observed only in chain B with reasonable electron density (Figures 4A and S8). This is likely due to the high flexibility of the alkyl linker, and it reflects our observation that a glycosylated chain is not required for activity. AKR1C3 features a large and very hydrophobic active site that has been divided into the following subsections: the oxyanion site formed by the catalytic residues Tyr55, His117 and NADP^+^, a steroid channel (Tyr24, Leu54, Ser129, Trp227) and the three sub‐pockets SP1 (Ser118, Asn167, Phe306, Phe311, Tyr319), SP2 (Trp86, Leu122, Ser129, Phe311) and SP3 (Try24, Glu192, Ser221, Tyr305) [35]. The carboxylic acid of **1** was located at the oxyanion site, forming key hydrogen bonds to the catalytic residues Tyr55 (2.6 Å) and His117 (3.0 Å). This is in line with the observation that the carboxylic acid was essential for AKR1C3 inhibition (Figure 4B). The α‐ketoamide did not participate in any hydrogen bonding or covalent interactions. However, it rigidified the peptidyl moiety by favoring an almost planar conformation due to the antiperiplanar alignment of the adjacent carbonyl groups (Figure 4B). The vinyl group and the adjacent amide extended into the SP1 pocket, with the amide nitrogen atom forming a hydrogen bond to Ser118. Compared to the other AKR1C isoforms, the SP1 site of AKR1C3 is significantly larger, primarily due to a shift in the backbone conformation of residues 305–311 (Figure 4C).

**FIGURE 4:**
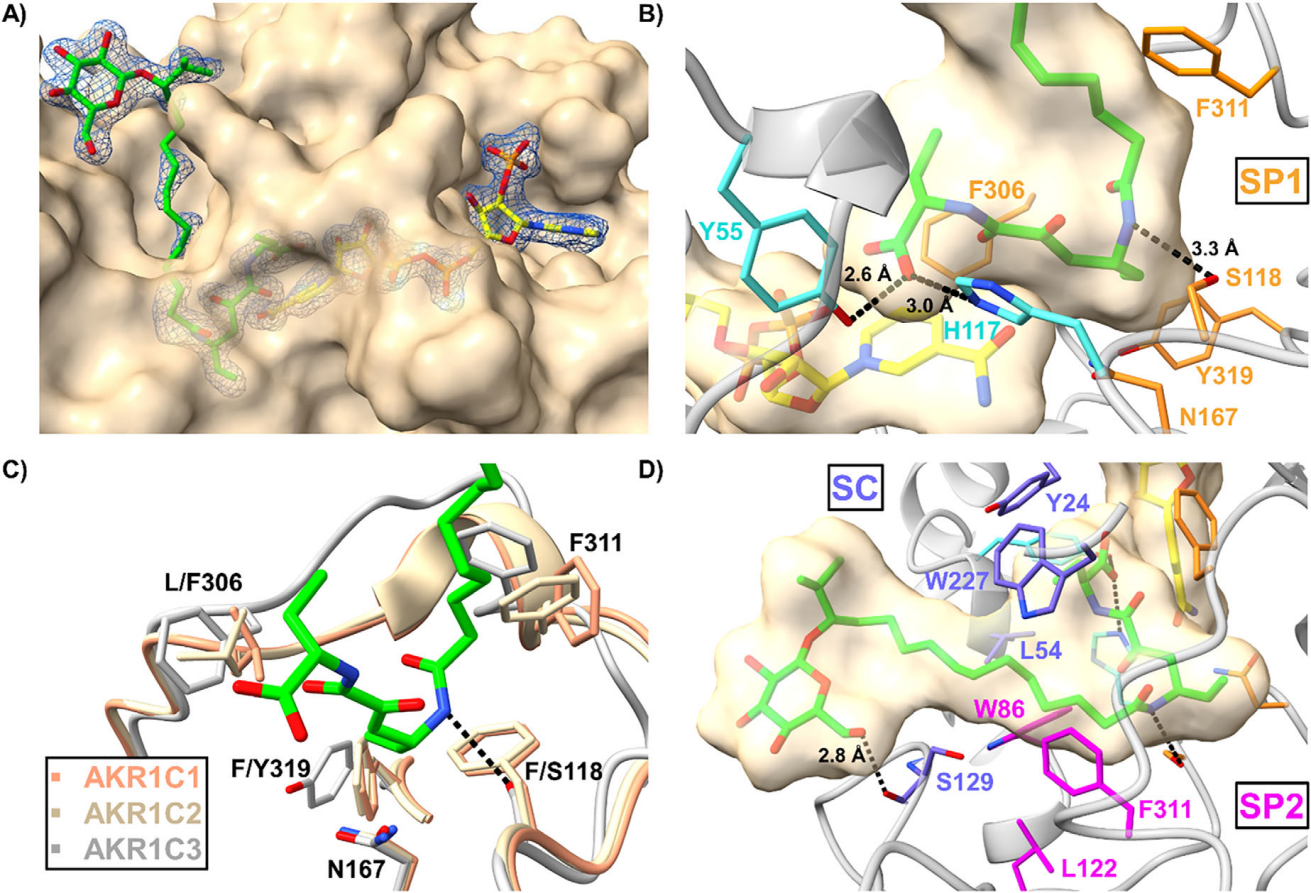
X‐ray crystal structure of AKR1C3 in complex with **1** and NADP^+^ (PDB: 9SWH). (A) 2F_o_–F_c_ electron density map of **1** and NADP^+^ contoured at 1.0 σ, shown as blue mesh within the AKR1C3 surface of chain B; **1** in green and NADP^+^ in yellow sticks. (B) Peptidyl binding site. The residues of the subsections are color‐coded: cyan = oxyanion site, orange = SP1. Hydrogen bonding interactions are shown as dashed lines. (C) Superposition of the SP1 pockets of AKR1C1 (PDB: 3NTY) [36], AKR1C2 (PDB: 4XO7) [37], and AKR1C3. (D) Localization of the glycosylated alkyl moiety. The residues of the subsections are color‐coded: magenta = SP2, purple = steroid channel (SC).

Additionally, Ser118 is replaced by phenylalanine in the other AKR1C isoforms, eliminating the potential for hydrogen bonding to the amide group. These differences likely contributed to the high selectivity of **1** toward AKR1C3. The alkyl chain was partially solvent‐exposed as it extended through the SP2 pocket and the steroid channel (Figure 4D). The glucose moiety was located outside the binding site and formed a hydrogen bond to the amide carbonyl of Ser129 at the protein surface, which explains its minimal impact on AKR1C3 inhibition.

## 3 Conclusion

The unique glucolipopeptide structure of myxoglucamides prompted us to shed light on their biological role. To identify its molecular target in A549 human lung epithelial cells, a synthetic access to the compound class was a prerequisite. It was accomplished by the first total synthesis of myxoglucamide A in 11 steps in the longest linear sequence with an overall yield of 2.4%, employing a convergent strategy with a late‐stage coupling between the peptidic and the glycolipidic building blocks. The synthesis enabled activity‐based protein profiling with a myxoglucamide‐biotin conjugate that led to the identification of several oxidoreductases as well as structural proteins as binders. The aldo‐keto reductase 1C3 (AKR1C3) was validated as a target of **1** through functional, biophysical and structural studies.

The synthetic access also allowed studying the structure‐activity relationship of AKR1C3 inhibition with the help of 20 analogs. The glucose moiety was not essential for inhibition. It might therefore rather function as an enhancer of solubility in the lipophilic part of **1** or influence chemical stability, cellular uptake, transport, or interactions with biological targets in the native host or competing microorganisms [38]. The lipid part, whose truncation and unsaturation led to an eightfold enhanced potency, occupied the hydrophobic SP2 pocket and extended through the steroid channel.

Our initial assumption that the α‐ketoamide functions as a covalent warhead was incorrect, as AKR1C3 is lacking a reactive cysteine residue in its active site. However, the SAR study indicates that the ketoamide is essential for biological activity. Given the lack of bonding interactions, it might rather enhance the structural rigidity of the peptidyl fragment in a defined conformation [39]. Also, the carboxylic acid was found to be essential for inhibition by binding to parts of the catalytic tetrad (His117 and Tyr55); similar interactions have been observed for synthetic AKR1C3 binders as well. The peptidic part of **1** thus was essential to occupy the catalytic site of the enzyme, stabilizing the ternary complex formed between AKR1C3, **1**, and NADP^+^. These structure‐activity relationships map the roles of the peptide, lipid, and sugar parts of the myxoglucamides with respect to AKR1C3 inhibition. The findings further illustrate that an α‐ketoamide moiety is not necessarily associated with covalent binding, as demonstrated by other natural products such as rapamycin, complestatin, or plitidepsin.

Because AKR1C isoforms show variable and partially undesired bioactivities, a high selectivity of inhibitors for AKR1C3 is regarded as important [29]; this criterion is fulfilled by the myxoglucamides. They have not reached the low nanomolar potency of the best, optimized AKR1C3 inhibitors yet, but provide a new and original starting point against this well‐validated target.

While AKR1C3 has been identified as an important mammalian target of myxoglucamides, the effect(s) of the compound in their natural habitat and associated targets in microbial communities remain to be deciphered in future studies. Given the lack of a strong growth inhibition phenotype, a role in microbial signaling or interspecies interactions is conceivable. On a general notion, the study demonstrates the merit of investigating natural products even in the absence of strong phenotypes such as cell death induction. The use of unbiased chemoproteomic methods was a viable strategy to alleviate the pronounced darkness of biological space targeted by natural products that appear ‘inactive’ at first sight.

## Author Contributions

**Thomas Siemon**: writing – original draft, investigation, formal analysis, methodology, conceptualization. **Vivek K. Mishra**: investigation, writing – review and editing. **Mingming Zhao**: investigation, writing – review and editing. **Konrad Büssow**: investigation, writing – review and editing. **Alexander Popoff**: writing – review and editing, investigation. **Chengzhang Fu**: writing – review and editing, investigation. **Rolf Müller**: writing – review and editing, conceptualization. **Lothar Jänsch**: supervision, investigation, writing – review and editing, resources, validation. **Wulf Blankenfeldt**: supervision, formal analysis, resources, validation. **Mark Brönstrup**: supervision, writing – original draft, writing – review and editing, conceptualization, formal analysis, resources, validation.

## Conflicts of Interest

The authors declare no conflicts of interest.

## Supporting information



The authors have cited additional references within the Supporting Information [40, 41, 42, 43, 44, 45, 46, 47, 48, 49, 50, 51, 52, 53, 54, 55, 56, 57].**Supporting File**: anie73999‐sup‐0001‐SuppMat.docx.

## Data Availability

The data that support the findings of this study are available in the Supporting Information of this article.

## References

[1] S. B. Bharate and C. W. Lindsley , “Natural Products Driven Medicinal Chemistry,” Journal of Medicinal Chemistry 67 (2024): 20723–20730, 10.1021/acs.jmedchem.4c02736. 39629819

[2] A. L. Harvey , R. Edrada‐Ebel , and R. J. Quinn , “The Re‐Emergence of Natural Products for Drug Discovery in the Genomics Era,” Nature Reviews Drug Discovery 14 (2015): 111–129, 10.1038/nrd4510. 25614221

[3] B. Chopra and A. K. Dhingra , “Natural Products: A Lead for Drug Discovery and Development,” Phytotherapy Research 35 (2021): 4660–4702, 10.1002/ptr.7099. 33847440

[4] D. J. Newman and G. M. Cragg , “Natural Products as Sources of New Drugs Over the Nearly Four Decades From 01/1981 to 09/2019,” Journal of Natural Products 83 (2020): 770–803, 10.1021/acs.jnatprod.9b01285. 32162523

[5] N. Dixon , L. S. Wong , T. H. Geerlings , and J. Micklefield , “Cellular Targets of Natural Products,” Natural Product Reports 24 (2007): 1288–1310, 10.1039/b616808f. 18033580

[6] B. Hanley , Natural and Unnatural Product Chemistry: From Molecules to Systems, ed. B. Hanley , (Springer Nature Switzerland, 2025), 249–262, 10.1007/978-3-031-81698-7.

[7] K. Jerye , H. Lüken , A. Steffen , et al., “Activity‐Based Protein Profiling Identifies Protein Disulfide‐Isomerases as Target Proteins of the Volatile Salinilactones,” Advanced Science 11 (2024): e2309515, 10.1002/advs.202309515. PMC11095149 38430530

[8] S. K. Saggu , A. Nath , and S. Kumar , “Myxobacteria: Biology and Bioactive Secondary Metabolites,” Research in Microbiology 174 (2023): 104079, 10.1016/j.resmic.2023.104079. 37169232

[9] J. Herrmann , A. A. Fayad , and R. Müller , “Natural Products From Myxobacteria: Novel Metabolites and Bioactivities,” Natural Product Reports 34 (2017): 135–160, 10.1039/C6NP00106H. 27907217

[10] A. Gavriilidou , S. A. Kautsar , N. Zaburannyi , et al., “Compendium of Specialized Metabolite Biosynthetic Diversity Encoded in Bacterial Genomes,” Nature Microbiology 7 (2022): 726–735, 10.1038/s41564-022-01110-2. 35505244

[11] L. Huo , J. J. Hug , C. Fu , X. Bian , Y. Zhang , and R. Müller , “Heterologous Expression of Bacterial Natural Product Biosynthetic Pathways,” Natural Product Reports 36 (2019): 1412–1436, 10.1039/C8NP00091C. 30620035

[12] T. Wang , A. Popoff , M. Hunter , et al., “Atypical Condensation Domains Guide Discovery and Illuminate Biosynthesis of Myxoglucamides Featuring Vinyl‐Substituted, α‐Oxidized γ‐Amino Acids,” Angewandte Chemie International Edition (2026): e6995139, 10.1002/anie.6995139. PMC13638107 42565744

[13] T. Böttcher , M. Pitscheider , and S. A. Sieber , “Natural Products and Their Biological Targets: Proteomic and Metabolomic Labeling Strategies,” Angewandte Chemie International Edition 49 (2010): 2680–2698, 10.1002/anie.200905352. 20333627

[14] C. Drahl , B. F. Cravatt , and E. J. Sorensen , “Protein‐Reactive Natural Products,” Angewandte Chemie International Edition 44 (2005): 5788–5809, 10.1002/anie.200500900. 16149114

[15] H. Deng , Q. Lei , Y. Wu , Y. He , and W. Li , “Activity‐Based Protein Profiling: Recent Advances in Medicinal Chemistry,” European Journal of Medicinal Chemistry 191 (2020): 112151, 10.1016/j.ejmech.2020.112151. 32109778

[16] S. Ziegler , V. Pries , C. Hedberg , and H. Waldmann , “Target Identification for Small Bioactive Molecules: Finding the Needle in the Haystack,” Angewandte Chemie International Edition 52 (2013): 2744–2792, 10.1002/anie.201208749. 23418026

[17] H. C. Brown and P. K. Jadhav , “Asymmetric Carbon‐carbon Bond Formation via .Beta.‐allyldiisopinocampheylborane. Simple Synthesis of Secondary Homoallylic Alcohols With Excellent Enantiomeric Purities,” Journal of the American Chemical Society 105 (1983): 2092–2093, 10.1021/ja00345a085.

[18] J. A. Dale , D. L. Dull , and H. S. Mosher , “.alpha.‐Methoxy‐.alpha.‐trifluoromethylphenylacetic Acid, a Versatile Reagent for the Determination of Enantiomeric Composition of Alcohols and Amines,” Journal of Organic Chemistry 34 (1969): 2543–2549, 10.1021/jo01261a013.

[19] R. R. Schmidt and J. Michel , “Facile Synthesis of α‐ and β‐*O*‐Glycosyl Imidates; Preparation of Glycosides and Disaccharides,” Angewandte Chemie International Edition 19 (1980): 731–732, 10.1002/anie.198007311.

[20] G. Pattenden and S. J. Reynolds , “Synthesis of (±)‐thienamycin Based on a New Approach to β‐lactams via 4‐exo‐trig Cyclisation of Carbamoylcobalt Salophens,” Journal of the Chemical Society, Perkin Transactions 1 (1994): 379–385, 10.1039/P19940000379.

[21] Z. Zhang , Y.‐S. Ding , A. R. Studenov , M. R. Gerasimov , and R. A. Ferrieri , “Novel Synthesis of [1‐^11^C]γ‐Vinyl‐γ‐Aminobutyric Acid ([1–^11^C]GVG) for Pharmacokinetic Studies of Addiction Treatment,” Journal of Labelled Compounds & Radiopharmaceuticals 45 (2002): 199–211, 10.1002/jlcr.544.

[22] J.‐M. Grassot , G. Masson , and J. Zhu , “Synthesis of α‐Ketoamides by a Molecular‐Sieves‐Promoted Formal Oxidative Coupling of Aliphatic Aldehydes With Isocyanides,” Angewandte Chemie International Edition 47 (2008): 947–950, 10.1002/anie.200704840. 18092314

[23] S. E. Schaus , B. D. Brandes , J. F. Larrow , et al., “Highly Selective Hydrolytic Kinetic Resolution of Terminal Epoxides Catalyzed by Chiral (salen)Co^III^ Complexes. Practical Synthesis of Enantioenriched Terminal Epoxides and 1,2‐Diols,” Journal of the American Chemical Society 124 (2002): 1307–1315, 10.1021/ja016737l. 11841300

[24] V. V. Rostovtsev , L. G. Green , V. V. Fokin , and K. B. Sharpless , “A Stepwise Huisgen Cycloaddition Process: Copper(I)‐Catalyzed Regioselective “Ligation” of Azides and Terminal Alkynes,” Angewandte Chemie International Edition 41 (2002): 2596–2599, 10.1002/1521-3773(20020715)41:14<2596::AID-ANIE2596>3.0.CO;2-4. 12203546

[25] C. S. Hughes , S. Moggridge , T. Müller , P. H. Sorensen , G. B. Morin , and J. Krijgsveld , “Single‐Pot, Solid‐Phase‐Enhanced Sample Preparation for Proteomics Experiments,” Nature Protocols 14 (2019): 68–85, 10.1038/s41596-018-0082-x. 30464214

[26] T. M. Penning , P. Wangtrakuldee , and R. J. Auchus , “Structural and Functional Biology of Aldo‐Keto Reductase Steroid‐Transforming Enzymes,” Endocrine Reviews 40 (2019): 447–475, 10.1210/er.2018-00089. PMC6405412 30137266

[27] T. M. Penning , “AKR1C3 (type 5 17β‐hydroxysteroid dehydrogenase/prostaglandin F synthase): Roles in Malignancy and Endocrine Disorders,” Molecular and Cellular Endocrinology 489 (2019): 82–91, 10.1016/j.mce.2018.07.002. PMC6422768 30012349

[28] M. Li , L. Zhang , J. Yu , et al., “AKR1C3 in Carcinomas: From Multifaceted Roles to Therapeutic Strategies,” Frontiers in Pharmacology 15 (2024): 1378292, 10.3389/fphar.2024.1378292. PMC10957692 38523637

[29] Y. Liu , S. He , Y. Chen , et al., “Overview of AKR1C3: Inhibitor Achievements and Disease Insights,” Journal of Medicinal Chemistry 63 (2020): 11305–11329, 10.1021/acs.jmedchem.9b02138. 32463235

[30] T. M. Penning , “Aldo‐Keto Reductase (AKR)1C3 Inhibitors: A Patent Review,” Expert Opinion on Therapeutic Patents 27 (2017): 1329–1340, 10.1080/13543776.2017.1379503. PMC5724044 28895472

[31] A. V. Carmona , S. Jonnalagadda , A. M. Case , et al., “Discovery of an Aldo‐Keto Reductase 1C3(AKR1C3) Degrader,” Communications Chemistry 7 (2024): 95, 10.1038/s42004-024-01177-4. PMC11059152 38684887

[32] A. Hara , S. Endo , T. Matsunaga , M. Soda , K. Yashiro , and O. El‐Kabbani , “Long‐Chain Fatty Acids Inhibit Human Members of the Aldo‐Keto Reductase 1C Subfamily,” Journal of Biochemistry 162 (2017): 371–379, 10.1093/jb/mvx041. 28992312

[33] S. Niebling , O. Burastero , J. Bürgi , et al., “FoldAffinity: Binding Affinities From nDSF Experiments,” Scientific Reports 11 (2021): 9572, 10.1038/s41598-021-88985-z. PMC8099913 33953265

[34] For calculation of K_d_ values from thermal shift assay the FoldAffinity WebTool was used: https://spc.embl‐hamburg.de/app/foldAffinity.

[35] M. C. Byrns , Y. Jin , and T. M. Penning , “Inhibitors of Type 5 17β‐hydroxysteroid Dehydrogenase (AKR1C3): Overview and Structural Insights,” The Journal of Steroid Biochemistry and Molecular Biology 125 (2011): 95–104, 10.1016/j.jsbmb.2010.11.004. PMC3047600 21087665

[36] O. El‐Kabbani , U. Dhagat , M. Soda , S. Endo , T. Matsunaga , and A. Hara , “Probing the Inhibitor Selectivity Pocket of Human 20α‐hydroxysteroid Dehydrogenase (AKR1C1) With X‐ray Crystallography and Site‐Directed Mutagenesis,” Bioorganic & medicinal chemistry letters 21 (2011): 2564–2567, 10.1016/j.bmcl.2011.01.076. 21414777

[37] B. Zhang , X.‐J. Hu , X.‐Q. Wang , et al., “Human 3α‐hydroxysteroid Dehydrogenase Type 3: Structural Clues of 5α‐DHT Reverse Binding and Enzyme Down‐Regulation Decreasing MCF7 Cell Growth,” Biochemical Journal 473 (2016): 1037–1046, 10.1042/BCJ20160083. 26929402

[38] M. He , X. Zhou , and X. Wang , “Glycosylation: Mechanisms, Biological Functions and Clinical Implications,” Signal Transduction and Targeted Therapy 9 (2024): 194, 10.1038/s41392-024-01886-1. PMC11298558 39098853

[39] M. Robello , E. Barresi , E. Baglini , S. Salerno , S. Taliani , and F. D. Settimo , “The Alpha Keto Amide Moiety as a Privileged Motif in Medicinal Chemistry: Current Insights and Emerging Opportunities,” Journal of Medicinal Chemistry 64 (2021): 3508–3545, 10.1021/acs.jmedchem.0c01808. PMC8154582 33764065

[40] G. R. Fulmer , A. J. M. Miller , N. H. Sherden , et al., “NMR Chemical Shifts of Trace Impurities: Common Laboratory Solvents, Organics, and Gases in Deuterated Solvents Relevant to the Organometallic Chemist,” Organometallics 29 (2010): 2176–2179, 10.1021/om100106e.

[41] Y. Perez‐Riverol , J. Bai , C. Bandla , et al., “The PRIDE Database Resources in 2022: A Hub for Mass Spectrometry‐Based Proteomics Evidence,” Nucleic Acids Research 50 (2022): D543–D552, 10.1093/nar/gkab1038. PMC8728295 34723319

[42] C. Du , J. Fang , J. Chen , et al., “Construction of the Tetracyclic Core of Calyciphylline B‐Type Daphniphyllum Alkaloids,” Organic Letters 21 (2019): 8718–8721, 10.1021/acs.orglett.9b03322. 31613108

[43] A. O. Adeniji , B. M. Twenter , M. C. Byrns , et al., “Development of Potent and Selective Inhibitors of Aldo–Keto Reductase 1C3 (Type 5 17β‐Hydroxysteroid Dehydrogenase) Based on N ‐Phenyl‐Aminobenzoates and Their Structure–Activity Relationships,” Journal of Medicinal Chemistry 55 (2012): 2311–2323, 10.1021/jm201547v. PMC3298089 22263837

[44] C. Vonrhein , C. Flensburg , P. Keller , et al., “Data Processing and Analysis With the autoPROC Toolbox,” Acta Crystallographica Section D: Biological Crystallography 67 (2011): 293–302, 10.1107/S0907444911007773. PMC3069744 21460447

[45] C. Vonrhein , C. Flensburg , and P. Keller , autoProc, Program for Diffraction Image Processing (Global Phasing Limited, 2022).

[46] W. Kabsch , “XDS,” Acta Crystallographica Section D: Biological Crystallography 66 (2010): 125–132, 10.1107/S0907444909047337. PMC2815665 20124692

[47] W. Kabsch , *XDS, Program for Diffraction Image Indexing*, Max Planck Institute for Medical Research, Heidelberg, Germany, **2023**.

[48] P. Evans , “Scaling and Assessment of Data Quality,” Acta Crystallographica Section D: Biological Crystallography 62 (2006): 72–82, 10.1107/S0907444905036693. 16369096

[49] P. Evans , *POINTLESS, Program for Space Group Determination*, Laboratory of Molecular Biology, Cambridge, U.K., **2021**.

[50] P. R. Evans and G. N. Murshudov , “How Good Are My Data and What Is the Resolution?” Acta Crystallographica Section D: Biological Crystallography 69 (2013): 1204–1214, 10.1107/S0907444913000061. PMC3689523 23793146

[51] P. Evans , *AIMLESS. Program for Scaling & Analysis of Unmerged Intensities*, Laboratory of Molecular Biology, Cambridge, U.K., **2022**.

[52] D. Liebschner , P. V. Afonine , M. L. Baker , et al., “Macromolecular Structure Determination Using X‐rays, Neutrons and Electrons: Recent Developments in Phenix,” Acta Crystallographica Section D: Biological Crystallography 75 (2019): 861–877, 10.1107/S2059798319011471. PMC6778852 31588918

[53] P. D. Adams , P. V. Afonine , and G. Bunkoczi , Phenix: Python‐based Hierarchical Environment for Integrated Xtallography, Program for Crystal Structure Refinement (Lawrence Berkeley Laboratory, 2022).

[54] A. J. McCoy , R. W. Grosse‐Kunstleve , P. D. Adams , M. D. Winn , L. C. Storoni , and R. J. Read , “Phaser Crystallographic Software,” Journal of Applied Crystallography 40 (2007): 658–674, 10.1107/S0021889807021206. PMC2483472 19461840

[55] *PHENIX: Phaser, Program for Molecular Replacement*, Lawrence Berkeley Laboratory, Berkeley, CA, USA, **2022**.

[56] P. Emsley , B. Lohkamp , W. G. Scott , and K. Cowtan , “Features and Development of Coot,” Acta Crystallographica Section D: Biological Crystallography 66 (2010): 486–501, 10.1107/S0907444910007493. PMC2852313 20383002

[57] P. Emsley , K. Cowtan , and B. Lohkamp , Coot. Marina Bay, (University of Oxford; University of York, 2012)..

