# Supplementary material for: Synthesis and Activity‐Based Protein Profiling Identifies Aldo‐Keto Reductase 1C3 as Target Protein of Myxoglucamides
Source: Angew Chem Int Ed Engl. 2026 Aug 10;65(41):e6489568. doi: 10.1002/anie.6489568 (PMC13638102; doi:10.1002/anie.6489568)
Supplement: Supplementary file 1 [file ANIE-65-e6489568-s001.docx]

Synthesis and Activity Based Protein Profiling Identifies Aldo-Keto Reductase 1C3 as Target Protein of Myxoglucamides

Thomas Siemon,^[a]^ Vivek K. Mishra,^[a]^ Mingming Zhao,^[b]^ Konrad Büssow,^[b]^ Alexander Popoff,^[c]^ Chengzhang Fu,^[c]^ Rolf Müller,^[c]^ Lothar Jänsch,^[d]^ Wulf Blankenfeldt,^[b]^ and Mark Brönstrup*^[a, e, f]^

[a]	Dr. T. Siemon, Dr. V. K. Mishra, Prof. Dr. M. Brönstrup
Department of Chemical Biology
Helmholtz-Centre for Infection Research (HZI)
Inhoffenstr. 7, 38124 Braunschweig, Germany
E-mail: [mark.broenstrup@helmholtz-hzi.de](mailto:mark.broenstrup@helmholtz-hzi.de)

[b]	M. Sc. Mingming Zhao, Dr. K. Büssow, Prof. Dr. W. Blankenfeldt
Department of Structure and Function of Proteins
Helmholtz-Centre for Infection Research (HZI)
Inhoffenstr. 7, 38124 Braunschweig, Germany

[c]	Dr. A. Popoff, Dr. C. Fu, Prof. Dr. R. Müller
Helmholtz-Institute for Pharmaceutical Research Saarland (HIPS)
Helmholtz Centre for Infection Research (HZI) and Department of Pharmacy (HZI)
Saarland University, Campus E8.1, 66123 Saarbrücken, Germany

[d]	Prof. Dr. L. Jänsch
Research Group Cellular Proteome Research
Helmholtz-Centre for Infection Research (HZI)
Inhoffenstr. 7, 38124 Braunschweig, Germany

[e]	Prof. Dr. M. Brönstrup
Institute of Organic Chemistry and Centre of Biomolecular Drug Research (BMWZ)
Leibniz Universität Hannover
Schneiderberg 1B, 30167 Hannover, Germany

[f]	Prof. Dr. M. Brönstrup
German Center for Infection Research
Site Hannover-Braunschweig
Inhoffenstraße 7, 38124 Braunschweig, Germany

**Table of Content**

[1	General Information	3](#_Toc225756690)

[2	Chemical Synthesis	4](#_Toc225756691)

[2.1	Synthesis of Myxoglucamide (1)	4](#_Toc225756692)

[2.2	Synthesis of Activity-Based Probe 16	14](#_Toc225756693)

[2.3	Synthesis of Myxoglucamide Analogs	18](#_Toc225756694)

[3	Activity-based Protein Profiling	30](#_Toc225756695)

[3.1	ABPP in A549	30](#_Toc225756696)

[3.2	Competitive ABPP	35](#_Toc225756697)

[4	Enzyme Inhibition Assays	36](#_Toc225756698)

[4.1	Aldehyde Dehydrogenase (ALDH) Inhibition Assay	36](#_Toc225756699)

[4.2	Aldo-Keto-Reductase 1 B1/B10 Inhibition Assay	37](#_Toc225756700)

[4.3	Aldo-Keto-Reductase 1 C1/C2/C3 Inhibition Assay	38](#_Toc225756701)

[5	AKR1C3 Expression and Purification	39](#_Toc225756702)

[6	Structure-Activity Relationship AKR1C3	39](#_Toc225756703)

[7	Cytotoxicity	41](#_Toc225756704)

[8	Michaelis-Menten Enzyme Kinetics AKR1C3	43](#_Toc225756705)

[9	Thermal Shift Assay (nanoDSF)	45](#_Toc225756706)

[9.1	Measurement of Denaturation Temperature (T_M_)	45](#_Toc225756707)

[9.2	Binding Affinity Determination	45](#_Toc225756708)

[10	X-Ray Crystal Structure of AKR1C3 with NADP^+^ and 1	49](#_Toc225756709)

[10.1	Co-Crystallization	49](#_Toc225756710)

[10.2	Data Collection, Refinement and Validation	49](#_Toc225756711)

[11	References	52](#_Toc225756712)

[12	NMR Spectra	53](#_Toc225756713)

# 1 General Information

**Column Chromatography**

For reaction control and determination of a suitable eluent mixture, thin layer chromatography (TLC) plates with silica 60 coated on aluminum with the fluorescent indicator F254 were used (Merck, 1.05554.0001). Staining of the TLC plates was carried out with a potassium permanganate or phosphomolybdic acid solutions. Columns were packed with silica gel (pore size 60 Å, particle size 230- 400 mesh, particle size 40-63 µm, Merck, 717185). Solvents were used from the supplier without further purification having the following purities: Baker Analyzed, ACS Grade, Baker Ultra Resi-Analyzed.

**Dry Solvents**

For water sensitive reactions, dry solvents were used from Thermo Scientific with an AcroSeal® cap.

**Starting Materials**

Starting material were purchased from commercial suppliers (Sigma-Aldrich, TCI, BLDpharm, Thermo Scientific, Acros Organics) and used without further purification.

**Liquid Chromatography–Mass Spectrometry (LCMS)**

For reaction control and purity assessment LCMS spectra were recorded on an Agilent 1260 Infinity II HPLC system equipped with an Agilent 6130 quadrupole mass detector with an electrospray ionisation (ESI) using ChemStation C.01.07 SR2 software. LC system: column: Gemini^®^ 3 µm NX-C18 110 Å, 50 × 2 mm (Phenomenex); solvent A: 0.1% formic acid in H2O; solvent B: 0.06% formic acid in MeCN; gradient: 5–95%B over 3 min with a constant flow rate of 1.5 mL min^-1^, detector: diode array detector (DAD) at 220, 254, 270 and 360 nm.

**High-Performance Liquid Chromatography (HPLC)**

Preparative HPLC was carried out with a Dionex UltiMate 3000 system from Thermo Scientific. Separation was achieved using the following column: Luna^®^ 5 µm C18(2) 100 Å, LC Column 250 x 21.2 mm, AXIA™ Packed (phenomenex, 00G-4252-P0-AX). As an eluent water (ultra pure water obtained from a PURELAB^®^ flex 1 system from ELGA) and acetonitrile (Ultra Gradient HPLC Grade, Baker HPLC Analyzed) with 0.1% formic acid additives each was used.

Analytical HPLC was performed on a Prominence 20 HPLC system (Shimadzu). Column: Gemini^®^ 3 µm NX-C18 110 Å, 50 × 2 mm (Phenomenex); solvent A: 0.1% formic acid in H2O; solvent B: 0.1% formic acid in MeCN; gradient: 1–100%B over 30 min with a constant flow rate of 1 mL min^-1^. The compounds were monitored by UV absorbance at 250 nm.

**Nuclear Magnetic Resonance (NMR) Spectroscopy**

NMR spectra were recorded at ambient temperature on Bruker Avance III (500/126 MHz, PABBO probe) or Bruker Avance III HD (700/176 MHz, CPTCI probe) spectrometers. Chemical shifts (δ) are reported in ppm and are referenced to the residual non-deuterated solvent peak.^[40]^ Coupling constants (J) are reported in Hz. ^1^H-NMR data are reported as: chemical shift, multiplicity (br, broad; s, single; d, doublet; t, triplet; q, quartet; quint, quintet; hept, heptet, m, multiplet; or a combination thereof), coupling constants, and number of nuclei. ^13^C-NMR Data are reported as: chemical shift, multiplicity (if not a singlet), coupling constants, and number of nuclei (if not one). Instead of ^13^C-NMR spectra, DEPT-Q spectra were measured for some compounds as stated in the chemical shift list. Samples were dissolved in deuterated solvents from Sigma-Aldrich. NMR spectra were processed with MestReNova 14.1.1 (Mestrelab Research).

**High Resolution Mass Spectrometry (HRMS)**

HRMS spectra were recorded on a Dionex Ultimate 3000 HPLC system equipped with a DAD and a Bruker maXis HD QTOF mass detector using ESI technique. Samples were directly injected via an Ultimate 3000RS autosampler (Thermo Fisher Scientific).

**Optical Rotation**

Optical rotation was measured in a quartz cuvette with a MCP 150 polarimeter from Anton Paar.

**Dimethyl Sulfoxide (DMSO)**

For all biological experiments as well as for the stock solutions, DMSO for cell culture was used (PanReac AppliChem, A3672).

**Proteomics Data Availability**

The mass spectrometry proteomics data have been deposited to the ProteomeXchange Consortium (http://proteomecentral.proteomexchange.org) via the PRIDE partner repository.^[41]^

The dataset identifier is PXD069317.

Username: reviewer_pxd069317@ebi.ac.uk

Password: meYXbcGUi6Ih

# 2 Chemical Synthesis

## 2.1 Synthesis of Myxoglucamide (1)

To a well stirred solution of (−)-β-methoxydiisopinocampheylborane (5.26 g, 16.6 mmol, 1.2 eq.) in Et_2_O (100 ml) at -80 °C was added allylmagnesium bromide (1M in Et_2_O, 16.6 mL, 16.6 mmol, 1.2 eq.)  slowly and drop-wise. After the complete addition of Grignard reagent the reaction was stirred −80 °C for 10 min, then the cooling bath was removed and the reaction was allowed to reach room temperature in 1 h. The suspension was cooled to −100 °C (EtOH + dry ice and liquid nitrogen) and a solution of isobutyraldehyde (1.27 mL, 13.9 mmol, 1.0 eq.) in Et_2_O (10 mL) was added within an hour via syringe pump. After complete addition, the reaction was allowed to stir at −100 °C to −90 °C for an hour. Thereafter the cooling bath was removed and the reaction was allowed to reach room temperature in an hour and further allowed stir vigorously for one more hour. Then the reaction was cooled to 0 °C and treated sequentially with a mixture of 1 M NaOH (60 mL) and 35% H_2_O_2_ (15 mL), and saturated aqueous NaHCO_3_ (50 mL). The mixture was stirred over night at room temperature and then the layers were separated, the aqueous layer was washed with Et_2_O (3 × 50 ml). The layers were combine and washed with brine (20 mL), dried over Na_2_SO_4_, concentrated *in vacuo*. The crude product was purified by flash column chromatography (silica gel, 5-10% Et_2_O in pentane) to obtain allyl alcohol **6** (1.03 g, 65%) as a colorless oil.

[α]_D_^20^ (CHCl_3_, c = 0.46) = ‒18.2

^1^H NMR (500 MHz, CDCl_3_) δ 5.83 (dddd, *J* = 16.8, 10.3, 8.1, 6.3 Hz, 1H), 5.16 – 5.09 (m, 2H), 3.38 (ddd, *J* = 8.9, 5.6, 3.5 Hz, 1H), 2.31 (dddt, *J* = 14.0, 6.4, 3.5, 1.4 Hz, 1H), 2.11 (dddt, *J* = 14.0, 9.0, 8.0, 1.1 Hz, 1H), 1.73 – 1.64 (m, 2H), 0.93 (d, *J* = 6.6 Hz, 3H), 0.91 (d, *J* = 6.6 Hz, 3H).

^13^C NMR (DEPT-Q, 126 MHz, CDCl_3_) δ = 135.4, 117.9, 75.4, 38.8, 33.1, 18.7, 17.5.

HRMS (ESI): calc. for C_14_H_29_O_2_ [2M+H]^+^: m/z = 229.2163, found: 229.2162.

**Confirmation of stereoselectivity by Mosher ester analysis^[18]^**

To a solution of alcohol **6** (7.0 mg, 61 μmol, 1.0 eq.) in THF (0.5 mL) were added (*S*)-(−)-α-methoxy-α-(trifluoromethyl)phenylacetic acid (21.5 mg, 92 μmol, 2.0 eq.), dicyclohexyl carbodiimide (19.0 mg, 92 μmol, 1.5 eq.) and 4-(dimethylamino)pyridine (1.5 mg, 12 μmol, 0.2 eq.). The reaction mixture was stirred at room temperature for 2 h and subsequently concentrated *in vacuo*. Purification by preparative TLC (silica gel, 10% EtOAc in cyclohexane) afforded the (*S*)-mosher ester **(*S*)-S1**.

^1^H NMR (500 MHz, CDCl_3_) δ 7.57 – 7.53 (m, 2H), 7.42 – 7.36 (m, 3H), 5.64 (ddt, *J* = 17.1, 10.2, 7.0 Hz, 1H), 5.04 – 4.97 (m, 3H), 3.53 (s, 3H), 2.35 (dd, *J* = 7.3, 6.0 Hz, 2H), 1.96 (heptd, *J* = 6.9, 4.9 Hz, 1H), 0.94 (d, *J* = 6.5 Hz, 3H), 0.93 (d, *J* = 6.3 Hz, 3H).

HRMS (ESI): calc. for C_17_H_21_F_3_O_3_Na [M+Na]^+^: m/z = 353.1335, found: 353.1334.

(*R*)-Mosher ester **(*R*)-S1** was synthesized by the same protocol as the **(*S*)-S1**.

^1^H NMR (500 MHz, CDCl_3_) δ 7.60 – 7.52 (m, 2H), 7.39 (td, *J* = 5.1, 2.1 Hz, 3H), 5.75 (ddt, *J* = 17.2, 10.2, 6.9 Hz, 1H), 5.13 – 4.97 (m, 3H), 3.56 (s, 3H), 2.41 (t, *J* = 6.7 Hz, 2H), 1.92 (heptd, *J* = 6.9, 5.9, 3.8 Hz, 1H), 0.86 (d, *J* = 6.8 Hz, 3H), 0.85 (d, *J* = 6.9 Hz, 3H).

HRMS (ESI): calc. for C_17_H_21_F_3_O_3_Na [M+Na]^+^: m/z = 353.1335, found: 353.1335.

| ***H*** | **(*S*)-S1** | **(*R*)-S1** | **Δ_SR_** |
| --- | --- | --- | --- |
| **1** | 0.94 | 0.86 | +0.08 |
| **2** | 0.93 | 0.85 | +0.08 |
| **3** | 1.96 | 1.92 | +0.04 |
| **5** | 2.35 | 2.41 | ‒0.06 |
| **6** | 5.64 | 5.75 | ‒0.11 |
| **7** | 5.01 | 5.08 | ‒0.07 |

Mosher ester analysis confirmed an (*S*)-configuration of the stereocenter with an e.r. of 82:18.^[18]^

To a solution of allylic alcohol **6** (1.03 g, 9.04 mmol, 1.0 eq.) in CH_2_Cl_2_ (20 mL) were added *tert*-butyl(chloro)diphenylsilane (2.98 g, 10.8 mmol, 1.2 eq.) and imidazole (1.54 g, 22.6 mmol, 2.5 eq.). After stirring at room temperature for 2 h, the reaction mixture was quenched by the addition of sat. NH_4_Cl (20 mL) and the aqueous phase was extracted with CH_2_Cl_2_ (3 x 20 mL). The combined organic phases were dried over Na_2_SO_4_, filtered and concentrated *in vacuo*. Purification by column chromatography (silica gel, pentane) afforded silylated alcohol **S2** (2.94 g, 92%) as a colorless oil.

^1^H NMR (700 MHz, CDCl_3_) δ 7.72 – 7.69 (m, 4H), 7.45 – 7.41 (m, 2H), 7.39 – 7.36 (m, 4H), 5.62 (ddt, *J* = 17.3, 10.3, 7.2 Hz, 1H), 4.91 – 4.84 (m, 2H), 3.60 (ddd, *J* = 6.9, 5.5, 3.6 Hz, 1H), 2.23 – 2.10 (m, 2H), 1.73 (heptd, *J* = 6.9, 3.7 Hz, 1H), 1.07 (s, 9H), 0.91 (d, *J* = 6.9 Hz, 3H), 0.86 (d, *J* = 6.8 Hz, 3H).

^13^C NMR (176 MHz, CDCl_3_) δ = 136.1, 135.6, 134.8, 134.4, 129.5, 129.4, 127.4, 127.3, 116.3, 77.6, 38.4, 32.1, 27.1, 19.6, 18.8, 16.8.

HRMS (ESI): calc. for C_23_H_33_OSi [M+H]^+^: m/z = 353.2295, found: 353.2297.

To a magnetically stirred suspension of sodium borohydride (1.89 g, 50.0 mmol, 6.0 eq.) in THF (100 mL) was added boron trifluoride diethyl etherate (6.50 g, 45.8 mmol, 5.5 eq.) at 0 °C. After stirring the reaction mixture at room temperature for 30 min, a solution of alkene **S2** (2.94 g, 8.33 mmol, 1.0 eq.) in THF (50 mL) was added dropwise at 0 °C. The mixture was allowed to reach room temperature within 1.5 h. Afterwards, 1 M NaOH was added dropwise at 0 °C until the strong gas evolution stopped. 1 M NaOH (50 mL) and 30% H_2_O_2_ (50 mL) were added sequentially to the reaction mixture at 0 °C. The mixture was stirred at room temperature overnight, before it was extracted with Et_2_O (3 x 50 mL). The combined organic phases were dried over anhydrous Na_2_SO_4_, filtered, and concentrated *in vacuo*. Purification by column chromatography (silica gel, 10% EtOAc in cyclohexane) afforded alcohol **S3** (2.45 g, 79%) as a colorless oil.

^1^H NMR (500 MHz, CDCl_3_) δ 7.71 – 7.67 (m, 4H), 7.44 – 7.35 (m, 6H), 3.58 – 3.53 (m, 1H), 3.38 – 3.33 (m, 2H), 1.76 (heptd, *J* = 6.7, 3.4 Hz, 1H), 1.43 – 1.31 (m, 4H), 1.06 (s, 9H), 0.91 (d, *J* = 6.8 Hz, 3H), 0.83 (d, *J* = 6.8 Hz, 3H).

^13^C NMR (126 MHz, CDCl_3_) δ = 136.0, 136.0, 134.8, 134.3, 129.5, 129.4, 127.4, 127.4, 77.7, 63.0, 32.5, 29.0, 28.7, 27.1, 27.1, 27.1, 19.6, 18.1, 17.5.

HRMS (ESI): calc. for C_23_H_35_O_2_Si [M+H]^+^: m/z = 371.2401, found: 371.2400.

To a solution of alcohol **S3** (2.45 g, 6.61 mmol, 1.0 eq.) in CH_2_Cl_2_ (50 mL) was added Dess-Martin periodinane (3.36 g, 7.93 mmol, 1.2 eq.). The suspension was stirred for 2 h at room temperature, filtered, and quenched by the addition of *i*-PrOH (5 mL). The mixture was concentrated *in vacuo* and purified by column chromatography (silica gel, 10% EtOAc in cyclohexane) to afford aldehyde **7** (2.14 g, 88%) as a colorless oil.

^1^H NMR (500 MHz, CDCl_3_) δ 9.46 (t, *J* = 1.6 Hz, 1H), 7.70 – 7.65 (m, 4H), 7.46 – 7.35 (m, 6H), 3.55 (dt, *J* = 6.6, 4.5 Hz, 1H), 2.33 (dddd, *J* = 17.5, 9.0, 6.2, 1.6 Hz, 1H), 2.21 (dddd, *J* = 17.4, 9.1, 6.4, 1.6 Hz, 1H), 1.77 (heptd, *J* = 6.9, 4.2 Hz, 1H), 1.70 – 1.63 (m, 2H), 1.06 (s, 9H), 0.91 (d, *J* = 6.8 Hz, 3H), 0.82 (d, *J* = 6.9 Hz, 3H).

^13^C NMR (126 MHz, CDCl_3_) δ = 202.4, 136.0, 135.9, 134.4, 134.0, 129.6, 129.6, 127.5, 127.5, 40.3, 32.9, 27.1, 27.1, 24.6, 19.6, 18.0, 17.4.

To a solution of benzyl 9-bromononanoate (4.78 g, 14.6 mmol, 1.0 eq.) in MeCN (100 mL) was added triphenylphosphine (4.21 g, 16.1 mmol, 1.1 eq.) and the mixture was refluxed for 4 h. The mixture was concentrated *in vacuo* and thoroughly dried to afford crude Wittig salt **8** that was used in the next step without further purification.

^1^H NMR (500 MHz, CDCl_3_) δ 7.85 – 7.75 (m, 9H), 7.71 – 7.66 (m, 6H), 7.35 – 7.26 (m, 5H), 5.07 (s, 2H), 3.80 – 3.69 (m, 2H), 2.28 (t, *J* = 7.5 Hz, 2H), 1.66 – 1.50 (m, 6H), 1.29 – 1.15 (m, 6H).

^13^C NMR (DEPT-Q, 126 MHz, CDCl_3_) δ = 173.6, 136.1, 135.0, 135.0, 133.7, 133.7, 130.6, 130.5, 128.5, 128.2, 128.1, 118.7, 118.1, 66.0, 34.2, 30.3, 30.2, 28.9, 28.9, 28.7, 24.8, 22.9, 22.6, 22.6, 22.5.

HRMS (ESI): calc. for C_34_H_38_O_2_P [M]^+^: m/z = 509.2604, found: 509.2609.

To a suspension of Wittig salt **8** (3.51 g, 5.95 mmol, 1.7 eq.) in THF (50 mL) was added LiHMDS (1 M in THF, 5.60 mL, 5.60 mmol, 1.6 eq.) dropwise at 0 °C. The mixture was stirred at 0 °C for 30 min upon which time a dark red suspension had formed. A solution of aldehyde **7** (1.29 g, 3.50 mmol, 1.0 eq.) in THF (10 mL) was added dropwise at 0 °C. The reaction mixture was stirred at 0 °C for 3 h, quenched by the addition of sat. NH_4_Cl and extracted with EtOAc (3 x 50 mL). The combined organic phases were dried over Na_2_SO_4_, filtered and concentrated *in vacuo*. Purification by column chromatiography (silica gel, 5-15% EtOAc in cyclohexane) afforded alkene **S4** (1.48 g, 71%) as a colorless oil.

^1^H NMR (500 MHz, CDCl_3_) δ 7.70 – 7.66 (m, 4H), 7.45 – 7.29 (m, 11H), 5.25 – 5.17 (m, 1H), 5.12 (s, 2H), 5.11 – 5.04 (m, 1H), 3.53 (td, *J* = 6.0, 3.7 Hz, 1H), 2.35 (t, *J* = 7.5 Hz, 2H), 1.98 – 1.89 (m, 1H), 1.87 – 1.81 (m, 2H), 1.79 – 1.68 (m, 2H), 1.67 – 1.60 (m, 2H), 1.44 – 1.36 (m, 2H), 1.35 – 1.15 (m, 8H), 1.06 (s, 9H), 0.90 (d, *J* = 6.9 Hz, 3H), 0.82 (d, *J* = 6.9 Hz, 3H).

^13^C NMR (DEPT-Q, 126 MHz, CDCl_3_) δ = 173.7, 136.1, 136.0, 134.9, 134.5, 130.2, 129.9, 129.7, 129.5, 129.4, 129.3, 128.6, 128.2, 127.4, 127.3, 77.8, 66.1, 34.3, 33.1, 32.4, 29.6, 29.2, 29.1, 29.1, 27.2, 25.0, 23.7, 19.6, 18.3, 17.3.

HRMS (ESI): calc. for C_39_H_55_O_3_Si [M+H]^+^: m/z = 599.3915, found: 599.3910.

A solution of silyl ether **S4** (760 mg, 1.27 mmol, 1.0 eq.) in THF (20 mL) was treated with tetrabutylammonium fluoride (1 M in THF, 5.1 mL, 5.1 mmol, 4.0 eq.) and the mixture was stirred overnight at room temperature. The reaction was quenched by the addition of sat. NH_4_Cl (20 mL) and extracted with EtOAc (3 x 20 mL). The combined organic phases were dried over Na_2_SO_4_, filtered and concentrated *in vacuo*. Purification by column chromatiography (silica gel, 20% EtOAc in cyclohexane) afforded alcohol **9** (312 mg, 68%) as a colorless oil.

^1^H NMR (500 MHz, CDCl_3_) δ 7.42 – 7.30 (m, 5H), 5.45 – 5.34 (m, 2H), 5.11 (s, 2H), 3.38 (ddd, *J* = 8.8, 5.1, 3.4 Hz, 1H), 2.35 (t, *J* = 7.5 Hz, 2H), 2.25 – 2.07 (m, 2H), 2.07 – 2.00 (m, 2H), 1.70 – 1.59 (m, 3H), 1.56 – 1.39 (m, 3H), 1.37 – 1.25 (m, 8H), 0.92 (d, *J* = 6.8 Hz, 3H), 0.91 (d, *J* = 6.7 Hz, 3H).

^13^C NMR (DEPT-Q, 126 MHz, CDCl_3_) δ = 173.7, 136.1, 130.5, 129.4, 128.5, 128.2, 127.7, 76.4, 66.1, 34.3, 34.1, 33.5, 29.6, 29.1, 29.0, 27.2, 26.5, 24.9, 23.9, 18.8, 17.1.

HRMS (ESI): calc. for C_23_H_37_O_3_ [M+H]^+^: m/z = 361.2737, found: 361.2736.

Alcohol **9** (312 mg, 0.87 mmol, 1.0 eq.) and tetraacetyl-d-glucosyl trichloroacetimidate **10** (852 mg, 1.73 mmol, 2.0 eq.) were dried under high vacuum in two separate flasks. Alcohol **9** was dissolved in CH_2_Cl_2_ (5 mL) and added to the other flask, followed by the addition of 4 Å molecular sieves (500 mg). The mixture was cooled to 0 °C and trimethylsilyl triflate (0.1 M in CH_2_Cl_2_, 0.87 mL, 0.087 mmol, 0.1 eq.) was added dropwise. After stirring at 0 °C for another 10 min, the reaction mixture was heated to reflux for 5 min upon which full conversion was achieved. The reaction mixture was neutralized with Et_3_N (12 µL), concentrated *in vacuo*, and purified by column chromatography (silica gel, 5-20% EtOAc in cyclohexane) to afford glycosylate **S5** (180 mg, 30%) as a colorless oil.

^1^H NMR (500 MHz, CDCl_3_) δ 7.41 – 7.30 (m, 5H), 5.39 – 5.32 (m, 2H), 5.20 (t, *J* = 9.6 Hz, 1H), 5.12 (s, 2H), 5.08 – 4.96 (m, 2H), 4.52 (d, *J* = 8.0 Hz, 1H), 4.21 (dd, *J* = 12.1, 5.3 Hz, 1H), 4.13 (dd, *J* = 12.1, 2.6 Hz, 1H), 3.67 (ddt, *J* = 10.1, 5.0, 2.5 Hz, 1H), 3.37 (dt, *J* = 8.1, 4.4 Hz, 1H), 2.36 (t, *J* = 7.5 Hz, 2H), 2.07 (s, 3H), 2.03 (s, 3H), 2.03 (s, 3H), 2.01 (s, 3H), 2.21 – 1.94 (m, 4H), 1.85 – 1.74 (m, 1H), 1.70 – 1.60 (m, 2H), 1.60 – 1.52 (m, 1H), 1.52 – 1.41 (m, 1H), 1.36 – 1.24 (m, 8H), 0.87 (d, *J* = 6.9 Hz, 3H), 0.86 (d, *J* = 6.9 Hz, 3H).

^13^C NMR (DEPT-Q, 126 MHz, CDCl_3_) δ = 173.6, 170.6, 170.3, 169.4, 169.2, 136.1, 130.1, 129.4, 128.5, 128.1, 100.9, 85.5, 73.0, 71.7, 71.5, 68.8, 66.0, 62.3, 34.3, 32.6, 31.2, 31.0, 29.6, 29.2, 29.1, 29.1, 27.2, 24.9, 23.1, 20.7, 20.6, 20.6, 17.8, 17.3.

HRMS (ESI): calc. for C_37_H_55_O_12_ [M+H]^+^: m/z = 691.3688, found: 691.3687.

To a solution of benzyl ester **S5** (180 mg, 0.261 mmol, 1.0 eq.) in EtOAc (5 mL) was added palladium on activated charcoal (10% Pd basis, 55 mg, 0.052 mmol, 0.2 eq.). The atmosphere was exchange against hydrogen by three-time evacuation and purging with hydrogen. The reaction mixture was stirred under a hydrogen atmosphere for 2 h at room temperature. The mixture was filtered through Celite and concentrated *in vacuo* to afford crude carboxylic acid **11** (157 mg, quant.) as a colorless oil. The crude product was used in the next step without further purification.

^1^H NMR (700 MHz, CDCl_3_) δ 5.18 (t, *J* = 9.5 Hz, 1H), 5.05 (t, *J* = 9.6 Hz, 1H), 4.98 (dd, *J* = 9.7, 7.9 Hz, 1H), 4.50 (d, *J* = 8.0 Hz, 1H), 4.20 (dd, *J* = 12.1, 4.9 Hz, 1H), 4.12 (d, *J* = 12.1 Hz, 1H), 3.68 – 3.62 (m, 1H), 3.32 (dt, *J* = 8.1, 4.2 Hz, 1H), 2.33 (t, *J* = 7.4 Hz, 2H), 2.06 (s, 3H), 2.01 (s, 3H), 2.01 (s, 3H), 1.99 (s, 3H), 1.81 – 1.74 (m, 1H), 1.61 (p, *J* = 7.5 Hz, 2H), 1.47 (tt, *J* = 8.9, 5.7 Hz, 1H), 1.43 – 1.17 (m, 17H), 0.84 (d, *J* = 7.4 Hz, 3H), 0.83 (d, *J* = 7.4 Hz, 3H).

^13^C NMR (DEPT-Q, 176 MHz, CDCl_3_) δ = 178.9, 170.6, 170.4, 169.4, 169.2, 100.9, 85.9, 73.0, 71.7, 71.4, 68.7, 62.3, 33.9, 31.0, 30.9, 29.6, 29.5, 29.5, 29.3, 29.2, 29.0, 25.3, 24.6, 20.7, 20.7, 20.6, 17.9, 17.3.

HRMS (ESI): calc. for C_30_H_51_O_12_ [M+H]^+^: m/z = 603.3375, found: 603.3374.

A suspension of methyl (*S*)-2-aminobutanoate hydrochloride (5.0 g, 32.5 mmol) in trimethyl orthoformate (20 mL) was stirred for 1 h at 105 °C, during which time it dissolved completely. The mixture was concentrated *in vacuo* to obtain formamide **S6** (4.7 g, quant.) as an off-white solid, which was used in the next step without further purification.

^1^H NMR (500 MHz, CDCl_3_) δ 8.22 (s, 1H), 6.28 (s, 1H), 4.67 (ddd, *J* = 7.9, 6.4, 5.3 Hz, 1H), 3.76 (s, 3H), 1.97 – 1.86 (m, 1H), 1.74 (dqd, *J* = 14.4, 7.5, 7.4 Hz, 1H), 0.91 (t, *J* = 7.5 Hz, 3H).*

^13^C NMR (126 MHz, CDCl_3_) δ 172.0, 160.6, 51.9, 51.4, 25.0, 8.9.*

*The NMRs show two rotamers with a ratio of 10:1. Only the NMR data of the major rotamer are displayed.

HRMS (ESI): calc. for C_6_H_11_NO_3_Na [M+Na]^+^: m/z = 168.0631, found: 168.0634.

To a solution of formamide **S6** (1.00 g, 6.90 mmol, 1.0 eq.) in CH_2_Cl_2_ (15 mL) was added Burgess reagent (1.64 g, 6.90 mmol, 1.0 eq.) and the mixture was stirred at 40 °C for 1 h. The reaction mixture was diluted with CH_2_Cl_2_ (50 mL) and washed with sat. citric acid (20 mL), H_2_O (20 mL) and brine (20 mL). The organic phase was dried over Na_2_SO_4_ and concentrated *in vacuo*. Purification by column chromatography (silica gel, 20% Et_2_O in pentane) afforded isocyanide **14** (520 mg, 59%) as a colorless oil. Analysis by chiral HPLC (Lux i-Cellulose-5, 20% *i*-PrOH in heptane, 1 mL/min, 210 nm) showed some slight epimerization (e.r. 88:12).

^1^H NMR (500 MHz, CDCl_3_) δ 4.25 (dd, *J* = 7.6, 4.9 Hz, 1H), 3.82 (s, 3H), 2.04 – 1.89 (m, 2H), 1.08 (t, *J* = 7.4 Hz, 3H).

^13^C NMR (126 MHz, CDCl_3_) δ 166.7, 159.4 (t, *J* = 4.7 Hz), 57.3 (t, *J* = 7.7 Hz), 52.8, 25.9, 9.1.

HRMS (ESI): calc. for C_6_H_10_NO_2_ [M+H]^+^: m/z = 128.0706, found: 128.0709.

To a solution of Boc-l-Homoserine lactone **12** (4.0 g, 19.9 mmol, 1.0 eq.) in THF (100 mL) was added DIBAL-H (1M in toluene, 23.8 mL, 23.8 mmol, 1.2 eq.) slowly via syringe pump over 45 min keeping the temperature below −70 °C (internal thermometer). After stirring at −70 °C for 1 h, the reaction was quenched by the addition of EtOAc (20 mL), followed by Rochelle salt (150 mL). The mixture was allowed to warm to room temperature and stirred for 1 h. The aq. phase was extracted with EtOAc (3 x 150 mL). The combined organic phases were washed with brine (100 mL), dried over Na_2_SO_4_ and concentrated *in vacuo*. Purification by column chromatography (silica gel, 40% EtOAc in cyclohexane) afforded lactol **S7** (3.94 g, 98%) as a white solid.

Isolated as a mixture of diastereomers (d.r. ~67:33).

HRMS (ESI): calc. for C_9_H_17_NO_4_Na [M+Na]^+^: m/z = 226.1050, found: 226.1052.

Major Diastereomer:

^1^H NMR (500 MHz, CDCl_3_) δ 5.34 – 5.25 (m, 1H), 5.08 (d, *J* = 8.9 Hz, 1H), 4.14 – 4.03 (m, 2H), 3.84 (dd, *J* = 8.1 Hz, 1H), 3.77 (s, 1H), 2.32 – 2.23 (m, 1H), 1.93 – 1.78 (m, 1H), 1.43 (s, 9H).

^13^C NMR (126 MHz, CDCl_3_) δ = 155.7, 95.0, 79.7, 65.9, 53.5, 29.2, 28.5.

Minor Diastereomer:

^1^H NMR (500 MHz, CDCl_3_) δ 5.34 – 5.25 (m, 1H), 4.70 (d, *J* = 8.8 Hz, 1H), 4.14 – 4.03 (m, 2H), 3.97 (dd, *J* = 7.9 Hz, 1H), 3.60 (s, 1H), 2.49 – 2.36 (m, 1H), 1.78 – 1.69 (m, 1H), 1.43 (s, 9H).

^13^C NMR (126 MHz, CDCl_3_) δ = 155.5, 101.8, 80.0, 66.2, 53.5, 29.2, 28.5.

To a suspension of MePPh_3_Br (6.73 g, 18.8 mmol, 1.9 eq.) in toluene and THF (4:1, 50 mL) was added KO*t*-Bu (1.78 g, 15.9 mmol, 1.6 eq.) at −10 °C. After stirring at −10 °C for 45 min, a solution of lactol **S7** (2.02 g, 9.91 mmol, 1.0 eq.) in THF (20 mL) was added. The mixture was stirred at −10 °C and then at room temperature for 1 h. The reaction was quenched by the addition of sat. NH_4_Cl (100 mL) and extracted with EtOAc (3 x 100 mL). The combined organic phases were dried over Na_2_SO_4_ and concentrated *in vacuo*. Purification by column chromatography (silica gel, 25% EtOAc in cyclohexane) afforded alkene **S8** (1.47 g, 74%) as a colorless oil.

^1^H NMR (700 MHz, CDCl_3_) δ 5.83 (ddd, *J* = 16.3, 10.2, 5.0 Hz, 1H), 5.20 (d, *J* = 17.4 Hz, 1H), 5.13 (d, *J* = 10.5 Hz, 1H), 4.65 (br. s, 1H), 4.41 – 4.32 (m, 1H), 3.71 – 3.63 (m, 2H), 2.68 (br. s, 1H), 1.95 – 1.88 (m, 1H), 1.45 (s, 9H).*

^13^C NMR (126 MHz, CDCl_3_) δ 156.1, 137.8, 114.3, 79.5, 58.2, 48.6, 37.5, 27.9.*

HRMS (ESI): calc. for C_10_H_19_NO_3_Na [M+Na]^+^: m/z = 224.1257, found: 224.1260.

*NMR data are in accordance with the literature.^[42]^

To a solution of alcohol **S8** (1.43 g, 7.14 mmol, 1.0 eq.) in CH_2_Cl_2_ (20 mL) was added Dess-Martin periodinane (4.54 g, 10.7 mmol, 1.5 eq.) and the mixture was stirred at room temperature for 2 h. The solids were filtered off and the filtrate was quenched by the addition of MeOH (5 mL) and subsequently concentrated *in vacuo*. The slurry was resuspended in CH_2_Cl_2_ and filtered again. Purification of the filtrate by column chromatography (silica gel, 20% EtOAc in cyclohexane) afforded aldehyde **13** (1.01 g, 71%) as a colorless oil that solidified over time.

^1^H NMR (500 MHz, CDCl_3_) δ 9.74 (s, 1H), 5.85 (ddd, *J* = 16.4, 10.4, 5.4 Hz, 1H), 5.19 (dd, *J* = 17.2, 1.6 Hz, 1H), 5.15 (dd, *J* = 10.5, 1.5 Hz, 1H), 4.87 (s, 1H), 4.61 (s, 1H), 2.76 – 2.64 (m, 2H), 1.42 (s, 9H).*

^13^C NMR (126 MHz, CDCl_3_) δ 200.0, 154.6, 136.6, 115.2, 79.4, 47.9, 47.7, 27.8.*

HRMS (ESI): calc. for C_10_H_17_NO_3_Na [M+Na]^+^: m/z = 222.1100, found: 222.1099.

*NMR data are in accordance with the literature.^[42]^

To a solution of aldehyde **13** (165 mg, 0.828 mmol, 1.0 eq.) in MeOH (0.8 mL) was added *N*‑methylhydroxylamine hydrochloride (76.0 mg, 0.911 mmol, 1.1 eq.), NaHCO_3_ (139 mg, 1.65 mmol, 2.0 eq.) and 4Å molecular sieves (600 mg). The mixture was stirred at room temperature for 30 min. Isonitrile **14** (e.r. 88:12, 116 mg, 0.911 mmol, 1.1 eq.) and acetic acid (426 µL, 7.45 mmol, 9.0 eq.) were added and the reaction mixture was stirred over night at room temperature. Analysis by TLC and LCMS indicated mainly formation α-acyloxyamino amide intermediate and only little amount of the desired ketoamide product. Therefore, more 4 Å molecular sieves (800 mg) and MeOH (0.5 mL) was added and the reaction mixture was stirred for another 4 h at room temperature. Subsequently, molecular sieves were filtered off and the filtrate was concentrated *in vacuo*. Purification by column chromatography (silica gel, 20-25% EtOAc in cyclohexane) afforded ketoamide **S9** (130 mg, 46%) as a colorless oil.

The product was isolated as a mixture of diastereomers (d.r. 84:16). Only the NMR data of the major diastereomer are displayed.

^1^H NMR (700 MHz, CDCl_3_) δ 7.36 (d, *J* = 8.5 Hz, 1H), 5.84 (ddd, *J* = 16.6, 10.3, 5.4 Hz, 1H), 5.21 (d, *J* = 17.3 Hz, 1H), 5.14 (d, *J* = 10.5 Hz, 1H), 4.96 – 4.90 (m, 1H), 4.66 (s, 1H), 4.53 (ddd, *J* = 8.6, 6.9, 5.3 Hz, 1H), 3.77 (s, 3H), 3.27 – 3.20 (m, 1H), 3.13 – 3.06 (m, 1H), 1.99 – 1.91 (m, 1H), 1.84 – 1.76 (m, 1H), 1.43 (s, 9H), 0.94 (t, *J* = 7.4 Hz, 3H).

^13^C NMR (126 MHz, CDCl_3_) δ 195.5, 171.2, 159.0, 154.6, 136.5, 115.1, 79.3, 52.8, 52.1, 48.6, 41.5, 27.8, 25.0, 9.0.

HRMS (ESI): calc. for C_16_H_26_N_2_O_6_Na [M+Na]^+^: m/z = 365.1683, found: 365.1693.

Ketoamide **S9** (34.0 mg, 0.100 mmol, 2.0 eq.) was treated with HCl in dioxane (4 M, 1.0 mL, 4.0 mmol) and stirred at room temperature for 1 h. The reaction mixture was concen­trated *in vacuo* to afford the amine hydrochloride **15** (27.8 mg, quant) as a colorless oil that was used in the next step without further purification. The amine hydro­chloride **15** was dissolved in CH_2_Cl_2_ (2 mL) and carboxylic acid **11** (30.0 mg, 0.050 mmol, 1.0 eq.) followed by HATU (28.4 mg, 0.075 mmol, 1.5 eq.), and DIPEA (26.1 µL, 0.149 mmol, 3.0 eq.) were added. The mixture was stirred overnight at room temperature and was then diluted with EtOAc (10 mL). The organic phase was washed with sat. NaHCO_3_ (10 mL), 1 M HCl (10 mL) and brine (10 mL), dried over Na_2_SO_4_ and concentrated *in vacuo*. Purification by column chromatography (silica gel, 20-25% EtOAc in cyclohexane) afforded amide **S10** (20.2 mg, 49%) as a colorless oil.

^1^H NMR (700 MHz, CDCl_3_) δ 7.34 (d, *J* = 8.3 Hz, 1H), 6.02 (d, *J* = 8.5 Hz, 1H), 5.82 (ddd, *J* = 17.2, 10.4, 5.4 Hz, 1H), 5.19 – 5.12 (m, 3H), 5.06 – 5.02 (m, 1H), 4.97 (dd, *J* = 9.7, 8.0 Hz, 1H), 4.95 – 4.87 (m, 1H), 4.52 – 4.47 (m, 2H), 4.19 (dd, *J* = 12.1, 5.2 Hz, 1H), 4.11 (dd, *J* = 12.1, 2.6 Hz, 1H), 3.75 (s, 3H), 3.65 (ddd, *J* = 10.0, 5.3, 2.7 Hz, 1H), 3.33 – 3.30 (m, 1H), 3.19 – 3.13 (m, 2H), 2.15 (dd, *J* = 8.5, 6.9 Hz, 2H), 2.09 – 2.05 (m, 1H), 2.06 (s, 3H), 2.01 (s, 3H), 2.01 (s, 3H), 1.98 (s, 3H), 1.96 – 1.90 (m, 1H), 1.82 – 1.73 (m, 2H), 1.61 – 1.55 (m, 2H), 1.50 – 1.19 (m, 17H), 0.92 (t, *J* = 7.5 Hz, 3H), 0.84 (d, *J* = 6.8 Hz, 3H), 0.83 (d, *J* = 6.8 Hz, 3H).

^13^C NMR (176 MHz, CDCl_3_) δ = 196.1, 172.5, 171.6, 170.6, 170.3, 169.4, 169.2, 159.6, 136.6, 115.8, 100.9, 86.0, 73.0, 71.7, 71.4, 68.7, 62.3, 53.4, 52.5, 47.5, 41.3, 36.7, 31.0, 31.0, 29.6, 29.6, 29.4, 29.3, 29.2, 25.6, 25.4, 25.3, 20.7, 20.7, 20.6, 20.6, 17.9, 17.3, 9.5.

HRMS (ESI): calc. for C_41_H_67_N_2_O_15_ [M+H]^+^: m/z = 827.4536, found: 827.4537.

To a solution of glycosylate **S10** (20.2 mg, 0.024 mmol) in THF/MeOH (3:1, 0.5 mL) was added 1 M LiOH_aq_ (250 μL, 0.25 mmol, 4.0 eq.) and the reaction mixture was stirred at room temperature for 1 h. The mixture was neutralized by the addition of with 1 M HCl (250 μL, 0.25 mmol), filtered through a syringe filter and purified by RP-HPLC (MeCN in H_2_O + 0.1% formic acid). Myxoglucamide (**1**, 12.5 mg, 80%, HPLC purity > 99%) was obtained as white solid.

| No | δ_H_ **1**^a^ | δ_H_ *Lit.* | Δ_H_ | δ_C_ **1**^a^ | δ_C_ *Lit.* | Δ_C_ |
| --- | --- | --- | --- | --- | --- | --- |
| 1 | 0.93 (d, 6.7) | 0.93 (d, 7.0) | 0 | 18.4 | 18.4 | 0 |
| 2 | 0.94 (d, 6.7) | 0.94 (d, 6.6) | 0 | 18.0 | 18.1 | -0.1 |
| 3 | 1.91 (m) | 1.90 (m) | +0.01 | 31.8 | 31.6 | +0.2 |
| 4 | 3.49 (m) | 3.49 (m) | 0 | 85.2 | 85.2 | 0 |
| 5 | 1.49 (m) | 1.49 (m) | 0 | 31.8 | 31.8 | 0 |
| 6 | 1.38-1.26 (m) | 1.36 (m) |  | 26.6 | 26.6 | 0 |
| 7-13 | 1.38-1.26 (m) | 1.32-1.27 (m) |  | 30.9-30.2 | 31.0-30.3 |  |
| 14 | 1.59 (m) | 1.58 (m) | +0.01 | 27.0 | 27.1 | -0.1 |
| 15 | 2.17 (t, 7.4) | 2.16 (t) | +0.01 | 37.0 | 37.1 | -0.1 |
| 16 |  |  |  | 175.6 | 175.7 | -0.1 |
| 17 | NH | | | NH | | |
| 18 | 4.88 (m) | 4.87 (m) | +0.01 | 48.7 | 48.6 | +0.1 |
| 19a | 2.99 (dd, 16.7, 8.5) | 2.99 (dd, 16.8, 8.4) | 0 | 42.7 | 42.7 | 0 |
| 19b | 3.21 (dd, 16.8, 5.5) | 3.21 (dd, 16.8, 5.3) | 0 |  |  |  |
| 20 |  |  |  | 196.2 | 196.3 | -0.1 |
| 21 |  |  |  | 162.2 | 162.0 | +0.2 |
| 22 | NH | | | NH | | |
| 23 | 4.30 (m) | 4.29 (m) | +0.01 | 55.0 | 55.5 | -0.5 |
| 24 |  |  |  | 174.5 | 175.2 | +0.3 |
| 25 | OH | | | OH | | |
| 26 | 5.86 (ddd, 17.3, 10.4, 5.5) | 5.86 (ddd, 17.3, 10.4, 5.5) | 0 | 138.4 | 138.6 | -0.2 |
| 27a | 5.19 (dt, 17.2, 1.5) | 5.19 (dt, 17.2, 1.4) | 0 | 115.5 | 115.5 | 0 |
| 27b | 5.11 (dt, 10.4, 1.4) | 5.11 (dt, 10.4, 1.4) | 0 |  |  |  |
| 28a | 1.81 (m) | 1.80 (m) | +0.01 | 25.7 | 25.9 | -0.2 |
| 28b | 1.96 (m) | 1.96 (m) | 0 |  |  |  |
| 29 | 0.96 (t, 7.4) | 0.95 (t, 7.4) | +0.01 | 10.4 | 10.5 | -0.1 |
| 1‘ | 4.30 (d, 7.9) | 4.30 (d, 7.8) | 0 | 103.9 | 103.4 | +0.5 |
| 2‘ | 3.24 – 3.15 (m) | 3.17 (dd) |  | 75.4 | 75.5 | -0.1 |
| 3‘ | 3.39 – 3.29 (m) | 3.32 (m) |  | 78.2 | 78.3 | -0.1 |
| 4‘ | 3.39 – 3.29 (m) | 3.31 |  | 71.8 | 71.7 | +0.1 |
| 5' | 3.24 – 3.15 (m) | 3.22 (m) |  | 77.6 | 77.7 | -0.1 |
| 6’a | 3.85 (dd, 11.7, 2.5) | 3.85 (dd, 11.6, 2.4) | 0 | 63.0 | 63.0 | 0 |
| 6‘b | 3.68 (dd, 11.7, 5.6) | 3.68 (dd, 11.5, 5.5) | 0 |  |  |  |

HRMS (ESI): calc. for C_32_H_57_N_2_O_11_ [M+H]^+^: m/z = 645.3957, found: 645.3954.

**Table S1.** Comparison of NMR spectra data of synthesized and isolated myxoglucamide.^[12]^

^a^ acquired at 500 MHz/ 126 MHz in CD_3_OD.

## 2.2 Synthesis of Activity-Based Probe 16

NiCl_2_ • 6 H_2_O (5.10 g, 21.4 mmol, 1.0 eq.) and Zinc dust (7.01 g, 107.2 mmol, 5.0 eq.) were suspended in dry pyridine (50 mL) and ethyl acrylate (10.5 mL, 96.5 mmol, 4.5 eq.) was added. The mixture was stirred at 60 °C for 30 min upon which a dark red solution formed. It was cooled to room temperature and 11-bromoundec-1-ene (5.00 g, 21.4 mmol, 1.0 eq.) dissolved in dry pyridine (10 mL) was added dropwise. The mixture was stirred at room temperature overnight and then diluted with EtOAc (100 mL) and filtered through Celite. The pad was washed with an excess of EtOAc. The combined org. phases were washed with 1 M HCl (3x) and brine, dried over Na_2_SO_4_ and concentrated *in vacuo*. Purification by column chromatography (silica gel, 0 – 4% EtOAc in cyclohexane) afforded alkene **17** (1.34 g, 24%) as a colorless oil.

^1^H NMR (700 MHz, CDCl_3_) δ 5.81 (ddd, *J* = 16.9, 10.2, 6.7 Hz, 1H), 4.99 (ddd, *J* = 17.1, 1.7, 1.7 Hz, 1H), 4.92 (ddd, *J* = 10.2, 2.4, 1.2 Hz, 1H), 4.12 (q, *J* = 7.1 Hz, 2H), 2.28 (t, *J* = 7.6 Hz, 2H), 2.06 – 2.01 (m, 2H), 1.63 – 1.59 (m, 2H), 1.39 – 1.34 (m, 2H), 1.33 – 1.23 (m, 14H), 1.25 (t, *J* = 7.1 Hz, 3H).

^13^C NMR (126 MHz, CDCl_3_) δ 174.0, 139.3, 114.1, 60.2, 34.4, 33.8, 29.6, 29.5, 29.5, 29.3, 29.2, 29.0, 25.0, 14.3.

HRMS (ESI): calc. for C_16_H_31_O_2_ [M+H]^+^: m/z = 255.2319, found: 255.2319.

To a solution of alkene **17** (1.34 g, 5.28 mmol, 1.0 eq.) in CH_2_Cl_2_ (20 mL) was added *meta*-chloroperbenzoic acid (≤ 77%, 1.30 g, 5.80 mmol, 1.1 eq.) at 0 °C. The reaction mixture was warmed to room temperature and stirred for 4 h. The reaction mixture was diluted with CH_2_Cl_2_ (50 mL) and quenched by the addition of sat. Na_2_S_2_O_3_ (20 mL). The organic phase was washed with sat. NaHCO_3_ (20 mL containing 1M NaOH (2 mL)) and brine (20 mL), dried over Na_2_SO_4_ and concentrated *in vacuo*. The crude product ***rac*-18** was used in the next step without further purification.

A solution of (*S*,*S*)-Co(salen) (91.0 mg, 0.15 mmol, 3 mol%) in toluene (2 mL) and AcOH (86.2 μL, 1.50 mmol, 0.3 eq.) was stirred at room temperature under air for 2 h until the dark red mixture turned dark brown. Toluene and excess AcOH were removed *in vacuo*. The racemic epoxide ***rac*-18** from the previous step (1.36 g, 5.03 mmol, 1.0 eq.) and THF (1.5 mL) were added, followed by the addition of H_2_O (50.0 μL, 2.77 mmol, 0.55 eq.) at 0 °C. The mixture was stirred at room temperature overnight then concentrated *in vacuo*. Purification by column chromatography (silica gel, 5% EtOAc in cyclohexane) afforded the enantiopure epoxide **18** (516 mg, 38%) as a slightly red oil.

^1^H NMR (500 MHz, CDCl_3_) δ 4.13 (q, *J* = 7.1 Hz, 2H), 2.91 (tdd, *J* = 5.6, 3.9, 2.7 Hz, 1H), 2.76 (dd, *J* = 5.0, 3.9 Hz, 1H), 2.47 (dd, *J* = 5.0, 2.7 Hz, 1H), 2.29 (t, *J* = 7.4 Hz, 2H), 1.66 – 1.40 (m, 6H), 1.36 – 1.24 (m, 14H), 1.26 (t, *J* = 7.1 Hz, 3H).

^13^C NMR (126 MHz, CDCl_3_) δ 173.5, 59.7, 52.0, 46.7, 33.9, 32.0, 29.1, 29.0, 29.0, 28.8, 28.7, 25.5, 24.5, 13.8.

HRMS (ESI): calc. for C_16_H_31_O_3_ [M+H]^+^: m/z = 271.2268, found: 271.2270.

To a solution of (triisopropylsilyl)acetylene (0.36 mL, 1.61 mmol, 1.3 eq.) in THF (10 mL) was added *n*‑butyllithium (1.6 M in hexane, 1.00 mL, 1.61 mmol, 1.3 eq.) dropwise at −78 °C. The mixture was stirred at −78 °C for 30 min, then BF_3_•OEt_2_ (0.20 mL, 1.61 mmol, 1.3 eq.) was added and after another 15 min a solution of epoxide **18** (334 mg, 1.24 mmol, 1.0 eq.) in THF (2.5 mL) was added. The reaction mixture was stirred at −78 °C for 2 h and subsequently quenched by the addition of sat NH_4_Cl (10 mL). The aqueous phase was extracted with EtOAc (3 x 15 ml). The combined organic phases were dried over Na_2_SO_4_ and concentrated *in vacuo*. Purification by column chromatography (silica gel, 4-10% EtOAc in cyclohexane) afforded alcohol **S11** (420 mg, 75%) as a colorless oil.

^1^H NMR (500 MHz, CDCl_3_) δ 4.13 (q, *J* = 7.1 Hz, 2H), 3.74 (p, *J* = 6.1 Hz, 1H), 2.51 (dd, *J* = 16.8, 4.9 Hz, 1H), 2.40 (dd, *J* = 16.8, 6.6 Hz, 1H), 2.29 (t, *J* = 7.6 Hz, 2H), 1.65 – 1.39 (m, 7H), 1.37 – 1.24 (m, 17H), 1.26 (t, *J* = 7.1 Hz, 3H), 1.10 – 1.05 (m, 18H).

^13^C NMR (126 MHz, CDCl_3_) δ 173.5, 104.3, 83.1, 69.5, 59.7, 35.7, 33.9, 29.1, 29.1, 29.0, 29.0, 28.8, 28.7, 28.4, 26.4, 25.1, 24.5, 18.2, 13.8, 10.7.

HRMS (ESI): calc. for C_27_H_53_O_3_Si [M+H]^+^: m/z = 453.3759, found: 453.3757.

**Confirmation of stereoselectivity by a Mosher ester^[18]^**

To a solution of alcohol **S11** (2.5 mg, 5.5 μmol, 1.0 eq.) in THF (0.5 mL) were added (*S*)-(−)-α-methoxy-α-(trifluoromethyl)phenylacetic acid (2.6 mg, 11 μmol, 2.0 eq.), DCC (2.3 mg, 11 μmol, 2.0 eq.) and DMAP (0.7 mg, 5.5 μmol, 1.0 eq.). The reaction mixture was stirred at room temperature for 2 h and subsequently concentrated *in vacuo*. Purification by column chromatography (silica gel, 20% EtOAc in cyclohexane) afforded the (*S*)-mosher ester **(*S*)-S12** as a colorless oil.

^1^H NMR (500 MHz, CDCl_3_) δ 7.57 – 7.53 (m, 2H), 7.42 – 7.35 (m, 3H), 5.16 (tdd, *J* = 7.0, 5.6, 5.6 Hz, 1H), 4.12 (q, *J* = 7.1 Hz, 2H), 3.60 (s, 3H), 2.65 (d, *J* = 5.9 Hz, 2H), 2.29 (t, *J* = 7.6 Hz, 2H), 1.77 – 1.69 (m, 2H), 1.68 – 1.44 (m, 7H), 1.41 – 1.13 (m, 14H), 1.25 (t, *J* = 7.1 Hz, 3H), 1.07 – 0.99 (m, 18H).

HRMS (ESI): calc. for C_37_H_60_F_3_O_5_Si [M+H]^+^: m/z = 669.4157, found: 669.4156.

The same protocol was used to synthesize the (*R*)-mosher ester **(*R*)-S12**.

^1^H NMR (500 MHz, CDCl_3_) δ 7.55 – 7.51 (m, 2H), 7.41 – 7.37 (m, 3H), 5.13 (tdd, *J* = 7.1, 6.8, 4.6 Hz, 1H), 4.12 (q, *J* = 7.1 Hz, 2H), 3.53 (s, 3H), 2.62 (dd, *J* = 17.0, 4.6 Hz, 1H), 2.55 (dd, *J* = 17.0, 6.8 Hz, 1H), 2.28 (t, *J* = 7.6 Hz, 2H), 1.91 – 1.75 (m, 2H), 1.65 – 1.54 (m, 7H), 1.36 – 1.21 (m, 14H), 1.25 (t, *J* = 7.1 Hz, 3H), 1.04 – 1.01 (m, 18H).

HRMS (ESI): calc. for C_37_H_60_F_3_O_5_Si [M+H]^+^: m/z = 669.4157, found: 669.4162.

| ***H*** | **(*S*)-S12** | **(*R*)-S12** | **Δ_SR_** |
| --- | --- | --- | --- |
| **1** | 2.65 | 2.62, 2.55 | +0,06 |
| **2** | 5.16 | 5.13 | +0.03 |
| **3** | 1.73 | 1.83 | -0.10 |

Mosher ester analysis confirms the desired (*S*)-configuration and an enantiomeric excess >99%.^[18]^

To a solution of ethyl ester **S11** (172 mg, 0.38 mmol, 1.0 eq.) in THF (6 mL) and MeOH (2 mL) was added LiOH (1 M in H_2_O, 2 mL, 2 mmol, 5.0 eq.). The mixture was stirred for 3 h at room temperature and subsequently acidified with 1 M HCl. The aqueous phase was extracted with EtOAc (3 x 15 mL). The combined organic phases were dried over Na_2_SO_4_ and concentrated *in vacuo*. The crude carboxylic acid **S13** (160 mg, quant) was used in the next step without further purification.

Crude carboxylic acid **S13** (80 mg, 0.19 mmol, 1.0 eq.) was dissolved in TBAF solution (1M in THF, 1.0 mL, 1.0 mmol, 5.0 eq.) and stirred at room temperature for 1 h. The reaction mixture was quenched by the addition of sat NH_4_Cl (5 mL) and 1 M HCl (1 mL), and extracted with EtOAc (3 x 10 mL). The combined organic phases were dried over Na_2_SO_4_ and concentrated *in vacuo*. Purification by column chromatography (silica gel, 30% EtOAc in cyclohexane) afforded carboxylic acid **19** (41.3 mg, 80%) as a colorless oil.

^1^H NMR (500 MHz, CDCl_3_) δ 3.75 (qd, *J* = 6.5, 4.9 Hz, 1H), 2.41 (ddd, *J* = 16.7, 4.8, 2.6 Hz, 1H), 2.32 (t, *J* = 7.5 Hz, 2H), 2.31 – 2.27 (m, 1H), 2.03 (t, *J* = 2.7 Hz, 1H), 1.65 – 1.57 (m, 2H), 1.56 – 1.49 (m, 2H), 1.45 – 1.19 (m, 17H).

^13^C NMR (126 MHz, CDCl_3_) δ 179.2, 80.4, 70.3, 69.5, 35.6, 33.6, 29.0, 28.9, 28.7, 28.5, 26.8, 25.0, 24.2.

HRMS (ESI): calc. for C_16_H_29_O_3_ [M+H]^+^: m/z = 269.2111, found: 269.2120.

To a solution of carboxylic acid **19** (41.3 mg, 0.15 mmol, 1.0 eq.) and amine hydro­chloride **15** (42.9 mg, 0.15 mmol, 1.0 eq.) in DMF (1.5 mL) were added HATU (70.2 mg, 0.19 mmol, 1.2 eq.), HOAt (25.1 mg, 0.19 mmol, 1.2 eq.) and DIPEA (67.2 µL, 0.39 mmol, 2.5 eq.). The mixture was stirred at room temperature for 4 h and was then diluted with EtOAc (25 mL). The organic phase was washed with sat. NaHCO_3_ (20 mL), 10% citric acid (20 mL) and brine (20 mL), dried over Na_2_SO_4_ and concentrated *in vacuo*. Purification by column chromatography (silica gel, 30-40% EtOAc in cyclohexane) afforded amide **S14** (43.3 mg, 57%) as a colorless oil.

^1^H NMR (500 MHz, CDCl_3_) δ 7.29 (d, *J* = 8.3 Hz, 1H), 5.94 (d, *J* = 8.5 Hz, 1H), 5.77 (ddd, *J* = 17.2, 10.4, 5.3 Hz, 1H), 5.12 (dd, *J* = 17.2, 1.6 Hz, 1H), 5.08 (dd, *J* = 10.2, 1.5 Hz, 1H), 4.91 – 4.84 (m, 1H), 4.44 (ddd, *J* = 8.3, 6.9, 5.4 Hz, 1H), 3.70 (s, 3H), 3.72 – 3.65 (m, 1H), 3.11 (d, *J* = 6.0 Hz, 2H), 2.36 (ddd, *J* = 16.7, 4.7, 2.6 Hz, 1H), 2.25 (ddd, *J* = 16.7, 6.8, 2.7 Hz, 1H), 2.10 (t, *J* = 7.6 Hz, 2H), 1.99 (t, *J* = 2.7 Hz, 1H), 1.94 – 1.79 (m, 2H), 1.78 – 1.68 (m, 2H), 1.58 – 1.44 (m, 4H), 1.41 – 1.32 (m, 1H), 1.29 – 1.15 (m, 14H), 0.86 (t, *J* = 7.5 Hz, 3H).

^13^C NMR (126 MHz, CDCl_3_) δ 196.2, 172.6, 171.6, 159.6, 136.6, 115.9, 81.0, 70.8, 69.9, 53.4, 52.6, 47.5, 41.3, 36.8, 36.2, 30.1, 29.7, 29.5, 29.5, 29.5, 29.4, 29.3, 29.2, 27.4, 25.6, 25.6, 25.4, 9.5.

HRMS (ESI): calc. for C_27_H_45_N_2_O_6_ [M+H]^+^: m/z = 493.3272, found: 493.3281.

Alcohol **S14** (22.6 mg, 0.024 mmol, 1.0 eq.) and tri­chloro­acet­imidate **10** (108 mg, 0.22 mmol, 2.5 eq.) were co-evaporated with toluene. 4 Å molecular sieves powder (220 mg) was added and the flask was evacuated at high vacuum for 30 min. Dry CH_2_Cl_2_ (0.5 mL) was added and mixture was stirred for 30 min at room temperature. TMSOTf (0.05 M in CH_2_Cl_2_, 0.35 mL, 0.018 mmol, 0.2 eq.) was added and the mixture was heated to 40 °C for 1.5 h. Only around 50% conversion was achieved but the reaction stalled. The mixture was filtered and concentrated *in vacuo*. Purification by column chromatography (silica gel, 35-50% EtOAc in cyclohexane) afforded 23 mg of an inseparable mixture of glucosyl **S15** and unidentified impurities.

^1^H NMR (500 MHz, CDCl_3_) δ 7.35 (d, *J* = 8.4 Hz, 1H), 6.01 (d, *J* = 8.7 Hz, 1H), 5.82 (ddd, *J* = 17.2, 10.5, 5.4 Hz, 1H), 5.71 (d, *J* = 5.2 Hz, 1H), 5.20 – 5.12 (m, 2H), 4.97 – 4.85 (m, 2H), 4.49 (ddd, *J* = 8.3, 6.9, 5.4 Hz, 1H), 4.41 (dd, *J* = 5.4, 3.0 Hz, 1H), 4.22 – 4.15 (m, 2H), 4.14 – 3.99 (m, 2H), 3.95 – 3.86 (m, 1H), 3.77 – 3.70 (m, 1H), 3.75 (s, 3H), 3.16 (d, *J* = 5.9 Hz, 2H), 2.43 – 2.37 (m, 2H), 2.10 (s, 3H), 2.08 (s, 3H), 2.07 (s, 3H), 2.03 (s, 3H), 2.23 – 1.89 (m, 3H), 1.84 – 1.48 (m, 7H), 1.34 – 1.20 (m, 14H), 0.92 (t, *J* = 7.5 Hz, 3H).

^13^C NMR (126 MHz, CDCl_3_) δ 196.1, 172.5, 171.6, 170.7, 169.6, 169.1, 159.6, 136.5, 115.8, 96.9, 80.8, 73.1, 72.1, 70.2, 70.0, 68.1, 67.0, 63.0, 53.4, 52.5, 47.5, 41.3, 36.7, 34.6, 29.6, 29.5, 29.5, 29.4, 29.3, 29.2, 25.6, 25.4, 25.1, 25.0, 21.7, 20.7, 20.7, 20.5, 9.4.

HRMS (ESI): calc. for C_41_H_63_N_2_O_15_ [M+H]^+^: m/z = 823.4223, found: 823.4237.

To a solution of glycosyl **S15** (23 mg, impure) in THF (0.5 mL) was added LiOH (1 M in H_2_O, 200 μL, 0.20 mmol) and the reaction mixture was stirred at room temperature for 1 h. The mixture was acidified with 1 M HCl, filtered through a syringe filter and purified by RP-HPLC (MeCN in H_2_O + 0.1% formic acid). carboxylic acid **S16** (2.5 mg, 16% over 2 steps) was obtained as a white solid.

^1^H NMR* (500 MHz, CD_3_OD) δ 5.86 (ddd, *J* = 17.2, 10.4, 5.5 Hz, 1H), 5.19 (dt, *J* = 17.2, 1.4 Hz, 1H), 5.11 (dt, *J* = 10.4, 1.4 Hz, 1H), 4.93 – 4.83 (m, 1H), 4.70 – 4.49 (m, 1H), 4.39 (d, *J* = 7.8 Hz, 1H), 4.31 (dd, *J* = 8.0, 5.1 Hz, 1H), 3.90 – 3.86 (m, 1H), 3.86 (dd, *J* = 11.8, 2.3 Hz, 1H), 3.68 (dd, *J* = 11.8, 5.4 Hz, 1H), 3.38 – 3.32 (m, 2H), 3.26 (ddd, *J* = 9.5, 5.4, 2.3 Hz, 1H), 3.21 (dd, *J* = 16.7, 5.4 Hz, 1H), 3.16 (dd, *J* = 9.0, 7.7 Hz, 1H), 2.99 (dd, *J* = 16.7, 8.4 Hz, 1H), 2.50 (ddd, *J* = 16.8, 4.7, 2.7 Hz, 1H), 2.39 (ddd, *J* = 16.8, 7.3, 2.7 Hz, 1H), 2.28 (t, *J* = 2.7 Hz, 1H), 2.17 (t, *J* = 6.9 Hz, 2H), 2.12 – 1.88 (m, 1H), 1.86 – 1.53 (m, 6H), 1.38 – 1.27 (m, 15H), 0.95 (t, *J* = 7.4 Hz, 3H).

^13^C NMR* (DEPT-Q, 126 MHz, CD_3_OD) δ 196.5, 175.8, 162.2, 138.6, 115.7, 102.8, 78.2, 78.0, 78.0, 75.3, 71.9, 63.0, 49.4, 48.9, 42.8, 37.2, 35.5, 30.9, 30.9, 30.8, 30.8, 30.6, 30.4, 27.2, 26.2, 26.1, 24.6, 10.5.

* Partial hemiacetal formation at the ketoamide in CD_3_OD was observed. Only the NMR signals of the product are displayed.

HRMS (ESI): calc. for C_32_H_53_N_2_O_11_ [M+H]^+^: m/z = 641.3644, found: 641.3653.

Alkyne **S16** (2.5 mg, 3.9 μmol, 1.0 eq.), Biotin-PEG3-azide (3.5 mg, 7.8 μmol, 2.0 eq.), CuSO_4_ •5 H_2_O (2.0 mg, 7.8 μmol, 2.0 eq.), sodium ascorbate (3.9 mg, 20 μmol, 5.0 eq.) and THPTA (3.4 mg, 7.8 μmol, 2.0 eq.) were dissolved in DMSO/H_2_O (0.5 mL) and the reaction mixture was stirred at room temperature for 2 h. The mixture was filtered through a syringe filter and purified by RP-HPLC (MeCN in H_2_O + 0.1% formic acid). The activity-based probe **16** (2.1 mg, 50%) was obtained as a white solid.

^1^H NMR (600 MHz, DMSO-*d*_6_) δ 8.53 (d, *J* = 8.0 Hz, 1H), 7.97 (s, 1H), 7.86 (d, *J* = 8.1 Hz, 1H), 7.81 (t, *J* = 5.8 Hz, 1H), 6.41 (s, 1H), 6.35 (s, 1H), 5.79 (ddd, *J* = 16.2, 10.5, 5.5 Hz, 1H), 5.08 (d, *J* = 16.5 Hz, 1H), 5.02 (d, *J* = 10.3 Hz, 1H), 4.97 – 4.83 (m, 1H), 4.74 – 4.68 (m, 1H), 4.46 (t, *J* = 5.4 Hz, 2H), 4.35 (s, 1H), 4.30 (dd, *J* = 7.7, 5.1 Hz, 1H), 4.27 (d, *J* = 7.9 Hz, 1H), 4.12 (td, *J* = 8.6, 5.0 Hz, 2H), 3.87 – 3.82 (m, 1H), 3.79 (t, *J* = 5.3 Hz, 2H), 3.66 (d, *J* = 11.2 Hz, 1H), 3.52 – 3.49 (m, 2H), 3.48 – 3.46 (m, 6H), 3.46 – 3.40 (m, 1H), 3.39 – 3.36 (m, 3H), 3.20 – 3.02 (m, 7H), 2.96 (t, *J* = 8.4 Hz, 1H), 2.93 – 2.86 (m, 2H), 2.81 (dd, *J* = 12.5, 5.3 Hz, 1H), 2.80 – 2.75 (m, 1H), 2.59 – 2.51 (m, 2H), 2.09 – 1.96 (m, 4H), 1.86 – 1.79 (m, 1H), 1.77 – 1.69 (m, 1H), 1.60 (ddt, *J* = 12.4, 9.6, 6.0 Hz, 1H), 1.55 – 1.41 (m, 5H), 1.36 – 1.15 (m, 21H), 0.85 (t, *J* = 7.6 Hz, 3H).

^13^C NMR (DEPT-Q, 151 MHz, DMSO-*d*_6_) δ 196.0, 172.4, 172.1, 171.4, 162.7, 142.9, 138.3, 123.8, 114.3, 101.3, 76.8, 76.8, 76.7, 73.7, 70.1, 69.7, 69.6, 69.6, 69.5, 69.2, 68.7, 61.2, 61.0, 59.2, 55.4, 53.7, 49.3, 49.2, 46.5, 41.6, 38.4, 35.3, 35.1, 33.6, 29.1, 29.1, 29.0, 28.8, 28.6, 28.2, 28.0, 25.3, 25.2, 24.7, 23.9, 10.1.

HRMS (ESI): calc. for C_50_H_85_N_8_O_16_S [M+H]^+^: m/z = 1085.5799, found: 1085.5812.

## 2.3 Synthesis of Myxoglucamide Analogs

To a solution of benzyl ester **S4** (57.5 mg, 0.096 mmol, 1.0 eq.) in EtOAc (5 mL) was added palladium on activated charcoal (10% Pd basis, 19 mg, 0.019 mmol, 0.2 eq.). The atmosphere was exchange against hydrogen by three-time evacuation and purging with hydrogen. The reaction mixture was stirred under a hydrogen atmosphere for 2 h at room temperature. The mixture was filtered through Celite and concentrated *in vacuo* to afford crude carboxylic acid **S17** (49.1 mg, quant.) as a colorless oil. The crude product was used in the next step without further purification.

To a solution of crude carboxylic acid **S17** (49.1 mg, 0.096 mmol, 1.0 eq.) and amine hydrochloride **15** (53.6 mg, 0.192 mmol, 2.0 eq.) in DMF (0.8 mL) were added HATU (54.8 mg, 0.144 mmol, 1.5 eq.), HOAt (19.6 mg, 0.144 mmol, 1.5 eq.) and DIPEA (42.0 µL, 0.240 mmol, 2.5 eq.). The mixture was stirred overnight at room temperature and was then diluted with EtOAc (25 mL). The organic phase was washed with sat. NaHCO_3_ (20 mL), 10% citric acid (20 mL) and brine (20 mL), dried over Na_2_SO_4_ and concentrated *in vacuo*. Purification by column chromatography (silica gel, 20-25% EtOAc in cyclohexane) afforded amide **S18** (58 mg, 82%) as a colorless oil.

^1^H NMR (500 MHz, CDCl_3_) δ 7.70 – 7.67 (m, 4H), 7.44 – 7.33 (m, 6H), 6.01 (d, *J* = 8.6 Hz, 1H), 5.84 (ddd, *J* = 17.2, 10.4, 5.3 Hz, 1H), 5.19 (d, *J* = 17.1 Hz, 1H), 5.15 (d, *J* = 10.5 Hz, 1H), 4.98 – 4.90 (m, 1H), 4.52 (ddd, *J* = 8.3, 6.9, 5.4 Hz, 1H), 3.77 (s, 3H), 3.51 (td, *J* = 5.9, 3.7 Hz, 1H), 3.19 (d, *J* = 5.9 Hz, 2H), 2.20 – 2.14 (m, 2H), 2.00 – 1.91 (m, 1H), 1.85 – 1.69 (m, 2H), 1.65 – 1.55 (m, 2H), 1.37 – 0.96 (m, 28H), 0.94 (t, *J* = 7.5 Hz, 3H), 0.90 (d, *J* = 6.8 Hz, 3H), 0.82 (d, *J* = 6.8 Hz, 3H).

^13^C NMR (126 MHz, CDCl_3_) δ = 195.7, 172.1, 171.1, 159.1, 136.1, 135.6, 135.6, 134.6, 134.1, 128.9, 128.8, 126.9, 126.8, 115.4, 77.6, 52.9, 52.1, 47.0, 40.8, 36.3, 32.6, 31.9, 29.1, 29.1, 29.1, 29.0, 29.0, 29.0, 28.9, 28.9, 28.8, 26.7, 25.2, 25.1, 25.0, 19.1, 17.9, 16.7, 9.0.

HRMS (ESI): calc. for C_43_H_67_N_2_O_6_Si [M+H]^+^: m/z = 735.4763, found: 735.4765.

To a solution of silyl ether **S18** (29 mg, 0.039 mmol, 1.0 eq.) in MeCN (0.5 mL) was added HF • pyridine (70% HF, 56 μL, 1.95 mmol, 50 eq.) and the mixture was stirred for 3 h at room temperature. Due to incomplete conversion more HF • pyridine (56 μL, 1.95 mmol, 50 eq.) was added and the mixture was stirred overnight at room temperature. The reaction was quenched by the addition of sat. NaHCO_3_ (10 mL) and extracted with EtOAc (3 x 10 mL). The combined organic phases were dried over Na_2_SO_4_ and concentrated *in vacuo*. Purification by column chromatography (silica gel, 1% MeOH in CHCl_3_) afforded alcohol **33** (15 mg, 77%) as a colorless oil.

^1^H NMR (500 MHz, CDCl_3_) δ 7.35 (d, *J* = 8.5 Hz, 1H), 5.98 (d, *J* = 8.4 Hz, 1H), 5.83 (ddd, *J* = 17.2, 10.4, 5.3 Hz, 1H), 5.18 (ddd, *J* = 17.2, 1.8, 0.8 Hz, 1H), 5.15 (ddd, *J* = 10.5, 1.7, 0.8 Hz, 1H), 4.97 – 4.89 (m, 1H), 4.51 (ddd, *J* = 8.3, 6.9, 5.4 Hz, 1H), 3.77 (s, 3H), 3.37 – 3.33 (m, 1H), 3.24 – 3.12 (m, 2H), 2.18 – 2.14 (m, 2H), 2.00 – 1.90 (m, 1H), 1.84 – 1.75 (m, 1H), 1.71 – 1.56 (m, 5H), 1.49 – 1.44 (m, 2H), 1.38 – 1.21 (m, 15H), 0.93 (t, *J* = 7.5 Hz, 3H), 0.91 (d, *J* = 6.5 Hz, 3H), 0.90 (d, *J* = 6.5 Hz, 3H).

^13^C NMR (176 MHz, CDCl_3_) δ = 196.2, 172.5, 171.6, 159.6, 136.6, 115.9, 76.7, 53.4, 52.5, 47.5, 33.4, 18.9, 17.1, 9.5.

HRMS (ESI): calc. for C_27_H_49_N_2_O_6_ [M+H]^+^: m/z = 497.3585, found: 497.3584.

To a solution of methyl ester **33** (8.0 mg, 0.016 mmol, 1.0 eq.) in THF (0.5 mL) was added LiOH (1 M in H_2_O, 200 μL, 0.20 mmol, 11 eq.) and the reaction mixture was stirred at room temperature for 1 h. The mixture was acidified with 1M HCl, filtered through a syringe filter and purified by RP-HPLC (MeCN in H_2_O + 0.1% formic acid). Carboxylic acid **20** (3.0 mg, 39%) was obtained as a white solid.

^1^H NMR (600 MHz, DMSO-*d*_6_) δ 8.38 (d, *J* = 7.6 Hz, 1H), 7.86 (d, *J* = 8.1 Hz, 1H), 5.79 (ddd, *J* = 17.3, 10.4, 5.3 Hz, 1H), 5.07 (ddd, *J* = 17.2, 1.7, 1.7 Hz, 1H), 5.02 (ddd, *J* = 10.4, 1.5, 1.5 Hz, 1H), 4.75 – 4.65 (m, 1H), 4.06 – 3.97 (m, 1H), 3.14 – 3.09 (m, 2H), 2.90 (dd, *J* = 16.8, 8.1 Hz, 1H), 2.03 (td, *J* = 7.3, 2.8 Hz, 2H), 1.85 – 1.77 (m, 1H), 1.74 – 1.67 (m, 1H), 1.54 – 1.30 (m, 5H), 1.26 – 1.18 (m, 19H), 0.81 (d, *J* = 6.8 Hz, 3H), 0.81 (t, *J* = 7.4 Hz, 3H), 0.80 (d, *J* = 6.8 Hz, 3H).

^13^C NMR (151 MHz, DMSO-*d*_6_) δ 199.2, 175.5, 174.6, 163.4, 141.4, 117.4, 77.5, 56.9, 49.7, 44.8, 38.5, 36.8, 36.3, 32.4, 32.3, 32.2, 32.1, 31.9, 31.7, 28.8, 28.4, 27.0, 22.1, 20.6, 13.2.

HRMS (ESI): calc. for C_26_H_47_N_2_O_6_ [M+H]^+^: m/z = 483.3429, found: 483.3427.

**General Procedure A for the synthesis of analogs 21 − 32**

To a solution of the respective fatty acid (1.0 eq.) and amine hydrochloride **15** (1.5 eq.) in CH_2_Cl_2_, were added HATU (1.5 eq.), and DIPEA (3.0 eq.). The mixture was stirred overnight at room temperature and was then diluted with EtOAc. The organic phase was washed with 1 M HCl, sat. NaHCO_3_, and brine, dried over Na_2_SO_4_ and concentrated *in vacuo*. Without further purification, the crude amide was dissolved in a mixture of THF/MeOH (3:1) and 1 M LiOH_aq_ (10 eq.) was added. The mixture was stirred until full conversion was achieved, neutralized with 1 M HCl and purified by RP-HPLC (C18, MeOH in H_2_O + 1% formic acid). The final products were obtained as white powders after lyophilisation.

 ^1^H NMR* (700 MHz, MeOD) δ 5.84 (ddd, *J* = 16.5, 10.5, 5.5 Hz, 1H), 5.19 (d, *J* = 17.1 Hz, 1H), 5.09 (d, *J* = 10.4 Hz, 1H), 4.91 – 4.85 (m, 1H), 4.38 – 4.29 (m, 1H), 3.17 (dd, *J* = 16.5, 5.4 Hz, 1H), 2.93 (dd, *J* = 16.5, 8.5 Hz, 1H), 2.01 – 1.88 (m, 1H), 1.87 – 1.74 (m, 1H), 1.43 (s, 9H), 0.96 (t, *J* = 7.4 Hz, 3H).

^13^C NMR* (DEPT-Q, 176 MHz, MeOD) δ = 196.6, 162.3, 157.9, 157.8, 139.2, 115.3, 80.3, 55.4, 50.4, 43.4, 28.9, 26.0, 10.5.

* Partial hemiacetal formation at the ketoamide in CD_3_OD observed. Only the NMR signals of the product are displayed.

HRMS (ESI): calc. for C_15_H_25_N_2_O_6_ [M+H]^+^: m/z = 329.1707, found: 329.1707.

^1^H NMR (600 MHz, DMSO-d6) δ 8.51 (d, *J* = 8.0 Hz, 1H), 7.85 (d, *J* = 8.2 Hz, 1H), 5.78 (ddd, *J* = 17.3, 10.4, 5.3 Hz, 1H), 5.07 (dt, *J* = 17.2, 1.6 Hz, 1H), 5.02 (dt, *J* = 10.4, 1.5 Hz, 1H), 4.73 – 4.67 (m, 1H), 4.11 (ddd, *J* = 8.8, 7.9, 4.9 Hz, 1H), 3.10 (dd, *J* = 16.9, 5.9 Hz, 1H), 2.90 (dd, *J* = 16.8, 8.1 Hz, 1H), 2.02 (td, *J* = 7.3, 2.8 Hz, 2H), 1.86 – 1.77 (m, 1H), 1.76 – 1.67 (m, 1H), 1.45 (p, *J* = 7.5 Hz, 2H), 1.28 – 1.14 (m, 4H), 0.84 (t, *J* = 7.2 Hz, 6H).

^13^C NMR (DEPT-Q, 151 MHz, DMSO-d6) δ = 195.8, 172.5, 171.4, 160.8, 138.3, 114.3, 53.4, 46.5, 41.8, 35.3, 30.8, 24.9, 23.7, 21.8, 13.8, 10.3.

HRMS (ESI): calc. for C_16_H_27_N_2_O_5_ [M+H]^+^: m/z = 327.1915, found: 327.1914.

^1^H NMR (700 MHz, DMSO-d6) δ 12.77 (s, 1H), 8.51 (d, *J* = 8.0 Hz, 1H), 7.85 (d, *J* = 8.2 Hz, 1H), 5.78 (ddd, *J* = 17.3, 10.4, 5.3 Hz, 1H), 5.07 (dt, *J* = 17.2, 1.6 Hz, 1H), 5.02 (dt, *J* = 10.4, 1.5 Hz, 1H), 4.73 – 4.66 (m, 1H), 4.11 (ddd, *J* = 8.9, 7.9, 4.9 Hz, 1H), 3.11 (dd, *J* = 16.8, 5.9 Hz, 1H), 2.90 (dd, *J* = 16.8, 8.1 Hz, 1H), 2.06 – 1.97 (m, 2H), 1.86 – 1.78 (m, 1H), 1.76 – 1.67 (m, 1H), 1.48 – 1.41 (m, 2H), 1.29 – 1.16 (m, 12H), 0.84 (t, *J* = 7.1 Hz, 3H), 0.84 (t, *J* = 7.4 Hz, 3H).

^13^C NMR (DEPT-Q, 176 MHz, DMSO-d6) δ = 195.8, 172.5, 171.4, 160.8, 138.2, 114.3, 53.4, 46.5, 41.8, 35.3, 31.3, 28.9, 28.8, 28.7, 28.6, 25.2, 23.7, 22.1, 13.9, 10.3.

HRMS (ESI): calc. for C_20_H_35_N_2_O_5_ [M+H]^+^: m/z = 383.2541, found: 383.2540.

^1^H NMR (700 MHz, DMSO-d6) δ 12.76 (s, 1H), 8.51 (d, *J* = 8.0 Hz, 1H), 7.85 (d, *J* = 8.2 Hz, 1H), 5.78 (ddd, *J* = 17.2, 10.4, 5.3 Hz, 1H), 5.07 (dt, *J* = 17.3, 1.6 Hz, 1H), 5.01 (dt, *J* = 10.4, 1.5 Hz, 1H), 4.74 – 4.65 (m, 1H), 4.11 (tt, *J* = 8.4, 4.2 Hz, 1H), 3.11 (dd, *J* = 16.8, 5.9 Hz, 1H), 2.90 (dd, *J* = 16.8, 8.0 Hz, 1H), 2.09 – 1.97 (m, 2H), 1.86 – 1.77 (m, 1H), 1.75 – 1.67 (m, 1H), 1.45 (qt, *J* = 8.5, 4.0 Hz, 2H), 1.30 – 1.14 (m, 21H), 0.84 (t, *J* = 7.1 Hz, 3H), 0.84 (t, *J* = 7.4 Hz, 3H).

^13^C NMR (DEPT-Q, 176 MHz, DMSO-d6) δ = 195.8, 172.5, 171.4, 160.8, 138.2, 114.3, 53.4, 46.5, 41.8, 35.3, 31.3, 29.0, 29.0, 29.0, 28.9, 28.7, 28.7, 28.6, 25.2, 23.7, 22.1, 13.9, 10.3.

HRMS (ESI): calc. for C_24_H_43_N_2_O_5_ [M+H]^+^: m/z = 439.3167, found: 439.3165.

^1^H NMR* (700 MHz, MeOD) δ 5.86 (ddd, *J* = 17.2, 10.4, 5.6 Hz, 1H), 5.19 (dt, *J* = 17.2, 1.4 Hz, 1H), 5.11 (dt, *J* = 10.4, 1.4 Hz, 1H), 4.90 – 4.85 (m, 1H), 4.33 (dd, *J* = 8.1, 5.2 Hz, 1H), 3.20 (dd, *J* = 16.7, 5.4 Hz, 1H), 2.99 (dd, *J* = 16.7, 8.4 Hz, 1H), 2.18 – 2.14 (m, 2H), 2.02 – 1.92 (m, 1H), 1.85 – 1.77 (m, 1H), 1.63 – 1.55 (m, 2H), 1.29 (s, 30H), 0.96 (t, *J* = 7.4 Hz, 3H), 0.90 (t, *J* = 7.1 Hz, 3H).

^13^C NMR* (DEPT-Q, 176 MHz, MeOD) δ = 196.4, 175.8, 174.6, 162.4, 138.6, 115.7, 55.2, 42.9, 37.2, 33.2, 30.9, 30.9, 30.9, 30.9, 30.8, 30.6, 30.6, 30.4, 27.2, 25.9, 23.9, 14.6, 10.6.

* Partial hemiacetal formation at the ketoamide in CD_3_OD observed. Only the NMR signals of the product are displayed.

HRMS (ESI): calc. for C_28_H_50_N_2_O_5_ [M+H]^+^: m/z = 495.3793, found: 495.3792.

^1^H NMR* (500 MHz, MeOD) δ 5.86 (ddd, *J* = 17.3, 10.4, 5.5 Hz, 1H), 5.36 – 5.32 (m, 2H), 5.19 (dt, *J* = 17.2, 1.5 Hz, 1H), 5.11 (dt, *J* = 10.3, 1.4 Hz, 1H), 4.91 – 4.86 (m, 1H), kk4.33 (dd, *J* = 8.0, 5.1 Hz, 1H), 3.21 (dd, *J* = 16.7, 5.5 Hz, 1H), 2.99 (dd, *J* = 16.7, 8.4 Hz, 1H), 2.18 (dt, *J* = 13.5, 7.4 Hz, 3H), 2.08 – 1.92 (m, 5H), 1.86 – 1.77 (m, 1H), 1.65 – 1.54 (m, 2H), 1.41 – 1.25 (m, 18H), 0.96 (t, *J* = 7.5 Hz, 3H), 0.90 (t, *J* = 6.8 Hz, 3H).

^13^C NMR* (DEPT-Q, 126 MHz, MeOD) δ = 196.4, 175.7, 174.6, 162.4, 138.6, 131.0, 131.0, 115.7, 55.1, 42.9, 37.2, 33.1, 31.0, 30.5, 30.4, 30.4, 30.2, 28.3, 28.3, 27.2, 25.9, 23.9, 14.6, 10.6.

* Partial hemiacetal formation at the ketoamide in CD_3_OD observed. Only the NMR signals of the product are displayed.

HRMS (ESI): calc. for C_26_H_45_N_2_O_5_ [M+H]^+^: m/z = 465.3323, found: 465.3322.

^1^H NMR* (700 MHz, MeOD) δ 5.86 (ddd, *J* = 17.3, 10.4, 5.6 Hz, 1H), 5.36 – 5.33 (m, 2H), 5.19 (dt, *J* = 17.2, 1.4 Hz, 1H), 5.11 (dt, *J* = 10.4, 1.4 Hz, 1H), 4.91-4.86 (m, 1H), 4.33 (dd, *J* = 8.0, 5.1 Hz, 1H), 3.21 (dd, *J* = 16.7, 5.4 Hz, 1H), 2.99 (dd, *J* = 16.7, 8.4 Hz, 1H), 2.18 – 2.15 (m, 2H), 2.05 – 2.01 (m, 4H), 2.00 – 1.94 (m, 1H), 1.85 – 1.78 (m, 1H), 1.63 – 1.55 (m, 2H), 1.38 – 1.25 (m, 21H), 0.96 (t, *J* = 7.4 Hz, 3H), 0.90 (t, *J* = 7.1 Hz, 3H).

^13^C NMR* (DEPT-Q, 176 MHz, MeOD) δ = 196.3, 175.7, 174.5, 162.4, 138.6, 131.0, 131.0, 115.7, 55.1, 48.9, 42.9, 37.2, 33.2, 31.0, 31.0, 30.8, 30.6, 30.5, 30.4, 30.4, 28.3, 28.3, 27.2, 25.9, 23.9, 14.6, 10.6.

* Partial hemiacetal formation at the ketoamide in CD_3_OD observed. Only the NMR signals of the product are displayed.

HRMS (ESI): calc. for C_28_H_49_N_2_O_5_ [M+H]^+^: m/z = 493.3636, found: 493.3635.

^1^H NMR* (500 MHz, MeOD) δ 5.86 (ddd, *J* = 17.4, 10.4, 5.5 Hz, 1H), 5.37 – 5.31 (m, 2H), 5.19 (dt, *J* = 17.2, 1.5 Hz, 1H), 5.11 (dt, *J* = 10.5, 1.4 Hz, 1H), 4.91-4.86 (m, 1H), 4.33 (dd, *J* = 8.0, 5.1 Hz, 1H), 3.21 (dd, *J* = 16.7, 5.4 Hz, 1H), 2.99 (dd, *J* = 16.7, 8.4 Hz, 1H), 2.21 – 2.12 (m, 2H), 2.10 – 1.89 (m, 6H), 1.85 – 1.75 (m, 1H), 1.64 – 1.52 (m, 2H), 1.42 – 1.22 (m, 28H), 0.96 (t, *J* = 7.5 Hz, 3H), 0.90 (t, *J* = 6.9 Hz, 3H).

^13^C NMR* (DEPT-Q, 126 MHz, MeOD) δ = 196.4, 175.7, 174.6, 162.4, 138.6, 131.0, 129.2, 115.7, 55.1, 48.9, 42.9, 37.2, 33.2, 31.0, 30.9, 30.9, 30.9, 30.8, 30.8, 30.6, 30.6, 30.6, 30.5, 30.5, 30.5, 30.4, 28.3, 28.3, 27.2, 27.2, 27.1, 26.7, 25.9, 25.9, 23.9, 14.6, 10.6.

* Partial hemiacetal formation at the ketoamide in CD_3_OD observed. Only the NMR signals of the product are displayed.

HRMS (ESI): calc. for C_32_H_57_N_2_O_5_ [M+H]^+^: m/z = 549.4262, found: 549.4261.

^1^H NMR* (500 MHz, MeOD) δ 5.86 (ddd, *J* = 16.5, 10.4, 5.5 Hz, 1H), 5.40 – 5.29 (m, 4H), 5.19 (d, *J* = 17.2 Hz, 1H), 5.11 (d, *J* = 10.4 Hz, 1H), 4.90 – 4.85 (m, 1H), 4.33 (dd, *J* = 8.0, 5.1 Hz, 1H), 3.21 (dd, *J* = 16.7, 5.4 Hz, 1H), 2.99 (dd, *J* = 16.6, 8.4 Hz, 1H), 2.78 (t, *J* = 6.6 Hz, 2H), 2.21 – 2.14 (m, 2H), 2.12 – 2.02 (m, 4H), 2.02 – 1.90 (m, 1H), 1.87 – 1.76 (m, 1H), 1.60 (dt, *J* = 10.5, 7.0 Hz, 2H), 1.41 – 1.26 (m, 16H), 0.96 (t, *J* = 7.5 Hz, 3H), 0.91 (t, *J* = 6.8 Hz, 3H).

^13^C NMR* (DEPT-Q, 126 MHz, MeOD) δ = 196.4, 175.7, 174.6, 162.4, 138.6, 131.1, 131.0, 131.0, 129.2, 129.2, 115.7, 55.1, 48.9, 42.9, 42.7, 37.2, 32.8, 30.9, 30.6, 30.5, 30.4, 28.3, 28.3, 27.2, 26.7, 25.9, 25.9, 23.8, 14.6, 10.6.

* Partial hemiacetal formation at the ketoamide in CD_3_OD observed. Only the NMR signals of the product are displayed.

HRMS (ESI): calc. for C_28_H_47_N_2_O_5_ [M+H]^+^: m/z = 491.3480, found: 491.3478.

^1^H NMR* (600 MHz, MeOD) δ 5.86 (ddd, *J* = 17.3, 10.5, 5.6 Hz, 1H), 5.41 – 5.27 (m, 6H), 5.19 (dt, *J* = 17.2, 1.4 Hz, 1H), 5.11 (dt, *J* = 10.5, 1.3 Hz, 1H), 4.91 – 4.85 (m, 1H), 4.37 – 4.31 (m, 1H), 3.21 (dd, *J* = 16.6, 5.4 Hz, 1H), 2.99 (dd, *J* = 16.6, 8.4 Hz, 1H), 2.83 – 2.79 (m, 4H), 2.17 (t, *J* = 7.5 Hz, 2H), 2.12 – 2.04 (m, 4H), 2.02 – 1.93 (m, 1H), 1.81 (dtt, *J* = 15.2, 9.0, 5.9 Hz, 1H), 1.64 – 1.56 (m, 3H), 1.40 – 1.27 (m, 10H), 0.97 (2x t, *J* = 7.6 Hz, 6H).

^13^C NMR* (DEPT-Q, 151 MHz, MeOD) δ = 196.4, 175.7, 174.6, 162.4, 138.6, 132.9, 131.2, 129.4, 129.3, 129.0, 128.4, 115.7, 55.1, 48.9, 42.9, 37.2, 37.2, 30.9, 30.5, 30.4, 30.4, 28.3, 27.2, 26.7, 26.6, 25.9, 21.6, 14.8, 10.6.

* Partial hemiacetal formation at the ketoamide in CD_3_OD observed. Only the NMR signals of the product are displayed.

HRMS (ESI): calc. for C_28_H_45_N_2_O_5_ [M+H]^+^: m/z = 489.3323, found: 489.3321.

^1^H NMR* (500 MHz, MeOD) δ 5.88 (ddd, *J* = 17.3, 10.4, 5.6 Hz, 1H), 5.44 – 5.32 (m, 8H), 5.21 (dt, *J* = 17.2, 1.5 Hz, 1H), 5.13 (dt, *J* = 10.4, 1.3 Hz, 1H), 4.93 – 4.87 (m, 1H), 4.35 (dd, *J* = 8.1, 5.1 Hz, 1H), 3.23 (dd, *J* = 16.7, 5.5 Hz, 1H), 3.01 (dd, *J* = 16.7, 8.4 Hz, 1H), 2.85 (dq, *J* = 11.7, 6.0, 5.6 Hz, 6H), 2.25 – 2.19 (m, 2H), 2.16 – 2.06 (m, 5H), 2.04 – 1.90 (m, 2H), 1.83 (dt, *J* = 13.9, 7.6 Hz, 1H), 1.72 – 1-64 (m, 2H), 1.43 – 1.28 (m, 7H), 0.98 (t, *J* = 7.5 Hz, 3H), 0.93 (t, *J* = 6.9 Hz, 3H).

^13^C NMR* (DEPT-Q, 126 MHz, MeOD) δ = 194.8, 173.9, 173.0, 160.8, 137.1, 129.8, 128.8, 128.3, 128.1, 127.8, 127.8, 127.5, 127.4, 114.3, 53.6, 47.4, 41.4, 35.2, 31.3, 29.1, 26.8, 26.3, 25.6, 25.2, 25.2, 24.3, 22.2, 13.0, 9.0.

* Partial hemiacetal formation at the ketoamide in CD_3_OD observed. Only the NMR signals of the product are displayed.

HRMS (ESI): calc. for C_30_H_47_N_2_O_5_ [M+H]^+^: m/z = 515.3480, found: 515.3478.

^1^H NMR* (600 MHz, MeOD) δ 5.86 (ddd, *J* = 17.2, 10.4, 5.5 Hz, 1H), 5.42 – 5.27 (m, 12H), 5.20 (dt, *J* = 17.2, 1.4 Hz, 1H), 5.12 – 5.08 (m, 1H), 5.02 (tt, *J* = 10.3, 1.5 Hz, 1H), 4.91 – 4.85 (m, 1H), 4.33 (dd, *J* = 8.0, 5.1 Hz, 1H), 3.21 (dd, *J* = 16.8, 5.6 Hz, 1H), 3.00 (dd, *J* = 16.7, 8.2 Hz, 1H), 2.90 – 2.80 (m, 10H), 2.43 – 2.33 (m, 2H), 2.24 – 2.19 (m, 2H), 2.11 – 2.05 (m, 2H), 2.01 – 1.90 (m, 1H), 1.86 – 1.76 (m, 1H), 0.97 (t, *J* = 7.5 Hz, 3H).

^13^C NMR* (DEPT-Q, 151 MHz, MeOD) δ = 196.4, 174.9, 174.6, 162.4, 138.5, 133.0, 130.3, 129.6, 129.4, 129.4, 129.4, 129.3, 129.3, 129.3, 129.1, 128.3, 115.8, 55.1, 48.9, 42.9, 37.1, 26.7, 26.7, 26.7, 26.7, 26.6, 25.9, 24.9, 21.7, 14.8, 10.6.

* Partial hemiacetal formation at the ketoamide in CD_3_OD observed. Only the NMR signals of the product are displayed.

HRMS (ESI): calc. for C_32_H_47_N_2_O_5_ [M+H]^+^: m/z = 539.3480, found: 539.3478.

To a solution of ketoamide **S9** (85.0 mg, 0.248 mmol, 1.0 eq.) in MeOH (2 mL) was added sodium borohydride (10.3 mg, 0.273 mmol, 1.1 eq.) at 0 °C. The mixture was stirred for 1 h at 0 °C, subsequently diluted with EtOAc (10 mL), and quenched by the addition of sat. NH_4_Cl_aq_ (10 mL). The phases were separated and the aqueous phase was extracted with EtOAc (3 x 10 mL). The combined organic phases were washed with brine (10 mL), dried over Na_2_SO_4_, filtered and concentrated *in vacuo*. Purification by column chromatography (silica gel, 25-50% EtOAc in cyclohexane) afforded α-hydroxyamides **(*R*)-S19** (28.2 mg, 33%) and **(*S*)-S19** (24.4 mg, 29%) was colorless oils.

^1^H NMR (700 MHz, CDCl_3_) δ 5.79 (ddd, *J* = 16.7, 10.6, 5.8 Hz, 1H), 5.22 (d, *J* = 17.6 Hz, 1H), 5.14 (d, *J* = 10.6 Hz, 1H), 4.52 (td, *J* = 7.5, 5.3 Hz, 1H), 4.18 (dd, *J* = 7.5, 5.4 Hz, 1H), 3.74 (s, 3H), 2.10 (dt, *J* = 14.4, 5.4 Hz, 1H), 1.94 – 1.87 (m, 1H), 1.86 – 1.71 (m, 3H), 1.44 (s, 9H), 0.94 (t, *J* = 7.5 Hz, 3H).

^13^C NMR (DEPT-Q, 176 MHz, CDCl_3_) δ = 173.5, 172.4, 156.2, 137.6, 115.6, 80.2, 69.7, 53.3, 52.7, 52.2, 40.5, 28.3, 25.4, 9.7.

HRMS (ESI): calc. for C_16_H_28_N_2_O_6_Na [M+Na]^+^: m/z = 367.1839, found: 367.1839.

^1^H NMR (700 MHz, CDCl_3_) δ 7.39 (d, *J* = 8.4 Hz, 1H), 5.84 (ddt, *J* = 17.3, 10.1, 5.0 Hz, 1H), 5.23 (dd, *J* = 17.3, 1.7 Hz, 1H), 5.16 (dd, *J* = 10.6, 1.7 Hz, 1H), 4.74 (d, *J* = 9.0 Hz, 1H), 4.59 – 4.52 (m, 1H), 4.42 – 4.35 (m, 1H), 3.75 (s, 3H), 2.00 – 1.68 (m, 5H), 1.44 (s, 9H), 0.92 (t, *J* = 7.5 Hz, 3H).

^13^C NMR (DEPT-Q, 176 MHz, CDCl_3_) δ = 173.1, 172.6, 157.3, 137.4, 115.5, 80.6, 68.6, 52.8, 52.7, 52.2, 40.6, 28.3, 25.7, 9.6.

HRMS (ESI): calc. for C_16_H_28_N_2_O_6_Na [M+Na]^+^: m/z = 367.1839, found: 367.1839.

Synthesized according to general procedure A.

^1^H NMR (500 MHz, MeOD) δ 5.79 (ddd, *J* = 17.2, 10.4, 6.9 Hz, 1H), 5.22 (dt, *J* = 17.2, 1.4 Hz, 1H), 5.15 (dt, *J* = 10.4, 1.3 Hz, 1H), 4.63 – 4.55 (m, 1H), 4.35 (dd, *J* = 7.6, 5.2 Hz, 1H), 4.04 (dd, *J* = 9.4, 3.6 Hz, 1H), 2.17 (dd, *J* = 8.2, 6.9 Hz, 2H), 2.00 – 1.89 (m, 2H), 1.82 – 1.73 (m, 2H), 1.60 (p, *J* = 7.2 Hz, 2H), 1.35 – 1.25 (m, 20H), 0.96 (t, *J* = 7.5 Hz, 3H), 0.90 (t, *J* = 7.0 Hz, 3H).

^13^C NMR (DEPT-Q, 126 MHz, MeOD) δ 176.7, 175.2, 174.7, 138.3, 116.4, 70.1, 54.4, 50.2, 40.1, 37.1, 32.9, 30.6, 30.6, 30.5, 30.4, 30.3, 30.3, 30.1, 26.9, 25.9, 23.6, 14.2, 10.0.

HRMS (ESI): calc. for C_24_H_45_N_2_O_5_ [M+H]^+^: m/z = 441.3323, found: 441.3322.

Synthesized according to the general procedure A.

**Table S2.** Comparison of ^1^H NMR data of both synthesized diastereomers of myxoglucamide B to the data of the isolated sample from the literature.^[12]^

| No | δ_H_ *Lit.* | δ_H_ **2**^a^ | Δ_H_ | δ_H_ **35^a^** | Δ_H_ |
| --- | --- | --- | --- | --- | --- |
| 1 | 0.93^b^ | 0.93^b^ | 0 | 0.93^b^ | 0 |
| 2 | 0.93^b^ | 0.93^b^ | 0 | 0.93^b^ | 0 |
| 3 | 1.90^b^ (m) | 2.00 – 1.86^b^ (m, 3H) | - | 1.97 – 1.87^b^ (m, 3H) | - |
| 4 | 3.49 (m) | 3.49 (dt, 7.0, 4.2) | 0 | 3.49 (dt, 7.0, 4.3) | 0 |
| 5 | 1.49 (m) | 1.48 (m) | ‒0.01 | 1.48^b^ (m) | ‒0.01 |
| 6 | 1.35 (m) | 1.36 – 1.26^b^ (m, 16H) | - | 1.39 – 1.22^b^ (m, 16H) | - |
| 7-13^b^ | 1.36 – 1.26^b^ (m) | 1.36 – 1.26^b^ (m, 16H) | 0 | 1.39 – 1.22^b^ (m, 16H) | 0 |
| 14 | 1.60 (m) | 1.59 (m) | ‒0.01 | 1.63 (m) | +0.03 |
| 15 | 2.17 (t, 7.6) | 2.18 (t, 7.5) | +0.01 | 2.25 (t, 7.5) | **+0.08** |
| 16 | C=O | | | C=O | |
| 17 | NH | | | NH | |
| 18 | 4.58 (m) | 4.59 (m) | +0.01 | 4.66 (m) | **+0.08** |
| 19a | 1.97^b^ (m) | 2.00 – 1.86^b^ (m, 3H) | - | 1.97 – 1.87^b^ (m, 3H) | - |
| 19b | 1.77^b^ (m) | 1.82 – 1.74^b^ (m, 2H) | - | 1.71 (ddd, 14.1, 10.6, 3.4) | **‒0.06** |
| 20 | 4.03 (dd, 9.5, 3.4) | 4.04 (dd, 9.5, 3.7) | +0.01 | 4.02 (dd, 10.6, 2.5) | ‒0.01 |
| 21 | C=O | | | C=O | |
| 22 | NH | | | NH | |
| 23 | 4.31 (m) | 4.35 (dd, 7.6, 5.2) | +0.04 | 4.37 (dd, 7.6, 5.2) | **+0.06** |
| 24 | C=O | | | C=O | |
| 25 | OH | | | OH | |
| 26 | 5.79 (ddd, 17.3, 10.5, 18) | 5.79 (ddd, 17.1, 10.3, 6.9) | 0 | 5.84 (ddd, 17.2, 10.4, 5.4) | **+0.05** |
| 27a | 5.23 (dt, 17.0, 1.4) | 5.23 (dt, 17.2, 1.4) | 0 | 5.17 (dt, 17.2, 1.5) | **‒0.06** |
| 27b | 5.15 (dt, 10.3, 1.2) | 5.15 (dt, 10.3, 1.3) | 0 | 5.09 (dt, 10.4, 1.5) | **‒0.06** |
| 28a | 1.92^b^ (m) | 2.00 – 1.86^b^ (m, 3H) | - | 1.97 – 1.87^b^ (m, 3H) |  |
| 28b | 1.78^b^ (m) | 1.82 – 1.74^b^ (m, 2H) | - | 1.78 (m) | 0 |
| 29 | 0.93^b^ | 0.93^b^ | 0 | 0.93^b^ | 0 |
| 1‘ | 4.30 (d, 7.6) | 4.30 (d, 7.8) | 0 | 4.30 (d, 7.8) | 0 |
| 2‘ | 3.17 (dd, 9.0, 8.2) | 3.18 (dd, 8.8, 7.7) | +0.01 | 3.18 (dd, 8.9, 7.7) | +0.01 |
| 3‘ | 3.33 (m) | 3.36 – 3.28^b^ (m, 2H) | - | 3.36 – 3.29 (m, 2H) | - |
| 4‘ | 3.30 (m) | 3.36 – 3.28^b^ (m, 2H) | - | 3.36 – 3.29 (m, 2H) | - |
| 5' | 3.22 (m) | 3.23 (m) | +0.01 | 3.23 (m) | +0.01 |
| 6’a | 3.85 (dd, 11.7, 2.4) | 3.85 (dd, 11.7, 2.5) | 0 | 3.85 (dd, 11.6, 2.5) | 0 |
| 6‘b | 3.68 (dd, 11.6, 5.6) | 3.68 (dd, 11.7, 5.5) | 0 | 3.68 (dd, 11.6, 5.5) | 0 |

^a^ acquired at 500 MHz in CD_3_OD; ^b^ overlapping signals.

| No | δ_C_ *Lit.* | δ_C_ **2**^a^ | Δ_C_ | δ_C_ **35^a^** | Δ_C_ |
| --- | --- | --- | --- | --- | --- |
| 1 | 17.9 | 17.8 | -0.1 | 17.9 | 0 |
| 2 | 18.3 | 18.2 | -0.1 | 18.2 | -0.1 |
| 3 | 31.5 | 31.6 | +0.1 | 31.6 | +0.1 |
| 4 | 84.4 | 84.9 | +0.5 | 85.0 | +0.6 |
| 5 | 31.7 | 31.8 | +0.1 | 31.8 | +0.1 |
| 6 | 26.6 | 26.3 | -0.3 | 26.3 | -0.3 |
| 7-13^b^ | 31.2 – 29.6 | 30.6 – 30.0 | - | 30.7 – 30.1 | - |
| 14 | 26.8 | 26.8 | 0 | 26.9 | +0.1 |
| 15 | 36.9 | 37.0 | +0.1 | 37.0 | +0.1 |
| 16 | 175.2 | 174.5 | -0.7 | 174.7 | -0.5 |
| 17 | NH | | | NH | |
| 18 | 50.1 | 50.1 | 0 | 49.1 | **-1.0** |
| 19 | 39.8 | 40.0 | +0.2 | 40.5 | **+0.7** |
| 20 | 69.9 | 70.0 | +0.1 | 70.0 | +0.1 |
| 21 | 176.5 | 176.6 | +0.1 | 176.6 | +0.1 |
| 22 | NH | | | NH | |
| 23 | 54.8 | 54.2 | -0.6 | 54.1 | **-0.7** |
| 24 | 175.4 | 175.0 | -0.4 | 176.1 | **+0.7** |
| 25 | OH | | | OH | |
| 26 | 138.2 | 138.2 | 0 | 139.3 | **+1.1** |
| 27 | 116.4 | 116.3 | -0.1 | 114.6 | **-1.8** |
| 28 | 25.9 | 25.7 | -0.2 | 25.8 | -0.1 |
| 29 | 9.8 | 9.9 | +0.1 | 9.9 | +0.1 |
| 1‘ | 103.6 | 103.7 | +0.1 | 103.7 | +0.1 |
| 2‘ | 75.2 | 75.2 | 0 | 75.2 | 0 |
| 3‘ | 78.0 | 78.0 | 0 | 78.0 | 0 |
| 4‘ | 71.5 | 71.6 | +0.1 | 71.6 | 0 |
| 5' | 77.4 | 77.4 | 0 | 77.4 | 0 |
| 6’ | 62.6 | 62.7 | +0.1 | 62.8 | +0.2 |

**Table S3.** Comparison of ^13^C NMR data of both synthesized diastereomers of myxoglucamide B to the data of the isolated sample from the literature.^[12]^

^a^ acquired as DEPT-Q at 126 MHz in CD_3_OD.

^b^ overlapping signals

**2**: HRMS (ESI): calc. for C_32_H_59_N_2_O_11_ [M+H]^+^: m/z = 647.4114, found: 647.4112.

**35**: HRMS (ESI): calc. for C_32_H_59_N_2_O_11_ [M+H]^+^: m/z = 647.4114, found: 647.4112.

To a solution of the 4-((tert-butoxycarbonyl)amino)hex-5-enoic acid (50.0 mg, 0.218 mmol, 1.0 eq.) and methyl (*S*)-2-aminobutanoate hydrochloride (40.2 mg, 0.262, 1.2 eq.) in CH_2_Cl_2_, were added HATU (124  mg, 0.327, 1.5 eq.), and DIPEA (95.2 µL, 0.545 mmol, 2.5 eq.). The mixture was stirred overnight at room temperature and was then diluted with EtOAc. The organic phase was washed with 1 M HCl, sat. NaHCO_3_, and brine, dried over Na_2_SO_4_ and concentrated *in vacuo*. Purification by flash column chromatography (silica gel, 40% EtOAc in cyclohexane) afforded amide **S20** (52.1 mg, 73%) as a mixture of diastereomers (d.r. 1:1).

^1^H NMR (700 MHz, CDCl_3_) δ 5.78 – 5.69 (m, 1H), 5.15 (2x d*, *J* = 17.3 Hz, 1H), 5.08 (dd, *J* = 10.5, 1.7 Hz, 1H), 4.81 – 4.68 (m, 1H), 4.54 – 4.48 (m, 1H), 3.72 (2x s*, 3H), 2.30 – 2.27 (m, 2H), 1.94 – 1.82 (m, 2H), 1.78 – 1.68 (m, 2H), 1.41 (s, 9H), 0.90 (2x t*, *J* = 7.5 Hz, 3H).

* Overlapping Signals

^13^C NMR (DEPT-Q, 176 MHz, CDCl_3_) δ = 172.9, 172.3, 155.7, 138.2, 114.9, 79.5, 53.4, 53.3, 52.2, 32.7, 30.7, 28.3, 25.4, 9.5.

HRMS (ESI): calc. for C_16_H_29_N_2_O_5_ [M+H]^+^: m/z = 329.2071, found: 329.2070.

Synthesized according to general procedure A. **36** was isolated as a mixture of diastereomers (d.r. 1:1).

^1^H NMR (500 MHz, MeOD) δ 5.79 (ddd, *J* = 17.3, 10.5, 6.0 Hz, 1H), 5.17 (2x dt*, *J* = 17.1, 1.5 Hz, 1H), 5.10 (2x dt*, *J* = 10.4, 1.4 Hz, 1H), 4.42 – 4.33 (m, 1H), 4.29 (dt, *J* = 8.4, 5.0 Hz, 1H), 2.32 – 2.26 (m, 2H), 2.21 (t, *J* = 7.5 Hz, 2H), 1.92 – 1.83 (m, 2H), 1.81 – 1.68 (m, 2H), 1.65 – 1.58 (m, 2H), 1.36 – 1.25 (m, 20H), 0.98 (2x t*, *J* = 7.4 Hz, 3H), 0.90 (t, *J* = 6.9 Hz, 3H).

* Overlapping signals

^13^C NMR (DEPT-Q, 126 MHz, MeOD) δ = 176.0, 175.9, 175.7, 175.6, 139.5, 139.4, 115.7, 115.6, 55.3, 55.3, 52.7, 52.6, 37.4, 37.4, 33.6, 33.5, 33.2, 31.9, 31.7, 30.9, 30.9, 30.9, 30.8, 30.6, 30.6, 30.5, 30.5, 27.3, 27.3, 26.0, 26.0, 23.9, 14.6, 10.8, 10.8.

HRMS (ESI): calc. for C_24_H_45_N_2_O_4_ [M+H]^+^: m/z = 425.3374, found: 425.3373.

A solution of amine hydro­chloride **15** (25.0 mg, 0.073 mmol, 1.0 eq.) in MeOH (1.5 mL) was treated with Pd on activated charcoal (10% Pd basis, 15.5 mg, 0.015 mmol, 0.2 eq.). The atmosphere was exchange with hydrogen by three times evacuating and subsequently purging with hydrogen. The reaction mixture was stirred under hydrogen atmosphere for 30 min. Subsequently, the mixture was filtered through Celite and concentrated *in vacuo*. The crude product **S21** was used without further purification. The following amide coupling and global deprotection were performed according to general procedure A.

^1^H NMR (500 MHz, MeOD) δ 4.33 (dd, *J* = 8.0, 5.2 Hz, 1H), 4.30 (d, *J* = 7.8 Hz, 1H), 4.19 (dtt, *J* = 15.9, 8.2, 5.1 Hz, 1H), 3.85 (dd, *J* = 11.7, 2.5 Hz, 1H), 3.68 (dd, *J* = 11.7, 5.5 Hz, 1H), 3.49 (dt, *J* = 7.0, 4.3 Hz, 1H), 3.37 – 3.31 (m, 2H), 3.25 – 3.20 (m, 1H), 3.18 (dd, *J* = 8.9, 7.8 Hz, 1H), 3.11 (dd, *J* = 16.1, 4.9 Hz, 1H), 2.83 (dd, *J* = 16.1, 8.3 Hz, 1H), 2.14 (t, *J* = 7.5 Hz, 2H), 2.02 – 1.86 (m, 3H), 1.84 – 1.78 (m, 1H), 1.66 – 1.54 (m, 3H), 1.54 – 1.40 (m, 4H), 1.39 – 1.24 (m, 16H), 1.00 – 0.86 (m, 12H).

^13^C NMR (DEPT-Q, 126 MHz, MeOD) δ = 197.0, 176.2, 162.5, 104.1, 85.4, 78.4, 77.8, 75.6, 72.0, 63.2, 55.1, 48.7, 43.3, 37.3, 32.2, 32.0, 31.1, 30.9, 30.9, 30.9, 30.8, 30.6, 30.4, 28.9, 28.8, 27.3, 26.8, 25.9, 18.6, 18.2, 11.0, 10.6.

HRMS (ESI): calc. for C_32_H_59_N_2_O_11_ [M+H]^+^: m/z = 647.4114, found: 647.4112.

^1^H NMR (600 MHz, DMSO) δ 8.43 (d, *J* = 7.9 Hz, 1H), 7.62 (d, *J* = 8.2 Hz, 1H), 4.14 – 4.09 (m, 1H), 4.08 – 3.96 (m, 1H), 2.99 (dd, *J* = 16.2, 5.4 Hz, 1H), 2.75 (dd, *J* = 16.2, 8.0 Hz, 1H), 2.03 – 1.94 (m, 2H), 1.87 – 1.78 (m, 1H), 1.77 – 1.68 (m, 1H), 1.50 – 1.33 (m, 4H), 1.30 – 1.16 (m, 20H), 0.85 (2x t, *J* = 7.2 Hz, 6H), 0.81 (t, *J* = 7.4 Hz, 3H).

^13^C NMR (151 MHz, DMSO) δ = 196.6, 172.6, 171.7, 160.9, 53.4, 46.2, 42.2, 35.4, 31.3, 29.0, 29.0, 29.0, 28.9, 28.7, 28.7, 28.5, 27.2, 25.3, 23.8, 22.1, 13.9, 10.3, 10.3.

HRMS (ESI): calc. for C_24_H_45_N_2_O_5_ [M+H]^+^: m/z = 441.3323, found: 441.3323.

# 3 Activity-based Protein Profiling

## 3.1 ABPP in A549

1. Seeding A549 cells in a T75 flask with media (gibco DMEM with GlutaMAX + 10% gibco FBS) and incubation at 37 °C and 5% CO_2_ until the cell were confluent
2. Removal of media and washing with PBS (2 x 10 mL)
3. Treatment of cells with Accutase (1.5 mL) and incubation at 37 °C for 3 min
4. Addition of 8.5 mL media and transfer into a falcon tube
5. Centrifugation (5 min, 1000 rpm)
6. Removal of supernatants and resuspension with media (10 mL)
7. Cell counting:
   a. 20 µL of cell suspension mixed with 20 µL trypan blue
   b. Cell counting performed on Cedex XS with ibidi plates: 8.6∙10^6^ (cell viability: 97%)
8. Centrifugation (5 min, 1000 rpm)
9. Removal of supernatant media and resuspension with PBS (5 mL each)
10. Centrifugation (5 min, 1000 rpm) and removal of supernatant PBS
11. Resuspension of the cell pellets in 0.1% Triton^TM^ X-100 (Sigma-Aldrich T8787) (400 µL) in ultra pure water and protease inhibitor (Pierce^TM^ A32963) (100 µL) in ultra pure water and distribution into protein LoBind® tubes (Eppendorf) (50 µL each)
12. Cell lysis for 20 min on ice with vortexing every 10 min
13. Addition of the activity-based probe **16** (2.5 µL from 10 mM stock solution) to the cell lysates (final concentration 500 µM) or DMSO (2.5 µL) (PanReac AppliChem, A3672) as a negative control
14. Incubation of the tubes for 3 h at 25 °C and 300 rpm in an Eppendorf ThermoMixer C
15. Preparation of the Streptavidin Sepharose^TM^ beads (cytiva, 17511301)
    a. Resuspension of the beads by shaking the vial
    b. Transfer of the bead suspension (50 µL) to different 1.5 mL protein LoBind® tubes (Eppendorf) (for each sample one tube)
    c. Suspension of the beads in 0.4% SDS solution in PBS (1 mL)
    d. Centrifugation (2 min, 4000 rpm)
    e. Removal of the supernatants
    f. Repetition of washing step three times
16. Transfer of each cell lysate to different tubes containing the streptavidin beads
17. Addition of 0.4% SDS solution in PBS (400 µL)
18. Vortexing the samples and incubation for 1 h at RT in a rotary mixer
19. Centrifugation (2 min, 4000 rpm)
20. Removal of the supernatants
21. Resuspension of the beads, centrifugation (2 min, 4000 rpm) and removal of the supernatants in each of the following washing steps:
    a. 3 × 0.4% SDS solution in PBS (1 ml)
    b. 2 × 3 M urea in ultra pure water (1 mL)
    c. 2 × PBS (1 mL)
22. Addition of a 2% SDS solution in ultra pure water (50 µL) to the tubes
23. Heating the samples for 6 min at 96 °C in an Eppendorf ThermoMixer C
24. Centrifugation (2 min, 13000 rpm)
25. Transfer of the supernatants to Pierce^TM^ Spin Columns (Thermo Scientific, 69725), placed in new protein LoBind® tubes (Eppendorf)
26. Centrifugation for few seconds
27. Preparing the SpeedBeads magnetic carboxylate modified particles (GE45152105050250)
    a. Resuspension thoroughly on an orbital shaker for half a day
    b. Transfer 4 mL of the suspension to a 5 mL protein LoBind® tube (Eppendorf) and let beads sediment on a magnet
    c. Remove the glycerin storage solution and wash three times with ultra pure water
    d. Transfer 30 µL by height to a 1.5 mL protein LoBind® tubes (Eppendorf) and add 1 mL ultra pure water
    e. Store in the fridge at 4 °C
28. Reduction with 50 mM TCEP in ultra pure water (5 µL) for 1 h at 55 °C and 700 rpm in an Eppendorf ThermoMixer C
29. Cooling to RT for around 15 min
30. Alkylation with 200 mM MMTS in isopropanol (2.5 µL) for 10 min at RT and 700 rpm in an Eppendorf ThermoMixer C
31. Resuspension of the magnetic beads from step 28. by vortexing
32. Addition of magnetic bead solution (20 µL) to every sample, followed by MeCN (200 µL)
33. Incubation over night at 22 °C and 1000 rpm in an Eppendorf ThermoMixer C
34. Centrifugation (5 min, 5000 rpm)
35. Placement of a magnet at the side of the tube and transfer of the supernatant into new 1.5 mL protein LoBind® tubes (Eppendorf)
36. Addition of magnetic bead suspension (10 µL) to the supernatants
37. Incubation for 7 h at 22 °C and 1000 rpm in an Eppendorf ThermoMixer C
38. Resuspension of the beads from step 36. in EtOH (300 µL)
39. Incubated for 7 h at 22 °C and 1000 rpm in an Eppendorf ThermoMixer C
40. Centrifugation (5 min, 5000 rpm) of all tubes
41. Tubes with 20 µL beads: discarding of supernatants
42. Tubes with 10 µL beads: discarding of supernatants, resuspension in EtOH (300 µL) and addition to the tubes with the 20 µL beads
43. Incubation for 15 min at 22 °C and 1000 rpm in an Eppendorf ThermoMixer C
44. Centrifugation (5 min, 5000 rpm)
45. Discarding of supernatants, resuspension in EtOH (300 µL), and incubation for 15 min at 22 °C and 1000 rpm in an Eppendorf ThermoMixer C
46. Centrifugation (5 min, 5000 rpm)
47. Discarding of supernatants, resuspension in MeCN (300 µL), and incubation for 15 min at 22 °C and 1000 rpm in an Eppendorf ThermoMixer C
48. Centrifugation (5 min, 5000 rpm)
49. Discarding of supernatants and drying of samples for 15 min with open lids in the fume hood
50. Resuspension in 1 M TEAB buffer (50 µL) pH 8.5
51. Addition of 2 µL Trypsin (Promega, V5111) (0.2 µg/mL in Trypsin Buffer)
52. Incubation over night at 37 °C and 1000 rpm in an Eppendorf ThermoMixer C
53. Resuspension in MeCN (1 mL) and addition of magnetic bead solution (10 µL)
54. Incubation over night at 22 °C and 1000 rpm in an Eppendorf ThermoMixer C
55. Centrifugation (5 min, 5000 rpm)
56. Discarding of supernatants, resuspension in MeCN (200 µL), and incubation for 15 min at 22 °C and 1000 rpm in an Eppendorf ThermoMixer C
57. Centrifugation (5 min, 5000 rpm)
58. Discarding of supernatants, resuspension in MeCN (200 µL), and incubation for 15 min at 22 °C and 1000 rpm in an Eppendorf ThermoMixer C
59. Centrifugation (5 min, 5000 rpm)
60. Discarding of supernatants and drying tubes with an open lid in the fume hood for 30 min
61. Peptide elution

a. Addition of 2% DMSO in ultra pure water (20 µL) and resuspension of the beads

b. Incubation for 15 min at 22 °C and 1000 rpm in an Eppendorf ThermoMixer C

c. Centrifugation (5 min, 5000 rpm)

d. Transfer of the supernatants to new 1.5 mL protein LoBind® tubes (Eppendorf)

e. Resuspension of the beads in ultra pure water (20 µL)

f. Incubation for 15 min at 22 °C and 1000 rpm in an Eppendorf ThermoMixer C

g. Centrifugation (5 min, 5000 rpm)

h. Transfer of the supernatants to the tubes from step d.

i. Removing volatiles at a CentriVap Concentrator (Labconco) overnight at 23 °C

For MS/MS analyses, the peptides were loaded onto Evotips as described by the supplier Evosep:

1. Wash dry Evotips with 20 µL solvent B and centrifuge at 800 g for 60 s
2. Soak in 100 µL 2-propanol until all the tips are pale white
3. Equilibrate soaked Evotips with 20 µL solvent A and centrifuge at 800 g for 60 s
4. Load samples on wet tips (20 µL in solvent A) and centrifuge for 60 s at 800 g
5. Wash tips with 20 µL solvent A and centrifuge for 60 s at 800 g
6. Transfer 100 µL solvent A and centrifuge tips for 10 s at 800 g to keep tips wet

The peptides were eluted afterwards over an Evosep One HPLC connected to a time-of-flight mass spectrometer (timsTOF Pro, Bruker, Bremen, Germany). Two independent ABPP experiments with a total of five positive controls containing activity-based probe **16** and five negative controls containing DMSO were performed. Identification of the enriched peptides was done using the Peaks Studio 12 software with the following settings.

Search parameters:

- Parent Mass Error Tolerance: 20.0 ppm
- Fragment Mass Error Tolerance: 0.05 Da
- Precursor Mass Search Type: monoisotopic
- Enzyme: Trypsin
- Max Missed Cleavages: 2
- Digest Mode: Semi-Specific
- Fixed Modifications: Beta-methylthiolation: 45.99
- Variable Modifications: Oxidation: 15.99
- Max Variable PTM Per Peptide: 2
- Database: 2024-08-20_Human_database_homo_sapiens
- Taxonomy: All species
- Searched Entry: 20436
- Contaminant Database: N/A
- Deep Learning Boost: yes
- Peptide FDR: 1.0%
- Proteins log_10_P ≥ 15.0
- Protein Unique Peptides ≥ 1
- Denovo Only ALC ≥ 50.0%
- Denono Only Tag Sharing: Disabled
- Denovo Only Fully Matched: No
- Confident Amino Acid Threshold: 0.00%
- Instrument parameters: Ion source: ESI(nano-spray)
- Fragmentation Mode: CID, CAD (y and b ions)
- MS Scan Mode: TimsTOF
- MS/MS Scan Mode: TimsTOF

Protein abundance was examined by label free quantification (LFQ) by adding up the areas under the curve of each feature (specific m/z, RT, charge, − log_10_P); this was done for all peptides belonging to a given protein. The results of the MS/MS analysis are shown in Table S4. These data were used for the statistical analysis, and the results are depicted in a volcano plot in the manuscript in Figure 2C.

Statistical analysis of the results was performed as follows. Identiﬁed proteins and their respective abundances have been exported from Peaks Studio 12 pro as a .csv ﬁle format and transformed to a .txt ﬁle format via Excel 2016. With this ﬁle, a generic matrix upload was done in Perseus 2.1.0.0. A categorical annotation was done assigning the ten samples (n = 10) to five DMSO negative controls (n = 5) and five positive controls containing the activity-based probe **16** (n = 5). The data were then log_2_ transformed and missing values were replaced with the constant 0. A two-tailed t-test with a false discovery rate of FDR = 0.05 was carried out. The thereby calculated −log_10_(P values) were plotted in GraphPad Prism 10.3.0 against the calculated diﬀerences in abundance between the positive control (activity-based probe **16**) and the negative control (DMSO) as log_2_(positive control/negative control) that corresponds to log_2_(fold change).

**Table S4.** List of enriched proteins from ABPP.

| **Protein** | **log_2_ (fold change)** | **−log_10_(p)** |
| --- | --- | --- |
| **AL1A1\|** Aldehyde dehydrogenase 1A1 | 14.44 | 7.30 |
| **TBA1C\|** Tubulin alpha-1C chain | 11.09 | 6.02 |
| **TBA3C\|** Tubulin alpha-3C chain | 11.06 | 5.99 |
| **TBA3D\|** Tubulin alpha-3D chain | 11.06 | 5.99 |
| **TBA1B\|** Tubulin alpha-1B chain | 11.06 | 5.99 |
| **TBA1A\|** Tubulin alpha-1A chain | 11.06 | 5.99 |
| **TBA3E\|** Tubulin alpha-3E chain | 10.64 | 5.48 |
| **TBA4A\|** Tubulin alpha-4A chain | 10.60 | 5.40 |
| **TBA8\|** Tubulin alpha-8 chain | 10.60 | 5.40 |
| **KRT86\|** Keratin, type II cuticular Hb6 | 10.54 | 7.04 |
| **KRT81\|** Keratin, type II cuticular Hb1 | 10.54 | 7.04 |
| **KR87P\|** Putative keratin-87 protein | 10.31 | 7.32 |
| **KRT83\|** Keratin, type II cuticular Hb3 | 10.31 | 7.32 |
| **AKR1C3\|** Aldo-keto reductase 1C3 | 7.99 | 7.86 |
| **AKR1C2\|** Aldo-keto reductase 1C2 | 7.87 | 2.19 |
| **KRT85\|** Keratin, type II cuticular Hb5 | 7.24 | 2.35 |
| **ALDH2\|** Aldehyde dehydrogenase, mitochrondrial | 7.10 | 2.01 |
| **TBA4B\|** Putative tubulin-like protein alpha-4B | 6.79 | 2.26 |
| **NOTC4\|** Neurogenic locus notch homolog protein 4 | 6.58 | 1.39 |
| **Q61726\|** Keratin, type II | 6.44 | 2.35 |
| **AKR1B10\|** Aldo-keto reductase 1B10 | 6.13 | 2.20 |
| **TITIN\|** Titin | 6.06 | 0.81 |
| **PRDX1\|** Peroxiredoxin-1 | 5.93 | 1.21 |
| **SSBP\|** Single-stranded DNA-binding protein | 5.70 | 1.59 |
| **G6PD\|** Glucose-6-phosphate 1-dehydrogenase | 5.42 | 1.30 |
| **ANXA2\|** Annexin A2 | 5.24 | 0.96 |
| **TBB5\|** Tubulin beta chain | 5.16 | 1.19 |
| **TBB4B\|** Tubulin beta-4B chain | 5.07 | 1.17 |
| **TBB2A\|** Tubulin beta-2A chain | 5.07 | 1.17 |
| **TBB2B\|** Tubulin beta-2B chain | 5.07 | 1.17 |
| **TBAL3\|** Tubulin alpha chain-like 3 | 4.91 | 1.37 |
| **AKR1C1\|** Aldo-keto reductase 1C1 | 4.90 | 1.38 |
| **TBB3\|** Tubulin beta-3 chain | 4.89 | 1.14 |
| **EF1A1\|** Elongation factor 1-alpha 1 | 4.68 | 0.85 |
| **EF1A3\|** Putative elongation factor 1-alpha-like 3 | 4.68 | 0.85 |
| **PRDX1\|** Peroxiredoxin-2 | 4.40 | 0.85 |
| **AKR1D1\|** Aldo-keto reductase 1D1 | 4.39 | 1.38 |
| **AKR1B1\|** Aldo-keto reductase 1B1 | 4.17 | 0.85 |
| **PCBP1\|** Poly(rC)-binding protein 1 | 4.13 | 0.85 |
| **TKT\|** Transketolase | 4.12 | 0.85 |
| **KRT82\|** Keratin, type II cuticular Hb2 | 4.06 | 1.33 |

## 3.2 Competitive ABPP

A competitive ABPP was performed using the same protocol as described above with the following exceptions:

- A549 cell lysate (5.5∙10^6^ cells per sample, 98% cell viability) was incubated with **1** (n = 3, 200 µm) or DMSO (n = 3) for 30 min at 25 °C and 300 rpm
- afterwards activity-based probe **16** (100 µm) was added to all samples and the samples were incubated for 3 h at 25 °C and 300 rpm

The statistical analysis was performed as described above. The log_2_ transformed area under the curve of each previously identified significantly enriched proteins is displayed in figure 2D comparing the sample with and without pre-incubation with **1**.

**Scheme S1.** Enzyme reactions catalyzed by the identified oxidoreductases aldehyde dehydrogenase (AL1A1), aldo-keto reductase 1B10 (AKR1B10), aldo-keto reductase 1C2 (AKR1C2) and aldo-keto reductase 1C3 (AKR1C3).

# 4 Enzyme Inhibition Assays

## 4.1 Aldehyde Dehydrogenase (ALDH) Inhibition Assay

An aldehyde dehydrogenase inhibitor screening kit (Sigma-Aldrich, MAK327) was used to examine the inhibitory efficacy of myxoglucamide A on ALDH.

1. Dilution of recombinant ALDH (from yeast, Sigma-Aldrich: A6338) to 2.2 U/mL (5.5 mg/mL) with assay buffer
2. Further dilution to 0.22 U/mL (0.55 mg/mL) with a 18:1 mixture of assay buffer and DTT solution (c = 160 µM 🡪 final DTT concentration: 8 µM)
3. Serial dilution of myxoglucamide A with DMSO: 10 mM – 0.05 mM
4. Addition of ALDH solution (0.55 mg/mL, 47.5 µL) to all needed wells in a 96 well plate and addition of Assay Buffer to the background well
5. Addition of myxoglucamide A dilution series (2.5 µL) to the sample wells and DMSO (2.5 µL) to the vehicle and background well
6. Incubation for 6 h at 37 °C (plate is covered with foil and lid to reduce evaporation)
7. Dilution of 4X substrate solution to 1X with ultrapure water
8. Preparation of reaction mix (50 µL needed for each well)
   a. Mix for inhibitor/vehicle wells: 45 µL assay buffer, 8 µL NAD/MTT, 1 µL diaphorase, 1 µL substrate solution (1X)
   b. Mix for background wells: 45 µL assay buffer, 8 µL NAD/MTT, 1 µL diaphorase
9. Addition of reaction mixes (50 µL) to the respective wells
10. Measurement of absorbance at λ = 565 nm every minute for 60 min with a Tecan Plate Reader Infinite M200 Pro

For analysis of the results, the mean value of the background wells was subtracted from all measured values of the inhibitor and vehicle wells. The resulting values were plotted against the measuring time using GraphPad Prism 10.3.0. The slope of the initial linear range (0 – 8 min) of the curves was determined and normalized (Vehicle = 100%, Y = 0: 0%). These values as triplicates for each concentration were plotted against the inhibitor concentration during incubation. A nonlinear regression fit with GraphPad Prism 10.3.0 ([Inhibitor] vs normalized response – Variable Slope) yielded the IC_50_ value. Disulfiram was used as a positive control and the values are consistent with the product information sheet provided for the assay kit (Sigma Aldrich, MAK327, IC_50_ = 2.65 µm).

**Figure S1.** Aldehyde dehydrogenase inhibition assay with different incubation times: 1 h (left) and 6 h (right).

## 4.2 Aldo-Keto-Reductase 1 B1/B10 Inhibition Assay

1. Recombinant AKR1B1 (Hölzel Diagnostics: NKM-ALR0901-100μg) and AKR1B10 (Hölzel Diagnostics: NKM-ATGP0314-100μg) were diluted with assay buffer (0.1 M sodium phosphate pH 6.0) to a concentration of 20 μg/mL
2. DL-Glyceraldehyde (100 mM stock) was diluted with assay buffer to 20 mM or 1 mM
3. NADPH (10 mM stock) was diluted with assay buffer to 0.64 mM
4. Serial dilution of myxoglucamide A with DMSO: 10 mM – 1.25 mM
5. Addition of AKR1B1/B10 (47.5 μL) to all wells except background
6. Addition of assay buffer (50 μL) to the background well
7. Addition of myxoglucamide A dilution series (2.5 μL) to the sample wells (final concentration: 250 – 31.25 μM) and DMSO (2.5 μL) to the vehicle control wells
8. Addition of NADPH solution (25 μL) to all wells (final concentration: 160 μM)
9. Shaken for 10 min in an orbital plate shaker
10. Addition of glyceraldehyde (25 μL, 1 mM) to AKR1B1 wells (final concentration: 0.25 mM)
11. Addition of glyceraldehyde (25 μL, 20 mM) to AKR1B10 wells (final concentration: 5 mM)
12. Measurement of fluorescence (excitation: 340 nm, emission: 460 nm) with a Tecan Plate Reader Infinite M200 Pro every 30 seconds for 15 minutes

For analysis of the results, all measured values of the inhibitor and vehicle wells were subtracted from the background wells. The resulting values were plotted against the measuring time using GraphPad Prism 10.3.0. The slope of the initial linear range of the curves was determined and normalized (Vehicle = 100%, Y = 0: 0%). These values as triplicates for each concentration were plotted against the inhibitor concentration. A nonlinear regression fit with GraphPad Prism 10.3.0 ([Inhibitor] vs normalized response – Variable Slope) yielded the IC_50_ value.

**Figure S2.** Inhibitory effect of **1** on aldo-keto reductase 1B1 (left) and 1B10 (right).

## 4.3 Aldo-Keto-Reductase 1 C1/C2/C3 Inhibition Assay

1. Recombinant AKR1C1 (R&D Systems: 6529-DH-020), AKR1C2 (Biomol ABS-PP-4447.100) and AKR1C3 (Hölzel Diagnostics: NKM-ATGP0270-100μg or self-prepared) were diluted with assay buffer (50 mM Tris buffer + 500 mM NaCl pH 8.5) to 10 μg/mL
2. (S)-Tetrahydronaphthol (200 mM stock in DMSO) was diluted with substrate buffer (50 mM Tris buffer + 500 mM NaCl + 20% (v/v) EtOH, pH 8.5) to a concentration of 4 mM
3. NADP^+^ (50 mM stock in H2O) was diluted with substrate buffer to 2 mM
4. Serial dilution of myxoglucamide A or inhibitors in DMSO
5. Addition of AKR1C1/C2/C3 (47.5 μL) to all wells except background
6. Addition of assay buffer (50 μL) to the background well
7. Addition of myxoglucamide A/ inhibitor dilution series (2.5 μL) to the sample wells and DMSO (2.5 μL) to the vehicle control wells
8. Addition of NADP^+^ solution (25 μL) to all wells (final concentration: 0.5 mM)
9. Addition of tetrahydronaphthol solution (25 μL) to all wells (final concentration: 1 mM)
10. Measurement of fluorescence (excitation: 340 nm, emission: 460 nm) with a Tecan Plate Reader Infinite M200 Pro every 30 seconds for 15 minutes

For analysis of the results, the mean value of the background wells was subtracted from all measured values of the inhibitor and vehicle wells. The resulting values were plotted against the measuring time using GraphPad Prism 10.3.0. The slope of the initial linear range of the curves was determined and normalized (Vehicle = 100%, Y = 0: 0%). These values as triplicates for each concentration were plotted against the inhibitor concentration. A nonlinear regression fit with GraphPad Prism 10.3.0 ([Inhibitor] vs normalized response – Variable Slope) yielded the IC_50_ value. As a positive control flufenamic acid was used and the results were in accordance with the literature.^[43]^

**Figure S3.** Inhibitory effect of **1** on aldo-keto reductase 1C1 – C3.

# 5 AKR1C3 Expression and Purification

An expression plasmid with the human AKR1C3 cDNA (NM_003739.6) in a pET-28a(+) vector with *N*-terminal His-tag and TEV-site was purchased (AKR1C3_OHu15813C_pET-28a(+)-TEV, Genscript). BL21(DE3) CodonPlus RIL cells carrying the plasmid were grown in 2 L TB medium. Protein expression was induced with 1 mM IPTG, followed by shaking over night at 20 °C. Cells were harvested by centrifugation and stored at −20°C. Cells were resuspended in Buffer A (25 mM sodium phosphate, pH 8.0, 150 mM NaCl) and lysed by sonification. The lysate was centrifuged and the supernatant was loaded onto a 5 mL HisTrap column. The column was washed with 50 mM imidazole in Buffer A, followed by elution with a 50 mM to 500 mM imidazole gradient. The AKR1C3 peak fractions were pooled and further purified, without TEV digest, on a Superdex 200 26/60 gelfiltration column in Buffer C (10 mM potassium phosphate pH 7.0, 1 mM EDTA, 1 mM DTT). Pooled peak fractions were concentrated to 16 g/L by ultrafiltration. The protein was stored in 100 µL aliquots at −70°C.

# 6 Structure-Activity Relationship AKR1C3

AKR1C3 inhibition assays with the synthesized myxoglucamide analogs were performed using the procedure described above with the expressed recombinant AKR1C3.

**Figure S4.** Structure-activity relationship study for the inhibition of AKR1C3.

# 7 Cytotoxicity

1. Seeding A549 cells in a T75 flask with media (gibco DMEM with GlutaMAX + 10% gibco FBS) and incubation at 37 °C and 5% CO_2_ until the cell were confluent
2. Removal of media and washing with PBS (2 x 10 mL)
3. Treatment of cells with Accutase (1.5 mL) and incubation at 37 °C for 3 min
4. Addition of 8.5 mL media and transfer into a falcon tube
5. Centrifugation (5 min, 1000 rpm)
6. Removal of supernatants and resuspension with media (10 mL)
7. Cell counting:
   a. 20 µL of cell suspension mixed with 20 µL trypan blue
   b. Cell counting performed on Cedex XS with ibidi plates
8. Dilution of cell suspension to a concentration of 50 000 cells/mL
9. Addition of 198 µL of cell suspension and 2 µL inhibitor solution (10 mM in DMSO) to the first well in each row in a 96-well plate
10. Addition of 100 µL of cell suspension (incl. 1% DMSO) to the rest of the wells in each row
11. Addition of 100 µL of media to the last well in each row (background well)
12. Serial Dilution (100 µL) from first well until the penultimate well with cell suspension
13. Incubation for 72 h at 37 °C and 5% CO_2_
14. Addition of Alamar Blue reagent (Resazurin) to each well (5 µL)
15. Incubation for 4 h at 37 °C and 5% CO_2_
16. Measurement of fluorescence (excitation: 540 nm, emission: 600 nm) with a Tecan Plate Reader Infinite M200 Pro

For analysis of the results, the mean value of the background wells was subtracted from all measured values of the inhibitor and vehicle wells. The resulting values were normalized (Vehicle = 100% cell viability, Y = 0: 0%). These values as triplicates for each concentration were plotted against the inhibitor concentration. A nonlinear regression fit with GraphPad Prism 10.3.0 ([Inhibitor] vs normalized response – Variable Slope) yielded the CC_50_ value.

**Figure S5.** Cytotoxic effect of myxoglucamide and its analogs on A549 cells.

# 8 Michaelis-Menten Enzyme Kinetics AKR1C3

1. Recombinant AKR1C3 was diluted with assay buffer (50 mM Tris buffer + 500 mM NaCl pH 8.5) to 10 μg/mL
2. NADP^+^ (50 mM stock in H2O was diluted with substrate buffer (50 mM Tris buffer + 500 mM NaCl + 20% (v/v) EtOH, pH 8.5) to a concentration of 4 mM
3. Serial dilution of (*S*)-tetrahydronaphthol (200 mM stock in DMSO) with substrate buffer
4. Serial dilution of myxoglucamide A or inhibitors in DMSO
5. Addition of AKR1C3 (47.5 μL, final assay concentration: 132 nM) to all wells except background
6. Addition of assay buffer (50 μL) to the background well
7. Addition of myxoglucamide A/ inhibitor dilution series (2.5 μL) to the sample wells and DMSO (2.5 μL) to the vehicle control wells
8. Addition of NADP^+^ solution (25 μL) to all wells (final concentration: 0.5 mM)
9. Addition of tetrahydronaphthol (25 μL) 🡪 each substrate concentration was added to each inhibitor concentration
10. Measurement of fluorescence (excitation: 340 nm, emission: 460 nm) with a Tecan Plate Reader Infinite M200 Pro every 30 seconds for 15 minutes

For analysis of the results, the mean value of the background wells was subtracted from all measured values of the inhibitor and vehicle wells. The resulting values were plotted against the measuring time using GraphPad Prism 10.3.0. The slope of the initial linear range of the curves was determined. These values were plotted against the tetrahydronaphthol concentration for each inhibitor concentration. A nonlinear regression fit with GraphPad Prism 10.3.0 using the “Michaelis-Menten” model yielded the respective K_M_ and V_max_ values for each replicate in the absence of inhibitor. For the weak-binding inhibitor **1**, the K_i_ value was determined by globally fitting the substrate-dependent initial velocity using the “Competitive Inhibition” model. Conversely, for the tight-binding inhibitors **24**, **26** and **30**, initial velocities were plotted against the inhibitor concentration for each individual substrate concentration. These datasets were globally fitted to the “Morrison K_i_” model, using the K_M_ values determined from the Michaelis-Menten fit as a constant constraint. All experiment were performed in duplicates for each inhibitor and the mean ± SEM was calculated.

**Table S5.** Summary of enzyme kinetics measurements with inhibitor **1**, **24**, **26** and **30**. The parameters K_i_, K_M_ and V_max_ are listed for both replicates and K_i_ also as duplicates.

| **#** | Replicate A | | | Replicate B | | | Duplicate |
| --- | --- | --- | --- | --- | --- | --- | --- |
|  | K_i_  [nM] | K_M_  [mM] | V_max_  [RFU/s] | K_i_  [nM] | K_M_  [mM] | V_max_  [RFU/s] | K_i_  [nM]^a^ |
| **1** | 337^[b]^ | 0.231 | 53.2 | 248^[b]^ | 0.269 | 49.7 | 293 ± 45 |
| **24** | 45.6^[c]^ | 0.207 | 56.7 | 32.0^[c]^ | 0.337 | 71.2 | 38.8 ± 6.8 |
| **26** | 20.0^[c]^ | 0.245 | 53.8 | 20.8^[c]^ | 0.212 | 21.0 | 20.4 ± 0.4 |
| **30** | 23.8^[c]^ | 0.245 | 20.5 | 21.0^[c]^ | 0.235 | 29.2 | 22.4 ± 1.4 |

^[a]^ K_i_ = mean ± SEM; ^[b]^ determined using the Competitive Inhibition model; ^[c]^ determined using the Morrison K_i_ model.

**Figure S6.** Enzyme kinetics of AKR1C3 inhibition. Fitting of the dataset was performed with GraphPad Prism 10.3.0 using the Competitive Inhibition model for compound **1** and the Morrison Ki model for compounds **24**, **26** and **30**.

# 9 Thermal Shift Assay (nanoDSF)

## 9.1 Measurement of Denaturation Temperature (T_M_)

Recombinant AKR1C3 was diluted with assay buffer (50 mM Tris + 500 mM NaCl pH 8.5) to a concentration of 0.45 mg/mL (~12 µM). 48 µL of protein solution was mixed with 1 µL inhibitor solution (10 mM in DMSO, final concentration: 200 µM) and 1 µL NADP^+^ (10 mM in H_2_O, final concentration: 200 µM) or 1 µL H_2_O. T_M_ values were measured on a Prometheus Panta (NanoTemper Technologies GmbH). Samples in triplicates were heated from 25 °C to 95 °C at a rate of 1.5 °C/min, the intrinsic fluorescence of aromatic residues up on unfolding of the proteins (350/330 nm fluorescence ratio) was measured and the inflection point of the resulting curve was determined as the T_M_ value.

**Table S6.** T_M_ values of binary and ternary complexes of AKR1C3, inhibitors and NADP^+^

| **Compound** | **T_M_ (AKR1C3 + Inhibitor)** | **T_M_ (AKR1C3 + Inhibitor + NADP^+^)** |
| --- | --- | --- |
| **1** | 56.6 | 61.2 |
| **2** | 53.2 | 55.4 |
| **20** | 56.6 | 61.4 |
| **21** | 53.3 | 54.8 |
| **22** | 53.9 | 56.5 |
| **23** | 56.5 | 60.9 |
| **24** | 56.7 | 62.1 |
| **25** | 53.2 | 58.1 |
| **26** | 57.6 | 62.7 |
| **27** | 53.7 | 59.1 |
| **28** | 50.8 | 54.9 |
| **29** | 56.5 | 61.7 |
| **30** | 55.4 | 63.0 |
| **31** | 54.4 | 59.8 |
| **32** | 55.4 | 60.7 |
| **33** | 53.6 | 55.0 |
| **34** | 50.0 | 53.8 |
| **35** | 53.9 | 57.0 |
| **36** | 50.6 | 54.5 |
| **37** | 57.2 | 62.2 |
| **38** | 57.3 | 61.4 |

AKR1C3 (0.45 mg/mL, ~12 µM): T_M_ = 53.4 °C

AKR1C3 + 200 µM NADP^+^: T_M_ = 54.9 °C

## 9.2 Binding Affinity Determination

Recombinant AKR1C3 was diluted in assay buffer (50 mM Tris + 500 mM NaCl pH 8.5) to a concentration of 0.5 mg/mL (~13 µM). For binary complex binding affinity determination, a serial dilution of **1** (2000 – 3.9 µM, 2% DMSO) or inhibitor (500 – 1 µM, 5% DMSO) was prepared. For ternary complex binding affinity determination, a serial dilution of **1** (2000 – 1 µM, 2% DMSO, 2 mM NADP^+^) or inhibitor (500 – 0.25 µM, 5% DMSO, 2 mM NADP^+^) was prepared. Thermal shift assays were measured on a Prometheus Panta (NanoTemper Technologies GmbH). Samples in triplicates were heated from 25 °C to 95 °C at a rate of 1.5 °C/min and the intrinsic fluorescence of aromatic residues upon unfolding of the proteins (350/330 nm fluorescence ratio) was measured. The fluorescence curves were analyzed using the FoldAffinity web server.^[33, 34]^ The heat capacity change upon unfolding (ΔC_p_) was fitted from the fluorescence ratio curves using the Global_CP fitting mode. The fluorescence curves described by Eq. (2-5) were locally fitted to the measured data using the previously calculated ΔC_p_ values. From these fits, the unfolded protein fractions were calculated at each temperatures and ligand concentrations. At a selected temperature (57 °C for the binary complex and 64 °C for the ternary complex) close to the denaturation point, the fraction unfolded was plotted against the ligand concentration. Eq. 6 derived from the unfolding and binding equilibrium model (Eq. 1), was fitted to these data points, and the dissociation constant was determined from the resulting curve.

(1)

**Equation 1.** Equilibrium model of unfolding and binding process. FL = protein (folded) – ligand complex, K_d_ = dissociation constant at equilibrium, K_U_ = unfolding constant at equilibrium, F = free folded protein, L = free ligand, U = unfolded protein.^[33]^

$F\left( T \right)= f_{U}\left( m_{U}T+b_{U} \right)+(1-f_{U})(m_{F}T+b_{F})$	(2)

**Equation 2.** Protein fluorescence. F = protein fluorescence, T = temperature, f_U_ = fraction unfolded, m_U_ = unfolded slope, b_U_ = unfolded intercept, m_F_ = folded slope, b_F_ = folded intercept.^[33]^

$$f_{U}=\frac{K_{U,obs}}{K_{U,obs}+1}$$

(3)

**Equation 3.** Relationship between unfolded fraction f_U_ and apparent unfolding constant K_U,obs_.^[33]^

$$K_{U,obs}=\frac{[U]}{\left[ F \right]+[FL]}=e^{-\frac{{\Delta G}_{U,obs}}{RT}}$$

(4)

**Equation 4.** Temperature dependence of unfolding constant. [U] = equilibrium concentration of unfolded protein, [F] = equilibrium concentration of folded protein, [FL] = equilibrium concentration of protein-ligand complex, ΔG_U,obs_ = apparent Gibbs energy of unfolding, R = gas constant.^[33]^

$${\Delta G}_{U,obs}={\Delta H}_{T_{M},obs}\left( 1-\frac{T}{T_{M,obs}} \right)-{\Delta C}_{p}\left( T_{M,obs}-T+ln\left( \frac{T}{T_{M,obs}} \right) \right)$$

(5)

**Equation 5.** Gibbs energy of unfolding. ΔH_U,obs_ = apparent unfolding enthalpy, T_M,obs_ = observed melting temperature, ΔC_p_ = head capacity change upon unfolding.^[33]^

$$f_{U}=\frac{K_{U}}{K_{U}+\frac{{[L]}_{0}-\left( \frac{\left[ L \right]_{0}+\frac{{[P]}_{0}}{K_{U}+1}+K_{d}}{2} \right)-\sqrt{\frac{\left( {[L]}_{0}+\frac{{[P]}_{0}}{K_{U}+1}+K_{d} \right)^{2}}{4}-\frac{{[L]}_{0}{[P]}_{0}}{K_{U}+1}}}{K_{d}}+1}$$

(6)

**Equation 6.** Unfolded fraction dependent on ligand concentration. f_U_ = unfolded fraction, K_U_ = unfolding constant at equilibrium, [L]_0_ = total ligand concentration (FL + L), [P]_0_ = total protein concentration ([U] + [F] + [FL]), K_d_ = dissociation constant at equilibrium.^[33]^

**Figure S7.** Determination of binding affinities for the binary (AKR1C3 + inhibitor) and the ternary complex ([AKR1C3 + NADP^+^] + inhibitor). (left): thermal unfolding curves at different inhibitor concentrations; (right): fraction of unfolded protein extracted from the thermal shift assay (using FoldAffinity web tool)^[33, 34]^ plotted against the ligand concentration.

# 10 X-Ray Crystal Structure of AKR1C3 with NADP^+^ and 1

## 10.1 Co-Crystallization

AKR1C3 (15 mg/mL) was incubated with 1 mM of NADP^+^ (100 mM stock in H_2_O) and 1 mM of **1** (100 mM stock in DMSO, 1% DMSO), at rt prior to crystallization. To remove precipitates, the solution was centrifuged. 200 nL protein solution and 200 nL crystallization buffer were pipetted into Intelli-Plate 96–3 LVR crystallization wells (Art Robbins Instruments, LLC, Sunnyvale, CA, USA) using a Crystal Gryphon liquid handling system (Art Robbins Instruments, LLC, Sunnyvale, CA, USA). In the same way 60 μL crystallization buffer were pipetted into the corresponding reservoir wells of the wellplate. Subsequently, the plates were sealed and stored in a Rock Imager 100 (Formulatrix, Bedford, MA, USA) at 18 °C. Initial crystallization conditions were identified using commercially available screens. For further optimization screens based on initial hit identification were prepared using Formulator 34 (Formulatrix, Inc., USA). AKR1C3-NADP^+^-**1** co-crystals formed after a few days in 29% PEG3350 and 0.2 M sodium acetate. The crystals were fished with a nylon loop from the crystallization buffer and put into a mixture of (*R*,*R*)-butane-2,3-diol (Thermo Scientific) and crystallization buffer (1:9) for cryo-protection. The crystals were flash-cooled in liquid nitrogen.

## 10.2 Data Collection, Refinement and Validation

Diffraction data of flash-cooled crystals were collected at 100 K on beamline P11 of the PETRA III synchrotron at DESY (Hamburg, Germany). Diffraction images were processed with the program package autoProc (v 1.0.5),^[44, 45]^ which uses parts of the CCP4 suite: The data was reduced and indexed with XDS.^[46, 47]^ Intensity scaling was performed and unit cell parameters were determined with Pointless (v 1.12.14)^[48, 49]^ and diffraction spots were integrated with Aimless (v 0.7.9).^[50, 51]^ The data was further refined with the Phenix suite program package (v 1.20.1):^[52, 53]^ Data quality was assessed using Xtriage, molecular replacement was performed with Phenix Phaser (v 2.8.3).^[54, 55]^ As phasing model, chain A from the asymmetric unit of 7WQR was used. A first model of the protein was fitted with Phenix Autobuild, then this model was refined by iteratively using Coot (v0.9.8.92)^[56, 57]^ and Phenix Refine. Ligand restraints were generated using phenix.elbow. Data collection and refinement statistics are listed in Table S7. Figures have been prepared with ChimeraX.

| **Structure** | AKR1C3 in complex with NADP+ and **1** |
| --- | --- |
| **PDB ID code** | 9SWH |
| **Data collection:** |  |
| Beamline | DESY P11 Petra III |
| Wavelength (Å) | 1.03320 |
| Space group | P1 |
| Cell dimensions: |  |
| *a, b, c* (Å) | 41.585, 52.932, 76.088 |
| *α, β, γ* (°) | 77.226, 85.027, 73.350 |
| Resolution (Å) | 49.691-1.991 (2.026 - 1.991) |
| *R*_merge_ (%) | 21.5 (123.8) |
| *R_pim_* (%) | 12.9 (78.3) |
| *I/σI* | 5.3 (1.3) |
| Completeness (%) | 69.3 (23.22) |
| Multiplicity | 3.8 (3.4) |
| CC1/2 (%) | 96.6 (39.6) |
| **Refinement:** |  |
| Resolution (Å) | 1.991 |
| No. reflections | 28691 |
| R_work_/R_free_ (%) | 23.62/28.65 |
| No. atoms (non-H) | 5481 |
| Protein | 5106 |
| Ligand/ion | 378 |
| Water | 177 |
| B-factors(Å^2^) | 18.46 |
| Protein | 18.48 |
| Ligand/ion | 18.77 |
| Water | 17.79 |
| R.m.s deviations |  |
| Bond lengths(Å) | 0.0072 |
| Bond angles(°) | 0.796 |
| Ramachandran statistics |  |
| Favored | 0.962 |
| Allowed | 3.64 |
| Outliers | 0.32 |
| Clashscore (MolProbity) | 4.44 |
| MolProbity score | 1.48 |

**Table S7.** X-ray data collection and refinement statistics.

**Figure S8.** X-ray crystal structure of AKR1C3 in complex with **1** and NADP^+^ (PDB: 9SWH). A) 2F_o_–F_c_ electron density map of **1** and NADP⁺ contoured at 1.0 σ, shown as blue mesh within the AKR1C3 surface of **chain A**; **1** in purple and NADP⁺ in yellow sticks. B) Superposition of **1** in **chain A** (purple) and **chain B** (green). C) F_o_-F_c_ difference electron density map of **1** in AKR1C3 **chain B**. The difference electron density is displayed as green mesh at a level of 2.5 σ before the incorporation of **1** into the model. D) F_o_-F_c_ difference electron density map of **1** in AKR1C3 **chain A**. The difference electron density is displayed as green mesh at a level of 2.5 σ before the incorporation of **1** into the model.

# 11 References

[12]	T. Wang, A. Popoff, M. Hunter, R. Garcia, A. Sikandar, R. Müller, C. Fu, **2025**.

[18]	J. A. Dale, D. L. Dull, H. S. Mosher, *J. Org. Chem.* **1969**, *34*, 2543–2549.

[33]	S. Niebling, O. Burastero, J. Bürgi, C. Günther, L. A. Defelipe, S. Sander, E. Gattkowski, R. Anjanappa, M. Wilmanns, S. Springer, H. Tidow, M. García-Alai, *Sci. Rep.* **2021**, *11*, 9572.

[34]	For calculation of K_d_ values from thermal shift assay the FoldAffinity WebTool was used: <https://spc.embl-hamburg.de/app/foldAffinity>.

[40]	G. R. Fulmer, A. J. M. Miller, N. H. Sherden, H. E. Gottlieb, A. Nudelman, B. M. Stoltz, J. E. Bercaw, K. I. Goldberg, *Organometallics* **2010**, *29*, 2176–2179.

[41]	Y. Perez-Riverol, J. Bai, C. Bandla, D. García-Seisdedos, S. Hewapathirana, S. Kamatchinathan, Deepti J. Kundu, A. Prakash, A. Frericks-Zipper, M. Eisenacher, M. Walzer, S. Wang, A. Brazma, Juan A. Vizcaíno, *Nucleic Acids Res.* **2022**, *50*, D543–D552.

[42]	C. Du, J. Fang, J. Chen, Z. Liu, H. Li, X. Wang, X. Xie, X. She, *Org. Lett.* **2019**, *21*, 8718–8721.

[43]	A. O. Adeniji, B. M. Twenter, M. C. Byrns, Y. Jin, M. Chen, J. D. Winkler, T. M. Penning, *J. Med. Chem.* **2012**, *55*, 2311–2323.

[44]	C. Vonrhein, C. Flensburg, P. Keller, A. Sharff, O. Smart, W. Paciorek, T. Womack, G. Bricogne, *Acta Crystallogr. Sect. D: Biol. Crystallogr.* **2011**, *67*, 293–302.

[45]	C. Vonrhein, C. Flensburg, P. Keller, A. Sharff, O. Smart, W. Paciorek, T. Womack, G. Bricogne, *autoProc, Program for Diffraction Image Processing*, Global Phasing Limited, Cambridge, U.K., **2022**.

[46]	W. Kabsch, *Acta Crystallogr. Sect. D: Biol. Crystallogr.* **2010**, *66*, 125–132.

[47]	W. Kabsch, *XDS, Program for Diffraction Image Indexing*, Max Planck Institute for Medical Research, Heidelberg, Germany, **2023**.

[48]	P. Evans, *Acta Crystallogr. Sect. D: Biol. Crystallogr.* **2006**, *62*, 72–82.

[49]	P. Evans, *POINTLESS, Program for Space Group Determination*, Laboratory of Molecular Biology, Cambridge, U.K., **2021**.

[50]	P. R. Evans, G. N. Murshudov, *Acta Crystallogr. Sect. D: Biol. Crystallogr.* **2013**, *69*, 1204–1214.

[51]	P. Evans, *AIMLESS. Program for Scaling & Analysis of Unmerged Intensities*, Laboratory of Molecular Biology, Cambridge, U.K., **2022**.

[52]	D. Liebschner, P. V. Afonine, M. L. Baker, G. Bunkoczi, V. B. Chen, T. I. Croll, B. Hintze, L.-W. Hung, S. Jain, A. J. McCoy, N. W. Moriarty, R. D. Oeffner, B. K. Poon, M. G. Prisant, R. J. Read, J. S. Richardson, D. C. Richardson, M. D. Sammito, O. V. Sobolev, D. H. Stockwell, T. C. Terwilliger, A. G. Urzhumtsev, L. L. Videau, C. J. Williams, P. D. Adams, *Acta Crystallogr. Sect. D: Biol. Crystallogr.* **2019**, *75*, 861–877.

[53]	P. D. Adams, P. V. Afonine, G. Bunkoczi, T. Burnley, V. B. Chen, Y. Dar, I. Davis, E. Draizen, N. Echols, R. Gildea, P. Gros, R. W. Grosse-Kunstleve, J. J. Headd, B. Hintze, L.-W. Hung, T. Ioerger, D. Liebschner, A. J. McCoy, E. McKee, N. W. Moriarty, R. D. Oeffner, B. K. Poon, R. J. Read, J. S. Richardson, D. C. Richardson, J. Sacchettini, N. Sauter, O. V. Sobolev, L. C. Storoni, T. C. Terwilliger, C. J. Williams, P. H. Zwart, *Phenix: Python-based Hierarchical Environment for Integrated Xtallography, Program for Crystal Structure Refinement*, Lawrence Berkeley Laboratory, Berkeley, CA, USA, **2022**.

[54]	A. J. McCoy, R. W. Grosse-Kunstleve, P. D. Adams, M. D. Winn, L. C. Storoni, R. J. Read, *J. Appl. Crystallogr.* **2007**, *40*, 658–674.

[55]	*PHENIX: Phaser, Program for Molecular Replacement*, Lawrence Berkeley Laboratory, Berkeley, CA, USA, **2022**.

[56]	P. Emsley, B. Lohkamp, W. G. Scott, K. Cowtan, *Acta Crystallogr. Sect. D: Biol. Crystallogr.* **2010**, *66*, 486–501.

[57]	P. Emsley, K. Cowtan, B. Lohkamp, *Coot. Marina Bay*, University of Oxford; University of York, Oxford, U.K.; York, U.K., **2012**.

# 12 NMR Spectra
